# Revisiting Homochiral *versus* Heterochiral Interactions through a Long Detective Story of a Useful Azobis-Nitrile and Puzzling Racemate

**DOI:** 10.1021/acs.cgd.3c00372

**Published:** 2023-06-27

**Authors:** Juan García de la Concepción, Mirian Flores-Jiménez, Louis A. Cuccia, Mark E. Light, Cristóbal Viedma, Pedro Cintas

**Affiliations:** † Department of Organic and Inorganic Chemistry, Faculty of Sciences, and IACYS-Green Chemistry and Sustainable Development Unit, University of Extremadura, E-06006 Badajoz, Spain; ‡ Department of Chemistry and Biochemistry, Concordia University, 7141 Sherbrooke Street West, H4B 1R6 Montreal, Canada; § Department of Chemistry, Faculty of Natural and Environmental Sciences, University of Southampton, Southampton SO17 1BJ, U.K.; ∥ Department of Crystallography and Mineralogy, University Complutense, 28040 Madrid, Spain

## Abstract

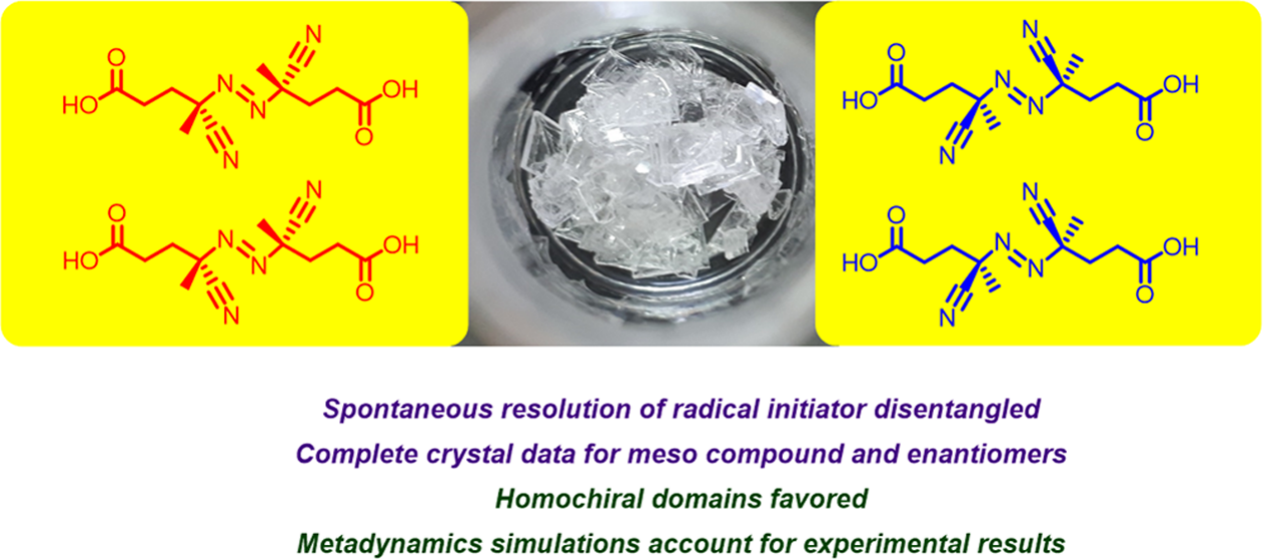

This paper documents and reinvestigates the solid-state and crystal structures of 4,4′-azobis-4-cyanopentanoic acid (ACPA), a water-soluble azobis-nitrile of immense utility as a radical initiator in living polymerizations and a labile mechanophore that can be embedded within long polymer chains to undergo selective scission under mechanical activation. Surprisingly, for such applications, both the commercially available reagent and their derivatives are used as “single initiators” when this azonitrile is actually a mixture of stereoisomers. Although the racemate and *meso* compounds were identified more than half a century ago and their enantiomers were separated by classical resolution, there have been confusing narratives dealing with their characterization, the existence of a conglomeratic phase, and fractional crystallization. Our results report on the X-ray crystal structures of all stereoisomers for the first time, along with further details on enantiodiscrimination and the always intriguing arguments accounting for the stability of homochiral *versus* heterochiral crystal aggregates. To this end, metadynamic (MTD) simulations on stereoisomer molecular aggregates were performed to capture the incipient nucleation events at the picosecond time scale. This analysis sheds light on the driving homochiral aggregation of ACPA enantiomers.

## Abstract 2

The solid-state behavior and crystal structures of all stereoisomers of ACPA, a useful azobis-nitrile-based radical initiator, have been described and discussed in depth. The investigation sheds light on complex and often confusing structural data collected so far and provides further reasoning on homochiral *versus* heterochiral aggregation of Pasteurian-type racemates.

## Introduction and Background

**Figure 1:**
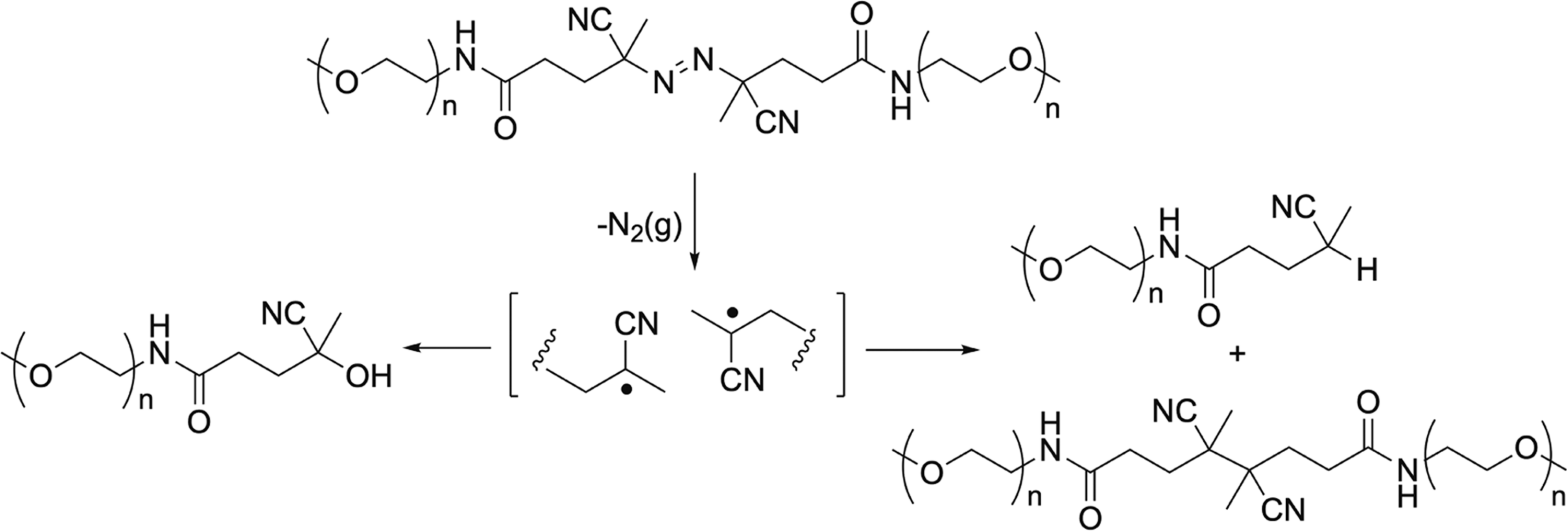
Azo mechanophore reports bond scission and repairing in polymers under mechanical stress.

Azonitriles are versatile substances in synthetic organic chemistry and polymer chemistry, a role well portrayed by azobisisobutyronitrile (AIBN) used as a foamer in plastics manufacture and as a radical initiator, soluble in a wide variety of organic solvents.^1,2^ Thermal decomposition of the monoazo function releases N_2_ and a stabilized cyanoradical, triggering subsequent addition and H-abstraction reactions. Water-soluble azonitriles would also be of enormous utility for practical applications. A suitable derivative to this end is 4,4′-azobis-4-cyanopentanoic (or cyanovaleric acid), hereinafter referred to as ACPA.^3^ Remarkably, the ACPA skeleton can easily be incorporated into the middle of a polymer chain where it can undergo selective fragmentation under the action of tensile forces, thus illustrating the mechanophore concept (Figure 1).^4,5^

**Figure 2:**
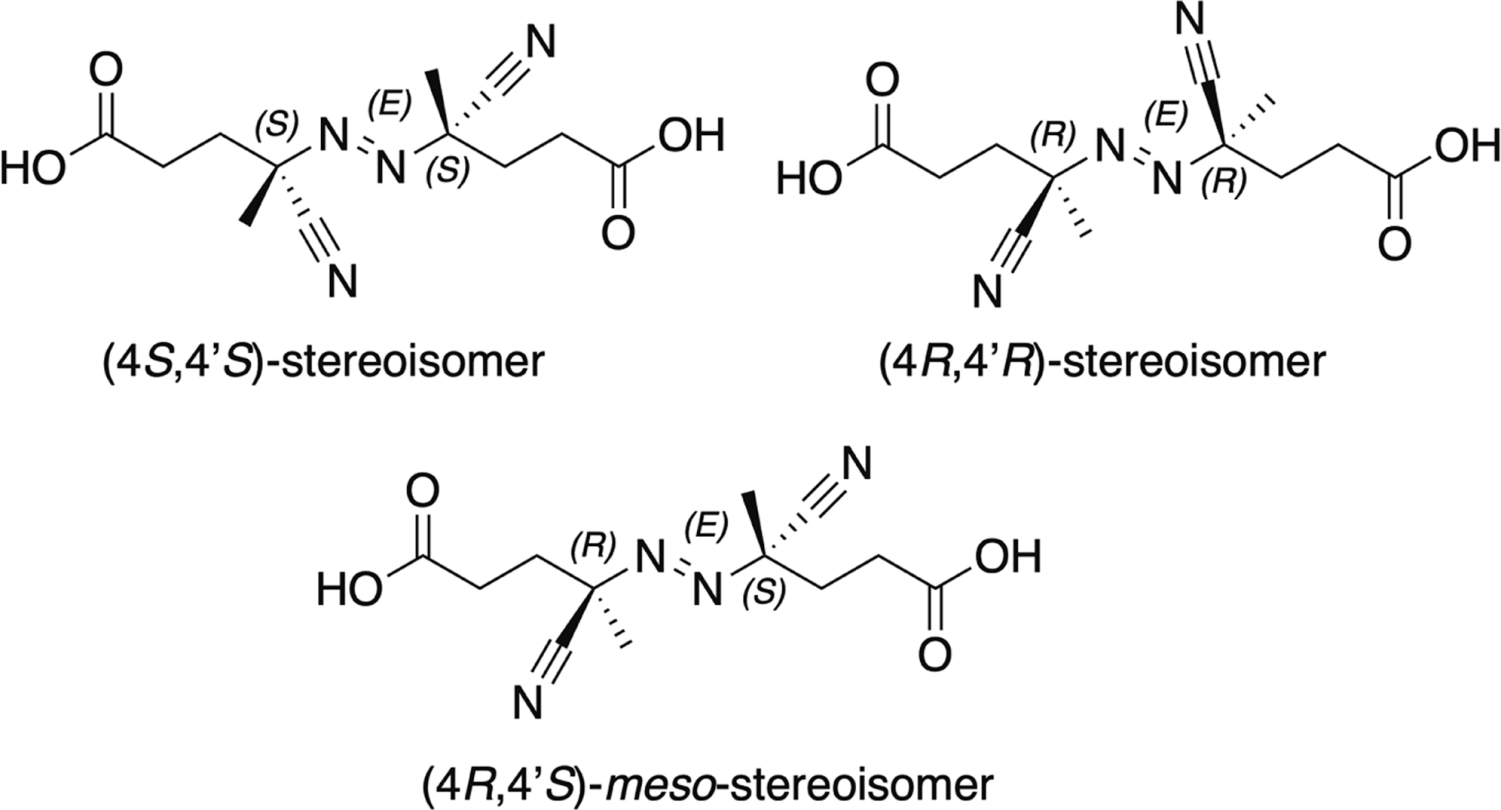
Configurational stereoisomers of 4,4′-azobis-4-cyanopentanoic acid (ACPA).

ACPA was prepared by Haines and Waters as early as 1955 by means of a Strecker-type synthesis that bypasses the use of hazardous HCN (otherwise employed in subsequent protocols).^6^ The authors recognized that, as a result of a symmetrical arrangement containing two stereogenic centers, the substance should exist in *meso*, racemic, and optically active forms (shown in Figure 2). They were able to separate the *meso* and racemic structures due to their difference in solubility in water and aqueous methanol, although failed to achieve the resolution of single enantiomers with either brucine or strychnine as chiral bases.

**Figure 3:**
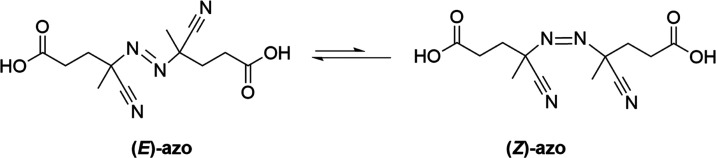
*E*,*Z*-isomerization of ACPA azo group.

A careful investigation in 1970 by Overberger and Labianca, who also explored both photochemical and thermal decompositions of such azonitriles, not only characterized the *meso* (mp 134–135 °C) and *d*,*l*-racemate (mp 117–118 °C) but also successfully resolved the latter with (−)-quinine in acetone, thereby affording the pure enantiomers ([α]_D_ +45.3 and −44.8 as maximum optical rotations) after acid treatment of the corresponding diastereomeric salts.^7^ Interestingly, the team noted the possibility of resolving the racemate by the preferential fractional crystallization of the (+)-isomer when hot aqueous solutions were allowed to cool slowly, yielding a mixture of a colorless solid (racemic form) and colorless platelets of the impure dextrorotatory isomer, which could be separated with tweezers in a Pasteurian-like approach. However, the reproducibility of this fractional resolution was somewhat capricious and dependent on the temperature and volume of solvent and resulted in a low yield of the dextrorotatory isomer. The discovery was presumably a matter of serendipity: “If the initial experiments had not been undertaken on warm days, when slow cooling of the hot solutions was easily facilitate, the recrystallization of impure racemic acid would have yielded simply purified racemic acid and not (+)-isomer”.^8^ While the authors noted the oddity of spontaneous resolutions by invoking the previous literature, they did not delve deeper. Nor did they realize this phenomenon could only be compatible with a conglomeratic phase, at least below the temperature of crystallization. The latter agrees with the higher melting point of an enantioenriched mixture relative to the racemate,^9,10^ a key point overlooked as well. It is surprising that a comprehensive patent in 1973, reporting the preparation of ACPA and related azonitriles,^11^ while quoting the method of Haines and Waters ignored, however, the resolution by Overberger and Labianca. Moreover, this study identified two ACPA isomers, one melting at 125–127 °C and the other at 141–143 °C, by taking advantage of their different solubility in ethanol and ethyl acetate, which were correlated with the low melting (110–111 °C) and the high melting (128 °C) isomers isolated by Haines and Waters, *i.e.*, *meso* and racemic acids, respectively. Since the azo function is susceptible to *cis*–*trans* isomerization, the authors hypothesized the existence of four isomers, namely, *meso*-*cis*, racemic-*cis*, *meso*-*trans*, and racemic-*trans*. They ruled out the existence of optically active isomers in the absence of an asymmetric synthesis. Accordingly, the two isomeric acids, the higher melting isomer (141–143 °C from EtOH) and the lower melting isomer (125–127 °C from EtOAc), were misassigned as the *trans*- and *cis*-azo acids, respectively. This misassignment becomes extremely confusing because the putative existence of *meso* and enantiomorphous species was discarded, and the two isomers would simply be *cis*- and *trans*-diastereomers around the azo group. Certainly, (*E*)- and (*Z*)-configured isomers are plausible, albeit both *meso*- and *rac*-ACPA isomers should exhibit the most stable *trans*- or (*E*)-configuration from a thermodynamic standpoint (Figure 3). In fact, visible light switching of the azo group represents a paradigm that can be exploited in sensing and other applications.^12^

In the late 1990s, the development of initiators derived from ACPA for free-radical polymerizations once again faced the diastereomer problem. As surprising as it may be, a study by Gibson et al. ignored again the separation by Overberger and Labianca and retook instead the 1973 patent.^13^ They concluded rightly (in agreement with the 1970 antecedent)^7^ that the duplicated signals for the α-CH_3_ protons and methylene protons observed in the ^1^H NMR spectrum arise from the *meso-* and *d*,*l*-isomers. If had the azo compound been a mixture of *E*- and *Z*-isomers, more than two signals would have resulted in every case due to both geometrical and stereogenic isomerisms. However, these authors assigned the *d*,*l*-isomer with the higher melting point and the other as the *meso*-isomer. This argument was based on the direct comparison with the melting point and solubilities of tartaric acids, with *d*,*l*-tartaric acid having a higher melting point (206 °C) than *meso*-tartaric acid (140 °C). Clearly, this conclusion contradicts previous assignments. It seems, however, that Gibson et al. overlooked the distinctive nature of tartrate racemates; tartaric acid crystallizes as a racemic compound, not as a racemic mixture (conglomerate), and only some salts (like Pasteur’s sodium ammonium tartrate tetrahydrate) can crystallize either as racemic compounds or as conglomerates. It is true that *meso* and racemates do not bear any expected trend in physical properties given their diastereomeric relationship, while for a conglomerate system, the racemate always melts at a temperature below the corresponding enantiomers or scalemic mixtures derived thereof.

In view of our current knowledge of solid-state chirality,^14,15^ all of the experimental observations on ACPA, despite a complex and puzzling structural story, point to a stable homochiral aggregation that would account for a spontaneous separation of enantiomorphous crystals. As a useful radical initiator of polymerization in aqueous media^16^ and a component of photopolymers,^17^ the crystalline phases of ACPA (used as an unspecified mixture of stereoisomers) should unambiguously be characterized. This represents likewise an invitation to interrogate the origin of homochiral sequences in solid structures.

## Experimental Section

### Materials

Analytical-grade solvents and distilled water were used for preparing the corresponding ACPA solutions. All reagents were obtained from commercial suppliers and used without further purification. 4,4′-Azobis(4-cyanopentanoic acid) (ACPA, ≥98.0%, mp 118–125 °C) was purchased from Sigma-Aldrich.

### Stereoisomer Separation and Resolution

*Meso*- and *rac*-ACPA were obtained according to the method described in the literature by Overberger and Labianca,^7^ although at a lower scale, starting from a suspension of ACPA (15.07 g, 53.8 mmol) in a 10% aqueous methanol solution (904.2 mL), which was vigorously stirred at room temperature for 24 h. The white solid was collected by filtration and dried under vacuum (7.14 g, 47.4%). The filtrate containing the *d*,*l*-racemate was kept at 4–5 °C for 12 h, and the resulting crystalline material was filtered, washed with cold water, and dried (3.47 g, 23%). Fractional resolution could be accomplished by reproducing the seminal observations.^7,8^ An Erlenmeyer flask containing the *d*,*l*-racemate (0.31 g, 1.11 mmol) suspended in water (9.5 mL) was coupled to a reflux condenser, and the mixture was heated at 55–60 °C under mechanical stirring. After complete dissolution, both heating and stirring were stopped, and the flask was allowed to cool slowly to an ambient temperature (∼28 °C in the present case). After 24 h, the resulting prismatic crystals were filtered and dried under vacuum (0.24 g, 77%). This material had an mp of 114–115 °C, [α]_D_ = −6.0 (*c* 5 × 10^–3^ g/mL, MeOH, *T* = 28 °C), which increased after recrystallization from water (mp = 123–124 °C, [α]_D_ = −40.4 (*c* 6 × 10^–3^ g/mL, MeOH, *T* = 30 °C)). Consistent with the spontaneous conglomerate resolution, sometimes a dextrorotatory sample was obtained with similar ees [α]_D_ = +6.3 (*c* 6 × 10^–3^ g/mL, MeOH, *T* = 30 °C) that increases after recrystallization, [α]_D_ = +35 (*c* 6 × 10^–3^ g/mL, MeOH, *T* = 28 °C). Literature data on the isolated impure (+)-enantiomer: +1,4, +8.5, and +40.8 after a third recrystallization (90% ee), with mp from 120 to 127 °C.^7^

### Melting Points

Data were obtained from either a Barnstead Electrothermal (IA9100 model) or a Stuart Scientific (SMP3 model) digital apparatus and are uncorrected (relative to external pressure). A heating rate of 1 °C/min was used.

### Polarimetry

Optical rotations were measured on a Perkin-Elmer 241 polarimeter at the standard sodium D-line wavelength (589 nm) as well as at Hg-lamp wavelengths (578, 546, and 436 nm). All measurements were conducted in either water or methanol at room temperature (in the range between 17 and 28 °C over time). Specific optical rotations, [α]_λ_, were calculated from the corresponding optical rotation values using the expression: [α]_λ_ = α/*c* × *l*, where α is the observed optical rotation (°), *l* is the path length of the optical cell (1 dm), and *c* is the concentration (g/mL).

### Optical Microscopy

Crystal specimens were examined with a Dino-Lite digital microscope equipped with a light-emitting diode (LED) lamp and a BL-ZW1 backlight pad to detect the polarized light emitted. Rotating the polarizer dial changes the degree of polarization. When the degree indicator on the BL-ZW1 is aligned with the magnification indicator on the digital microscope, 0 and 180° readings on the dial are equivalent to no polarization. The photographs were taken on a Mac computer using the DinoXcope software interface.

### FT-IR Spectroscopy

Infrared (IR) spectra were recorded on a Nicolet iS5 Fourier-transform infrared (FTIR) spectrophotometer (Thermo Scientific) using KBr (Merck, spectroscopic quality) pellets of finely ground crystals. The spectral range was 4000–500 cm^–1^ with a resolution set at 4 cm^–1^. All acquisition data and spectral images were obtained using the OMNIC program provided by the supplier.

### NMR Spectroscopy

Both proton and carbon NMR spectra were recorded on a Bruker Avance 300 spectrometer equipped with a Bruker Ultrashield 300 magnet (working at 300 and 75 MHz for ^1^H and ^13^C nuclei, respectively) and using hexadeuterated dimethyl sulfoxide (DMSO-*d*_6_) as a solvent. Chemical shifts (δ, ppm) are referred to as tetramethylsilane (Me_4_Si, TMS) as the internal standard (δ = 0.00 ppm). For ^13^C NMR spectra, the peak assignment was facilitated using distortionless enhancement by polarization transfer (DEPT) experiments. Experiments aimed at determining chiral discrimination between ACPA enantiomers were conducted with a chiral lanthanide shift reagent, europium tris[3-(heptafluoropropylhydroxymethylene)-(+)-camphorate], and Eu(hfc)_3_ in deuterated solvents such as DMSO-*d*_6_ and CD_3_OD. ^1^H NMR spectra were recorded for solutions containing enantioenriched ACPA (20 mg, [α]_D_ = −6.0) and increasing amounts of Eu(hfc)_3_ from substoichiometric to 2.1 ratios.

### Differential Scanning Calorimetry

The DSC experiments were conducted on a DSC 214 Polyma Netzsch instrument at a nitrogen gas flow of 100 mL/min. Samples weighing between 1.75 and 1.80 mg were heated from 25 to 200 °C. The initial sample was kept at 25 °C for 15 min and then heated to 200 °C at a rate of 2 °C/min. Melting temperatures were calculated from the DSC curves, with the maximum value of the peak taken as the melting point. The enthalpy of fusion was also estimated from the curve at a resolution of 0.1 μW and precision in the range between ±0.05 and ±0.2%. Heating/cooling cycles can be performed from 0.001 to 500 K/min using an intracooler IC40 system capable of operating between −40 and 600 °C.

### Single-Crystal X-ray Diffraction Analyses

In all cases, a suitable single clear colorless crystal was obtained by slow evaporation and then selected and mounted on a MITIGEN holder with silicon oil on a ROD, Synergy Custom systems, HyPix diffractometer. The crystal was kept at a steady *T* = 100(2) K during data collection (with an Oxford Cryosystems low-temperature device). The structure was solved with the SHELXT solution program^18^ using dual methods and by using OLEX2 1.5-α as the graphical interface.^19^ The model was refined with SHELXL using full-matrix least-squares minimization on *F*^2^.^20^ Data were measured using ω scans with Cu Kα radiation. The diffraction pattern was indexed, and the total number of runs and images were based on calculations using the program CrysAlis^PRO^.^21^ The unit cell was also refined with the aforementioned package along with data reduction, scaling, and absorption corrections.^21^ Empirical absorption correction using spherical harmonics was implemented in the SCALE3 ABSPACK scaling algorithm. All nonhydrogen atoms were refined anisotropically. Hydrogen atom positions were calculated geometrically and refined using the riding model.

### Powder X-ray Diffraction

The PXRD patterns were collected on a Rigaku SmartLab diffractometer equipped with an in-plane arm, a 9 kW (45 kV, 200 mA) Cu target rotating anode generator, and a HyPix 3000 semiconductor detector. CBO optics in the Bragg–Brentano configuration were employed with primary and secondary axial Soller slits (5.0°), *R_x_*–*R_y_* stage, detector mode = 1D, diffraction angle (2θ) from 5 to 50° with a step size of 0.02, and a scanning rate of 5°/min at room temperature.

### Computational Details

In order to first prepare the input structures for the metadynamic (MTD) simulations of the molecular aggregates, the geometry adopted by ACPA molecules (both *RR* and *RS* stereoisomers) in the resolved crystal structures was employed as the starting point. These geometries were optimized at the GFN2-xTB level of theory^22^ in water with the ALPB continuum solvation model.^23^ The characterization of the optimized structures was evaluated by Hessian calculations showing zero imaginary frequencies. A conformational analysis of the *RR* and *RS* stereoisomers was carried out within the iMTD-GC workflow^24^ implemented in the conformer–rotamer ensemble sampling tool (CREST) program.^24^ This state-of-the-art methodology combines MTD simulations with atomic root-mean-square deviation (RMSD) for generating the conformers. We used the default settings of the iMTD-GC workflow,^24^ thus obtaining 517 conformers in an energy window of 6 kcal/mol at 400 K for both *RR* and *SS* isomers. This conformer search was carried out in water, with the GBSA implicit solvation model.^25^ The most stable conformer was optimized with the GFN-FF force field^26^ in water (ALPB). Then, that structure was used for building the molecular clusters by employing the automated interaction site screening (aISS) submodule of the xTB program package.^27^ The screening is based on the rigid intermolecular force field xTB-IFF^28^ followed by final optimization at the GFN-FF force field in water (ALPB). The methodology is as follows: an aISS calculation between two *RR* molecules was carried out, and then, the resulting dimer was optimized at the GFN-FF force field in water (ALPB). That dimer was used as an input for computing another aISS calculation in combination with the other *RR* monomer, thereby obtaining a trimer that was optimized at the above-mentioned level of theory. Again, the trimer was employed in a subsequent aISS calculation with another *RR* monomer. This protocol was carried out iteratively until reaching a cluster of 20 molecules, with a cluster containing 720 atoms. For each aISS calculation, a pocket searching was accomplished. This molecular cluster was then optimized at the GFN1-xTB^29^ level of theory in water (ALPB). This process was also carried out for the *RS* stereoisomer, with the aim of studying the interaction of *RR* and *RS* molecular aggregates. To build the clusters representing racemic and diastereomeric mixtures, the same methodology as above was employed, yet alternating the aISS calculation with the *RR* isomer and its mirror image *SS* isomer up to a 20-molecule cluster. This process was also carried out for the *RR* and *RS* stereoisomers. Thus, we built four clusters of 20 molecules each (720 atoms) that will hereafter be called ***RR*****–*****RR***, ***RS*****–*****RS***, ***RR*****–*****SS***, and ***RR*****–*****RS***. From these four optimized clusters, we ran MTD simulations up to 100 ps with the GFN1-xTB^29^ method in water (ALPB) at 298 K. The collective variables in the MTD simulation are based on the RMSD, taking as reference the input structure. Therefore, to describe the biasing potential, a scaling factor of the RMSD criteria (*k*_*i*_/*N*, where *N* is the number of atoms) was set to 0.02 *E*_h_, whereas the width of the Gaussian potential (α) was set to 1.2 Bohr^–1^. Since we are interested in the interactions of the whole molecular cluster, to avoid any molecular dissociation by disrupting the noncovalent interactions, such clusters were encapsulated in an ellipsoid of 67, 66, and 65 Bohr described by a logfermi potential that is set at the origin. Accordingly, to maintain all of the molecules inside the cavity, the center of mass of the cluster was positioned at the origin. Single-point Hessian (SPH)^30^ calculations within the modified rigid rotor harmonic oscillator approximation (mRRHO) were used to compute the corresponding free energies (*G*) of the final nonequilibrium geometries of MTD simulations. To avoid imaginary frequencies, a maximum deviation of 0.5 Å in the RMSD was allowed.

## Results and Discussion

### Structural Characterization

ACPA is a white solid that can be synthesized through a safe Strecker-type variation involving hydrazine sulfate,^6^ thus avoiding the hazardous use of HCN. The broad melting point range of commercially available samples (as large as 115–128 °C) points to a structurally inhomogeneous substance, even if chemically consistent with a given formula. The synthesis is poorly diastereoselective, albeit the ratio between *meso-* (often prevalent) and *rac*-ACPA varies from batch to batch. This ratio can be estimated through NMR by the integration of diastereotopic methyl groups, which appear as close, though well-differentiated signals, at higher field. The diastereotopicity is obviously caused by the vicinal chiral center, which renders the proton signals as nonequivalent. We checked the ACPA sample as received (mp 118–125 °C) and recorded its ^1^H and ^13^C NMR spectra in DMSO-*d*_6_ at an ambient temperature. After isomer separation, the *d*,*l*-pair exhibits consistently δ_CH_3__ ∼1.65 ppm, while the *meso-*isomer resonates downfield (δ_CH_3__ ∼1.69 ppm), from which a 53:47 *meso*/*d*,*l* ratio can be inferred (Figure S1). The methylene groups likewise show diastereotopicity, but the eight protons appear as a complex multiplet (δ_CH_2__ = 2.42–2.19 ppm for the *d*,*l*-pair). Similar correlations can be obtained from ^13^C NMR spectra (Figures S2 and S3), showing δ_CH_3__ = 23.1 and 22.9 ppm for *meso-* and *d*,*l*-stereoisomers, respectively. As expected, the most deshielded signal at δ ∼ 12.4 ppm (*d*,*l*-isomer), partially interchanged with the solvent, identifies the acidic proton.

Solid-state infrared spectroscopy still represents one of the most reliable and expeditious techniques to assess the molecular signatures of racemates. For a conglomerate, the IR spectrum of a racemic mixture is virtually superposable on that of the pure enantiomers, whereas it will be different in the case of a racemic compound.^31^ Differences are often small and unraveled by comparing the fingerprint region. More striking differences should be expected between *meso* and racemic structures in view of different lattice symmetries and molecular arrangements (Figures S4 and S5).

Both diastereomers show similar absorption bands, consistent with a common framework that differs spatially around the stereogenic carbons. As expected, greater differences are observable in the fingerprint region, such as the intense stretching vibration of the C=O group around 1700 cm^–1^ accompanied by bending vibrations of the carboxylate group and the stretching band of the C–O bond between 1500 and 1250 cm^–1^. The characteristic stretching vibration of the cyano group can be observed at 2244 (*meso*) and 2247 (racemate) cm^–1^. A different pattern for the less diagnostic broad bands above 2500 cm^–1^ is noticeable. Along with the stretching vibrations of the aliphatic C–H groups below 3000 cm^–1^, the more intense and poorly defined band corresponds to the stretching modes of the O–H groups. The marked difference in morphology could be attributed to the existence of different O–H-bonds and H-bonding in the solid structures, including solvent molecules. The *meso* derivative was purified by recrystallization from a 96% aqueous ethanol, while the *d*,*l*-isomer could be recrystallized from water. The broad stretching band spreading into 3500 cm^–1^ and beyond points to intermolecular H-bonding, which is consistent with the typical dimerization of carboxylic acids.^32^ For the racemate, the presence of two single peaks blue-shifted to 3524 and 3611 cm^–1^ could be attributed to strong H-bonds between water and monomeric carboxylic acid. In fact, the absorption at ∼1700 cm^–1^ (observed in the *d*,*l*-racemate as a doubling of the carbonyl stretching band at 1689 and 1717 cm^–1^) has often been ascribed to this feature.^33^ Clearly, the existence of occluded water molecules after drying cannot be ruled out. Hydrogen bonds weaken the C=O bond, resulting in absorption at a lower frequency than the monomer. The carbonyl absorption in dimerized saturated aliphatic acids appears between 1720 and 1706 cm^–1^.^32^ Moreover, in the specific case of carboxylic acid derivatives, racemates and pure enantiomers could even exhibit different arrangements of H-bonding and/or alkyl chains in the crystal structures,^31^ which translate into differences in band shifts and/or band splitting. While this can be true for racemic compounds, IR spectra of enantiomers are largely indistinguishable from that of a conglomeratic phase, irrespective of any scalemic (enantioenriched) composition (Figure S6).

Furthermore, we attempted to detect any potential chiral discrimination between ACPA enantiomers by NMR using chiral paramagnetic shift reagents, a well-established approach to quantify scalemic mixtures through the corresponding diastereomeric complexes.^34^ Peak broadening was a serious limitation and, although some separation could be observed for the methylene multiplets after the increasing addition of chiral Eu(hfc)_3_ to the sample beyond the stoichiometric ratio, the diagnostic methyl protons remained unaffected in different deuterated solvents (Figures S7–S9).

### Separation by Triage and Crystal Structure Analyses

On the basis of a conglomerate arrangement for ACPA, crystallization of the racemate under equilibrium conditions (*i.e.*, slowly, either with cooling or by evaporation) should lead to a racemic system from which it may be feasible to separate homochiral specimens of each handedness, which represents a classic Pasteurian resolution of the first kind.^35^ Unlike the early observation of spontaneous resolution by slow cooling of hot solutions, our initial screening was rather disappointing because in most cases a precipitate of tiny crystals was obtained, globally lacking optical activity (Figure S10). Certainly, such experiments were conducted on cooler days (below 20 °C) than those reported for the first fractional crystallization exceeding 35 °C.^8^ The fact that tweezers were used to separate platelets (enantioenriched dextro-enantiomer) from a powdery solid (the purified racemate) is intriguing because it is not immediately obvious if the latter still corresponds to a mechanical mixture of ACPA enantiomers. However, the latter is consistent with our observation of powdery solid formation without optical activity. Conversely, we were able to reproduce the spontaneous enantiomer disproportionation a few months later, when the hot solution was cooled to room temperature on warmer days (28–30 °C). Low yields of large prismatic crystals were obtained (Figure S10). This material exhibited arbitrary polarization, sometimes dextrorotatory, sometimes levorotatory, which is typical of spontaneous resolution.^35^ Although crystal diffraction should solve at last the solid-state structure, other innovative nonlinear spectroscopy methods, such as second harmonic generation (SHG), have recently been applied to spot conglomerates and imaging of anisotropic substances.^36,37^

We surmised that, while enantiomer resolution is a genuine phenomenon accounting for the conglomeratic nature of the *d*,*l*-racemate, the oft-encountered low optical purity could be linked to some twinning of opposite homochiral crystals. In fact, it was difficult to find single crystals suitable for diffractometry analysis because most of the crystals stick to each other (S11). Under polarizing light, the observation of interference colors in a clockwise or counterclockwise mode of crossed polarizers points to mixed aggregates of homochiral prisms. Conversely, fine needles were obtained for the *meso*-stereoisomer (Figure S12). Analyses of 10 randomly picked crystals obtained from the aqueous crystallization revealed that they had all spontaneously resolved into the conglomerate, without evidence of any other crystal form. This strategy enabled both (*RR*)- and (*SS*)-enantiomers to be successfully characterized. Results collected in Table 1 evidence the prevalence of the (*S*,*S*)-enantiomer. Obviously, such data are not statistically significant given the limited number of cases examined, although it is entirely possible that there is some bias introduced during crystal picking if one enantiomer forms crystals more suitable for X-ray diffraction. As detailed through the crystallographic discussion (vide infra), the absolute configurations of both enantiomers could be established unambiguously. Table 2 summarizes the key crystal structure parameters of both enantiomers and those of *meso*-ACPA (see the Supporting Information).

**Table 1:** Single-Crystal Determination of ACPA Enantiomers Randomly Chosen

sample	space group	Flack parameter	chirality	*R*_1_	*a* (Å)	*c* (Å)	*V* (Å^3^)
1	*P*4_3_2_1_2	0.05(3)	*S*,*S*	2.28	9.7532(1)	30.5485(3)	2905.92(7)
2	*P*4_3_2_1_2	–0.1(3)	*S*,*S*	2.32	9.7542(1)	30.5500(3)	2906.66(5)
3	*P*4_3_2_1_2	0.04(2)	*S*,*S*	2.29	9.7539(1)	30.5401(3)	2905.54(5)
4	*P*4_3_2_1_2	0.03(4)	*S*,*S*	2.24	9.75545(7)	30.5538(3)	2907.77(4)
5	*P*4_1_2_1_2	0.08(2)	*R*,*R*	2.31	9.7559(1)	30.5528(4)	2907.94(6)
6	*P*4_3_2_1_2	0.01(4)	*S*,*S*	2.39	9.75111(8)	30.5538(4)	2905.18(5)
7	*P*4_3_2_1_2	0.03(4)	*S*,*S*	2.34	9.7545(1)	30.5413(4)	2906.01(6)
8	*P*4_3_2_1_2	–0.04(3)	*S*,*S*	2.27	9.75266(8)	30.5495(4)	2905.70(5)
9	*P*4_1_2_1_2	–0.03	*R*,*R*	2.61	9.75002(9)	30.5420(5)	2903.41(6)
10	*P*4_3_2_1_2	0.09(4)	*S*,*S*	2.39	9.75635(7)	30.05399(3)	2906.98(4)

**Table 2:** Crystallographic and Structure Refinement Data for ACPA Enantiomers and *Meso* Compound

parameter	(*R*,*R*)-ACPA	(*S*,*S*)-ACPA	*meso*-ACPA
formula	C_12_H_16_N_4_O_4_	C_12_H_16_N_4_O_4_	C_12_H_16_N_4_O_4_
*D*_calc_ (g/cm^3^)	1.280	1.281	1.340
μ (mm^–1^)	0.825	0.825	0.863
formula weight	280.29	280.29	280.29
size (mm^3^)	0.30 × 0.12 × 0.12	0.14 × 0.10 × 0.08	0.15 × 0.03 × 0.02
*T* (K)	100(2)	100(2)	100(2)
crystal system	tetragonal	tetragonal	monoclinic
Flack parameter	0.04(4)	0.02(3)	
Hooft parameter	0.08(2)	0.05(3)	
space group	*P*4_1_2_1_2	*P*4_3_2_1_2	*P*2_1_/*n*
*a* (Å)	9.75590(10)	9.75320(10)	6.2063(2)
*b* (Å)	9.75590(10)	9.75320(10)	9.4255(3)
*c* (Å)	30.5528(4)	30.5485(3)	11.8841(4)
α (°)	90	90	90
β (°)	90	90	92.453(3)
γ (°)	90	90	90
*V* (Å^3^)	2907.94(7)	2905.92(7)	694.55(4)
*Z*	8	8	2
*Z*′	1	1	0.5
wavelength (Å)	1.54184	1.54184	1.54184
radiation type	Cu Kα	Cu Kα	Cu Kα
Θ_min_ (°)	4.758	4.760	5.994
Θ_max_ (°)	75.981	76.387	71.699
measured refls.	28,698	28,304	8132
independent refls.	2924	2959	1338
refls. *I* ≥ 2σ(*I*)	2911	2932	1226
*R*_int_	0.0199	0.0232	0.0342
parameters	185	191	96
largest peak	0.145	0.149	0.231
deepest hole	–0.152	–0.136	–0.187
GooF	1.096	1.097	1.039
*w*R**_2_ (all data)	0.0582	0.0579	0.0819
*w*R**_2_	0.0581	0.0577	0.0797
*R*_1_ (all data)	0.0232	0.0231	0.0366
*R*_1_	0.0231	0.0228	0.0329

ACPA enantiomers crystallize in a chiral type of the tetragonal system whose correct configurational assignment was determined by the low value (0.04) of the Flack parameter.^38,39^ Determination of the absolute structure using Bayesian statistics on Bijvoet differences resulted in a similar Hooft parameter.^40^ Although a chiral structure can occur in any of the 65 Sohncke space groups, the ACPA enantiomers crystallize within the 22 chiral space groups forming enantiomorphous pairs, *P*4_1_2_1_2 and *P*4_3_2_1_2, characterized by screw axes of opposite handedness. Notably, the occurrence of tetragonal space groups among chiral substances is scarce, mainly dominated by *P*2_1_ (monoclinic) and *P*2_1_2_1_2_1_ (orthorhombic) Sohncke groups, neither being inherently chiral space groups.^41,42^ The positive effect of one or more binary screw axes to accommodate pure enantiomers has been related to an optimal close-packing favored by electrostatic interactions. In fact, the binary axis along with glide planes also occurs in the most frequently encountered groups for racemic crystals, mostly belonging to the *P*2_1_/*c* systems.^43^

**Figure 4:**
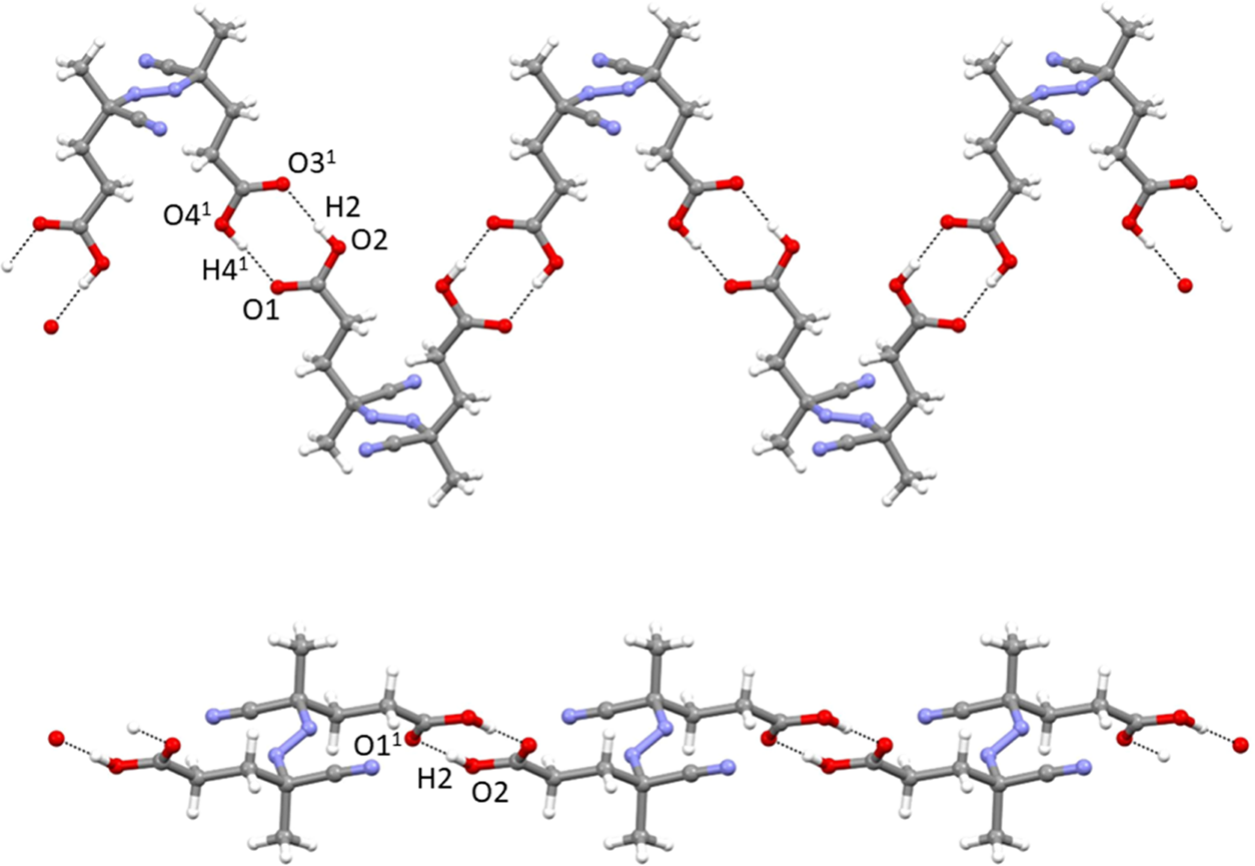
Hydrogen-bonding patterns in the ACPA chain for (top) homochiral molecules along the [1–10] direction (2_1_ screw axis) and (bottom) *meso* compound along (approx.) the [104] direction.

In ACPA enantiomers, the helicoidal arrangement around the screw axis evidences the otherwise expected H-bonding association between adjacent carboxylate groups, which manifests itself in the *meso* derivative as well, the latter lacking the helical twist (Figure 4 and Table 3). This dipole–dipole contact represents the main stabilizing motif in the crystal lattice, albeit other noncovalent interactions involving the parallel chains should also be present in the solid structure and in solution (see the Theoretical Analysis: Homochiral *vs* Heterochiral Interactions Section). However, H-bond distances between donor–acceptor termini do not indicate a greater stability of homochiral dimers relative to the symmetrical diastereomer. Regarding the helicity in chiral space groups, a comparison of chiral structures built on helical arrangements indicates that handedness most likely stems from 3_1_/3_2_ or 6_1_/6_5_ units present in hexagonal arrangements. In tetragonal packings (the case of ACPA enantiomers), 4*_n_* helices can either crystallize in chiral or achiral structures,^44^ which might likewise be related to the ability to obtain resolvable species crystallizing with noncentrosymmetric packing.

**Table 3:** Hydrogen Bond Information for ACPA Stereoisomers

(*S*,*S*)-enantiomer						
D	H	A	*d*(D–H) (Å)	*d*(H–A) (Å)	*d*(D–A) (Å)	D–H–A (deg)
O2	H2	O3^a^	0.92(3)	1.73(3)	2.6382(14)	172(2)
O4^1^	H4^1^	O1^b^	0.86(3)	1.80(3)	2.6521(13)	172(2)

(*R*,*R*)-enantiomer						
D	H	A	*d*(D–H) (Å)	*d*(H–A) (Å)	*d*(D–A) (Å)	D–H–A (deg)
O2	H2	O3^c^	0.84	1.82	2.6541(13)	173.1
O4^1^	H4^1^	O1^d^	0.84	1.81	2.6406(14)	172.7

*meso* compound						
D	H	A	*d*(D–H) (Å)	*d*(H–A) (Å)	*d*(D–A) (Å)	D–H–A (deg)
O2	H2	O1^e^	0.94(2)	1.73(2)	2.6736(12)	178.9(19)

a (−*y*, 1 – *x*, 1/2 – *z*).

b (1 – *y*, −*x*, 1/2 – *z*).

c (+*y*, −1 + *x*, 1 – *z*).

d (1 + *y*, +*x*, 1 – *z*).

e (−*x*, 1 – *y*, −*z*).

**Figure 5:**
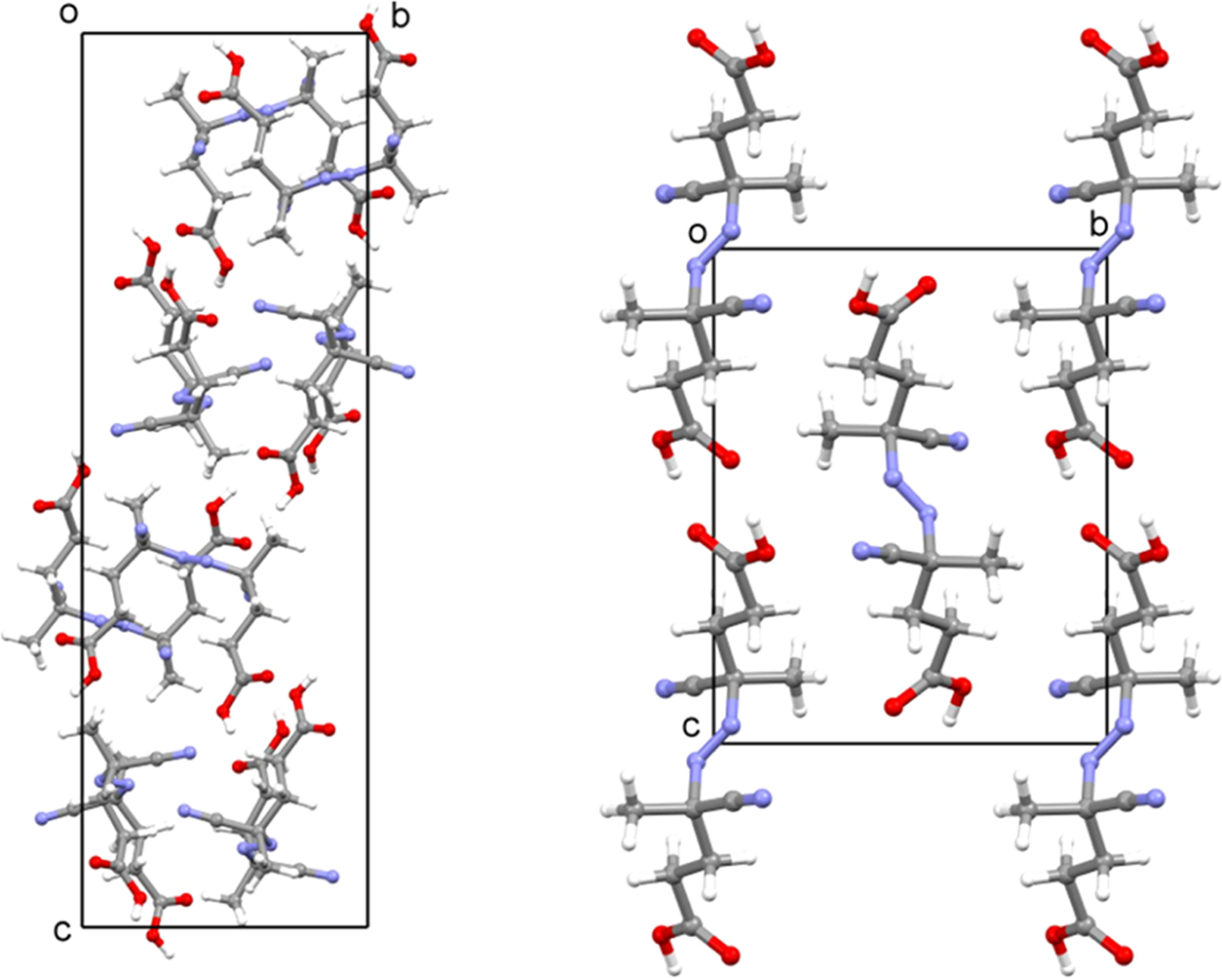
Representation of the molecular packing of ACPA molecules in the unit cells of a single enantiomer (left) and *meso* compound (right); diffraction data collected at 100 K.

It is worth noticing the molecular packing of ACPA stereoisomers in the unit cells (Figure 5), which were determined by diffraction analyses and determination of *Z* and *Z*′ values. The former denotes the number of molecules (or formula units) in the whole unit cell, while the latter accounts for the number of symmetry-independent molecules in a crystal structure.

The coexistence of multiple structurally distinct molecules in the asymmetric unit (*Z*′ > 1) is not unusual and constitutes a plausible solution to the problem of packing molecules in a 3D Euclidean space, especially for cocrystals and polymorphs.^45^ However, high *Z*′ values in enantiomerically pure species and racemates are often an oddity,^45−47^ with the exception of kryptoracemates.^48^ Both (*R*,*R*)- and (*S*,*S*)-configured ACPA enantiomers show *Z* = 8 and *Z*′ = 1, and hence there is a single symmetrically independent molecule in the asymmetric unit, thereby agreeing with a homochiral structure without significant disorder or stacking at the supramolecular level, which are encountered in higher *Z*′ crystal structures. For *meso*-ACPA, *Z* = 2, and *Z*′ = 0.5 (Figure 5), only half of the formula unit is present in the asymmetric unit, with the other half consisting of symmetry equivalent atoms. Interestingly, the *meso*-isomer is significantly denser than their chiral counterparts (1.340 *vs* 1.280 g/cm^3^), a fact also observed in tartaric acids for which the *meso* and racemic compounds are slightly denser than the enantiomer. Attempts to correlate *Z*′ values with the strength of molecular interactions through physical properties like density have led to inconclusive statements.^45,49^ Differences in density may be ascribed to the number and directionality of H-bonding and contribute to more tightly packed in the heterochiral association, like that of racemic compounds. The absence of an observable racemic compound phase in ACPA hampers the ability to verify further this assumption. While the classical Wallach’s rule stating that racemic crystals tend to be denser than their enantiomers is no longer true on average;^50^ the stabilizing advantage (from a thermodynamic standpoint only) of racemic crystal structures has been attributed to the formation of centrosymmetric dimers, whereas homochiral crystals are held together by weaker screw-symmetric ribbons.^51^*Meso*-isomers, however, represent borderline cases exhibiting either higher or lower density than other stereoisomers, with some reliable trends for polar molecules.^52^ This thermodynamic point aided by computation will be discussed in more detail (vide infra).

**Figure 6:**
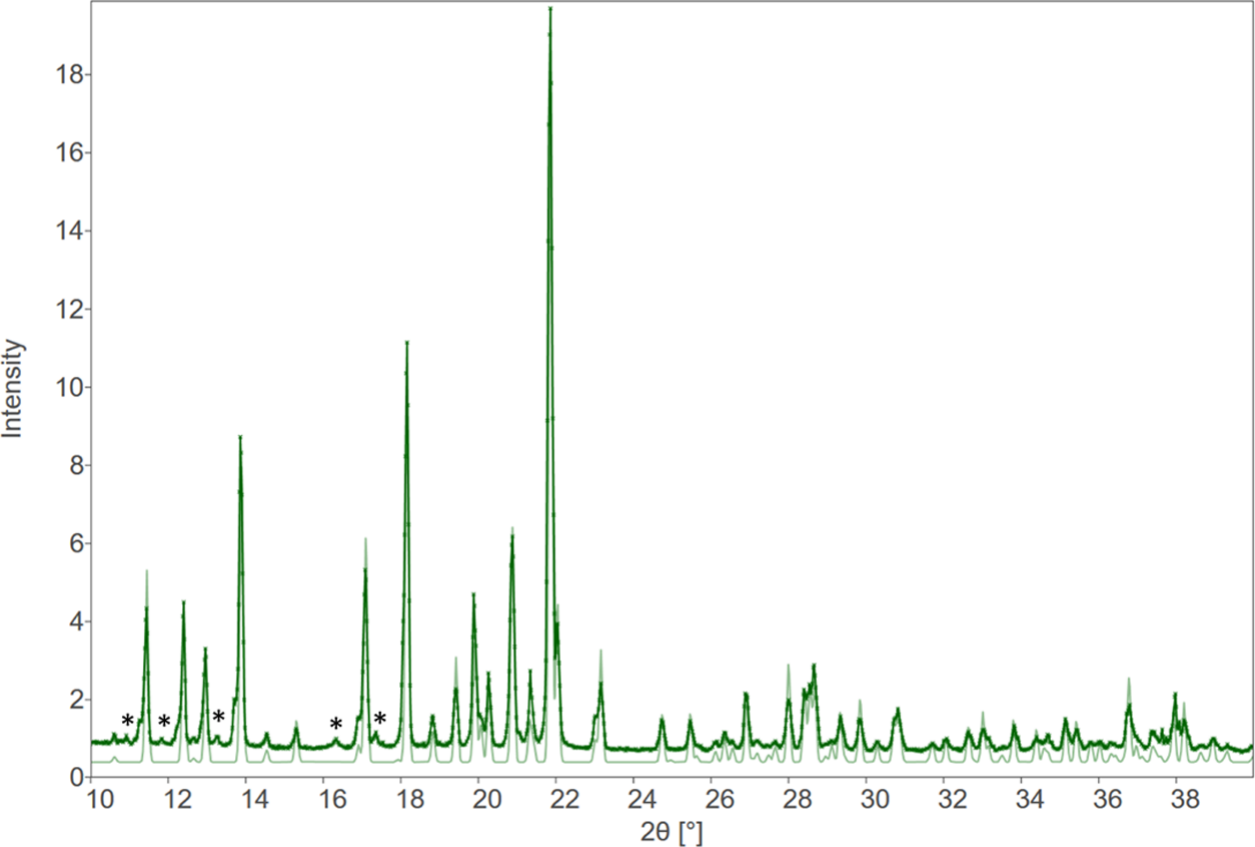
Experimental PXRD pattern (dark green) and calculated pattern from single-crystal structure, SXD (light green) of ACPA-racemate. 100 K-SXD cell parameters are normalized to RT values as refined against PXD data: *a* = 9.8903(13), *c* = 30.899(4). Peaks marked with an asterisk (*) are from tungsten contamination of the rotating anode.

**Figure 7:**
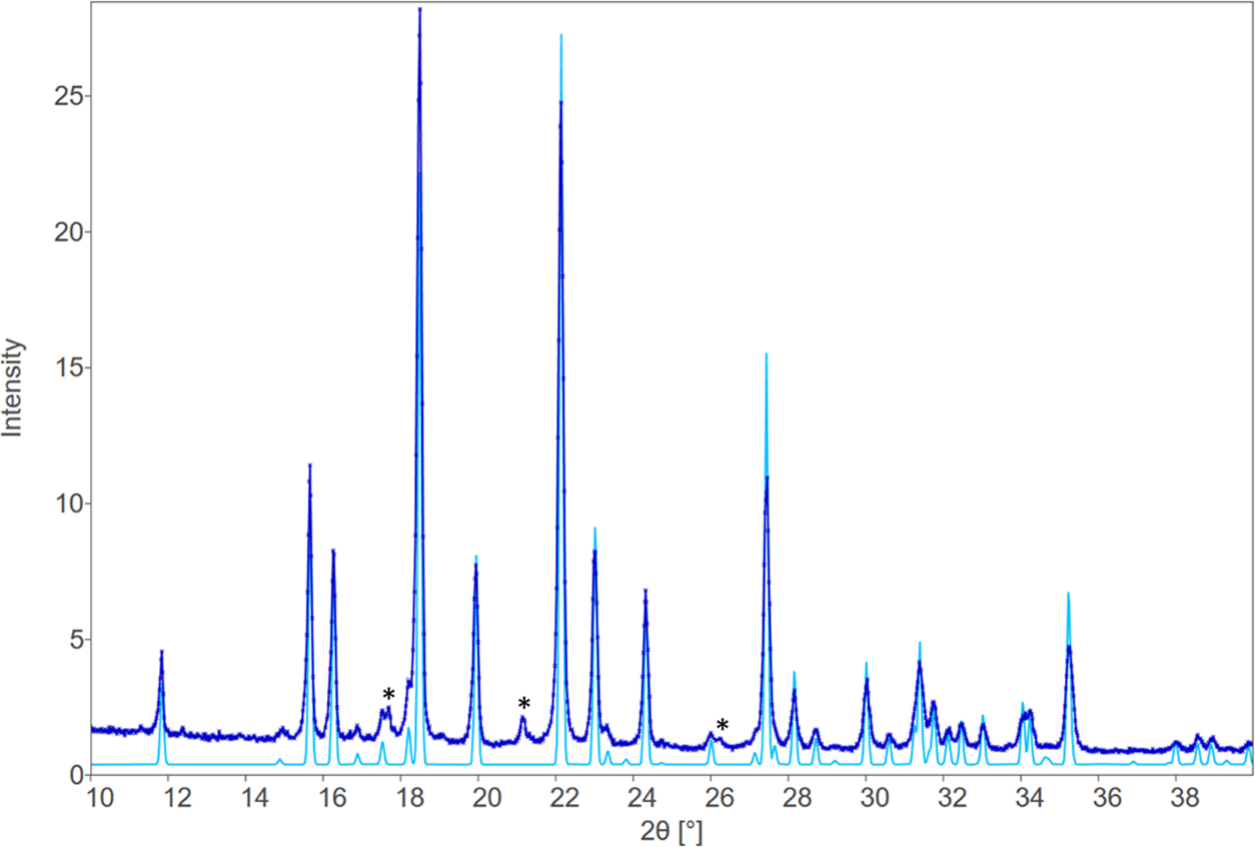
Experimental PXRD pattern (dark blue) and calculated pattern from single-crystal structure SXD (light blue) of *meso*-ACPA. 100 K-SXD cell parameters are normalized to RT values, as refined against PXRD data: *a* = 6.2724(12), *b* = 9.5881(17), *c* = 11.910(2), β = 92.634(3). Peaks marked with an asterisk (*) are from tungsten contamination of the rotating anode.

PXRD data show the characteristic diffraction peaks of crystalline materials with patterns matching the unit cell parameters of ACPA stereoisomers obtained by single-crystal X-ray diffraction (Figures 6 and 7). The distinctive peaks of the pattern for *meso*-ACPA were observed at 2θ = 11.8, 15.7, 16.3, 18.5, 19.9, 22.1, 23.0, 24.3, and 27.5°, whereas the *d*,*l*-racemate showed characteristic signals at 2θ = 11.4, 12.4, 13.9, 17.1, 18.2, 20.9, 21.9, 22.0, and 23.2°. Consistency can be illustrated through the overlay of powder patterns with refined unit cells (SCXD) from PXRD, *i.e.*, what the room-temperature unit cell would be. Scalamates are superimposable with the racemate, thus confirming anew its conglomerate nature as both enantiomers exhibit identical PXRD patterns.

### Thermal Analyses

**Figure 8:**
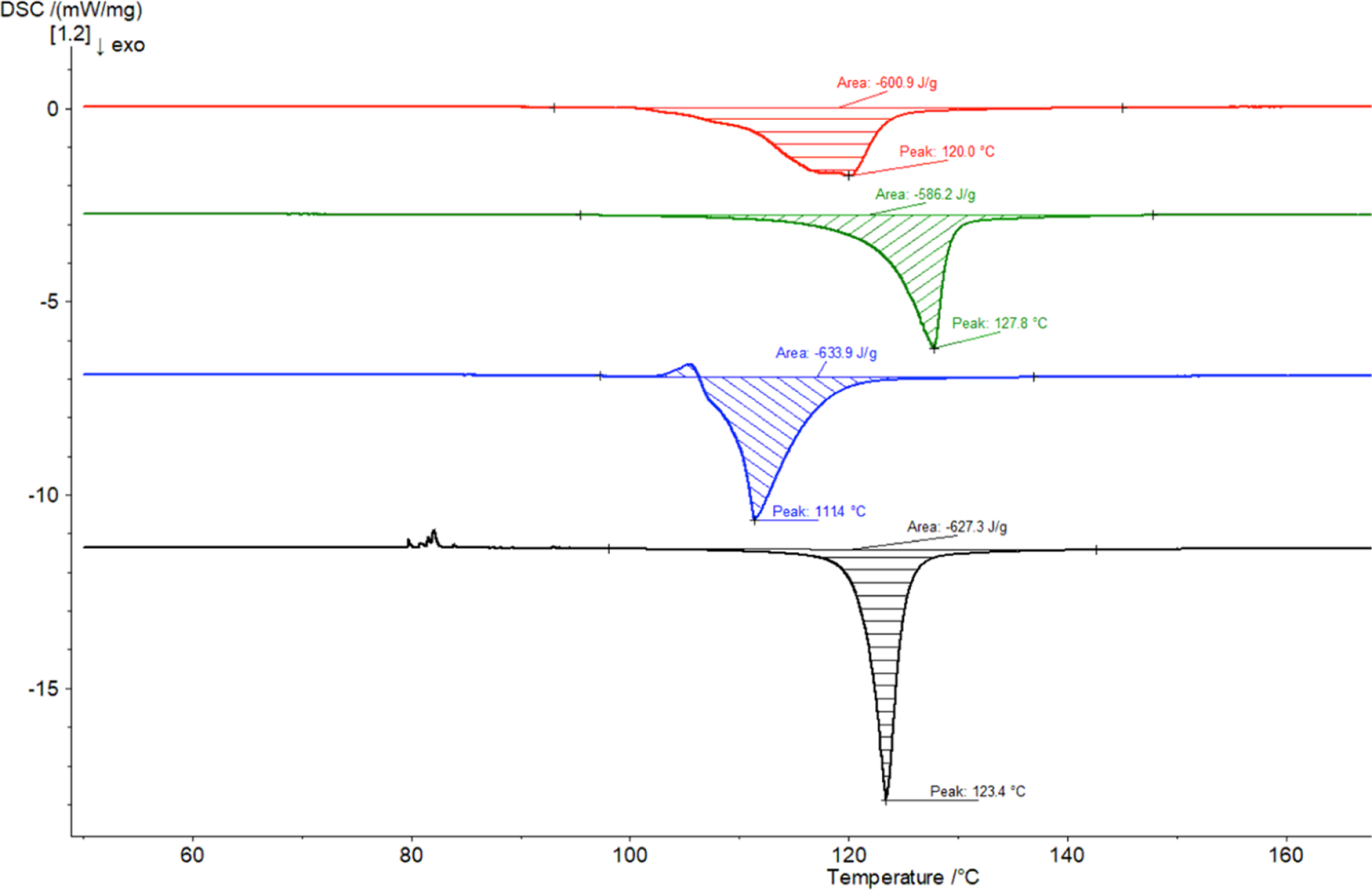
DSC plots obtained for ACPA (top, commercial sample) and its stereoisomers (in decreasing order): *meso* compound, *d*,*l*-racemate, and enantioenriched scalemic sample. Curves were recorded between 0 and 200 °C (shown until 160 °C for clarity).

As noted earlier, the melting point of ACPA stereoisomers has been a matter of confusion. Melting ranges most likely reflect a compositional variation of *meso* and racemic isomers, although there is another key point that should be kept in mind. Like other azonitriles (and hence their utility as radical initiators), ACPA decomposes thermally and releases nitrogen, at least from 70 °C in.^6^ We observed some browning above *ca.* 90 °C and before melting. Definitively, the melting point is not very reliable to estimate stereoisomer integrity. Differential scanning calorimetry (DSC) curves were obtained, however, to compare the thermal stability of diastereomers and verify that a scalemic mixture melts above the eutectic (conglomerate) composition (Figure 8). DSC measurements were recorded on ground crystals to give accurate values of the melting temperature and the enthalpy of fusion (Table 4). The melting temperatures of such mixtures showed good repeatability and matched well with the melting intervals measured with a digital apparatus, despite sample browning. It is customary to establish a straightforward relationship between Δ*H*_f_ and *T*_m_ values with the thermodynamic stability, which frequently holds for racemic compounds with respect to their enantiomers, also agreeing with the higher values calculated for lattice energy and density. While, as mentioned above, this assumption can no longer be a rule of thumb, the higher melting temperature and higher density of the *meso* diastereomer would indicate enhanced stability of this heterochiral structure. Actually, *meso* and conglomeratic ACPA decompose at different rates in aqueous solution, albeit the rates are similar in organic solvents.^3^

**Table 4:** Melting Data and Enthalpies for Stereoisomeric ACPA Samples

sample	initial mass (mg)	Δ*H*_f_ (J/g)	*T*_onset_ (°C)	*T*_m_ (°C)
ACPA (commercial)	1.79	–670.8	107.0	120.0
*meso-*isomer	1.80	–606.9	121.5	127.8
*d*,*l*-racemate	1.75	–644.6	108.9	111.4
scalemic *d*,*l*	1.76	–705.0	121.8	123.4

The heat of fusion measured on DSC thermograms can be calculated to determine the composition between eutectics or above the eutectic for racemic compounds or conglomerates, respectively.^53^ This follows the well-known Prigogine–Defay and Schröder–van Laar equations, so long as the melting temperatures of the racemate and pure enantiomers are known with accuracy and assuming ideal behavior.^54^ This is not the case with ACPA due to decomposition. For a conglomerate, the peak area of the racemate is directly proportional to the heat to melt the racemate, and hence the composition of a scalemic sample could be inferred from the whole area. However, the enthalpies of the racemate and the enantiomer are unequal, even in a conglomeratic system.^54^ Within this limitation, a rough estimation of the scalemic sample (last entry, Table 4) gives rise to ∼8.5% *ee*, slightly higher than the polarimetric measurement in solution (the sample being enriched in the levorotatory enantiomer).

### Theoretical Analysis: Homochiral *vs* Heterochiral Interactions

The existence of a conglomerate phase represents invariably an opportunity to assess a repeating issue in crystal stereochemistry, namely, whether racemic compounds are more stable than homochiral aggregates, which date back to pioneering manuals^9,31^ without clear-cut conclusions. A dominant argument is that racemic compounds are thermodynamically stable phases given their abundance (∼90% of known chiral substances) over conglomerates, which are often defined as metastable or kinetic racemates. The occurrence of conglomerates could be much higher than expected; simply, their exploration has been scarce, and we would like to extrapolate the famous McCrone’s aphorism on the search of polymorphs^55^ to conglomerates, *i.e.*, a matter of time and money spent. In fact, a recent survey has identified over 1800 potential conglomerate-forming substances within the Cambridge Structural Database (CSD) beyond the biological chemical space.^56^ And (homo)chiral crystals could also amount to more than 20% among known structures, leaving aside that chiral minerals do also exist.^57^ Despite recent advances in crystal structure predictions,^58^ studies aimed at elucidating any differences between homochiral and racemic compound phases are conducted at the semiempirical level or using *ab initio*/DFT methods with restricted basis sets. In any event, there seems to be no clear thermodynamic advantage of racemic compounds in terms of energy differences. On the other hand, conglomerate crystals, like ACPA, survive under a broad range of experimental conditions, thus witnessing enhanced stability. For chiral carboxylic acids, crystal packing most likely determines the stability of stereoisomer structures, with racemic crystals forming centrosymmetric H-bonded dimers, whereas homochiral crystals tend to form H-bonded chains.^59^ A similar trend has been observed for some APIs, such as the adrenoblockers propranolol, pindolol, or prenaterol, in which the supramolecular assembly in crystals reveals distinctive arrangements, namely, infinite one-dimensional chains around helical axes for single enantiomers, while racemates are dominated by zero-dimensional cycles around inversion symmetry elements.^60^

In a similar way to stereoselective chemical reactions, where the formation of either kinetic or thermodynamic products applies, the homochiral-racemic dichotomy could only be solved by assessing a larger landscape of environmental conditions, especially temperature and pressure. Within these limitations, however, one should invoke the highly cited analysis by Gavezzotti and Rizzato focused on the origin of stability favoring either stereoisomer. Without reaching a definitive conclusion, they postulated that “at least for homogeneous nucleation, a probabilistic factor, from kinetics or from statistical predominance of mixed *versus* enantiopure aggregates, must be in action during the early separation of liquid-like particles, which are thought to be the precursors of crystal nucleation”.^61^ Thus, by taking this inspiration, we believe that factors accounting for liquid-phase aggregation should be present in the early stages of nucleation. Experimental nucleation events, however, lie in the seconds time scale, and even powerful supercomputers are unable to simulate hundreds of microseconds per day with atomic precision. This limitation can be overcome by recent developments to augmentate nucleation sampling.^62^ We chose, in line with this argument, state-of-the-art metadynamic (MTD) simulations that capture some picoseconds of molecular aggregation leading to discrete, yet significant, atomic clusters where clues on enantiomer organization could be imaged. To this end, the computational study focuses on the influence of structural elements present in mixtures of ACPA stereoisomers *versus* pure stereoisomer aggregates. A set of four clusters comprising 20 molecules each were generated and evaluated. One contained only the *RR* stereoisomer (***RR***–***RR***) and the other the *RS* (***RS***–***RS***), whereas the two remaining cases were modeled as equimolar mixtures of *RR* and *SS* (***RR***–***SS***) and *RR* with *RS* (***RR*****–*****RS***). Since ACPA shows numerous σ bonds that allow a high number of torsions, we undertook first a conformational study of the *RR* and *RS* stereoisomers for obtaining the most stable conformation in water (implicit solvation). The starting structures were those of isolated monomers whose geometries were determined by single-crystal X-ray diffractometry. The conformational analysis is based on MTD simulations carried out at 400 K for an energy window of 6 kcal/mol (see the Computational Details Section).

In addition, even if the *trans* azo linkage in ACPA stereoisomers is clearly shown by crystal structures, we wanted to corroborate further the stability of such an arrangement relative to the *cis* configuration and performed a complete conformational analysis on both (*E*)- and (*Z*)-isomers of *meso* and enantiopure ACPA. The thermochemical analysis at 298.15 K indicates that the (*E*,*R*,*R*) diastereomer is more stable than (*Z*,*R*,*R*) stereostructure by 5.9 kcal/mol. The energy difference is even larger (9.3 kcal/mol) between (*E*,*R*,*S*) and (*Z*,*R*,*S*) arrangements. Such data would reasonably rule out the existence of (*Z*)-isomers in appreciable amounts in solution.

**Figure 9:**
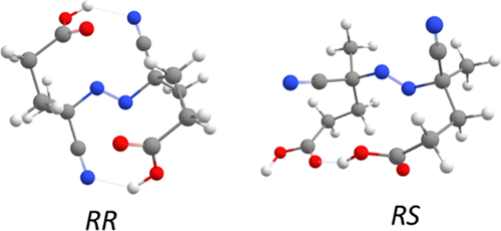
Optimized geometries for the most stable conformers of *RR* and *RS* ACPA stereoisomers.

Figure 9 shows the optimized geometries of the two more stable conformers for both *RR* and *RS* stereoisomers among 517 conformational arrangements.

**Figure 10:**
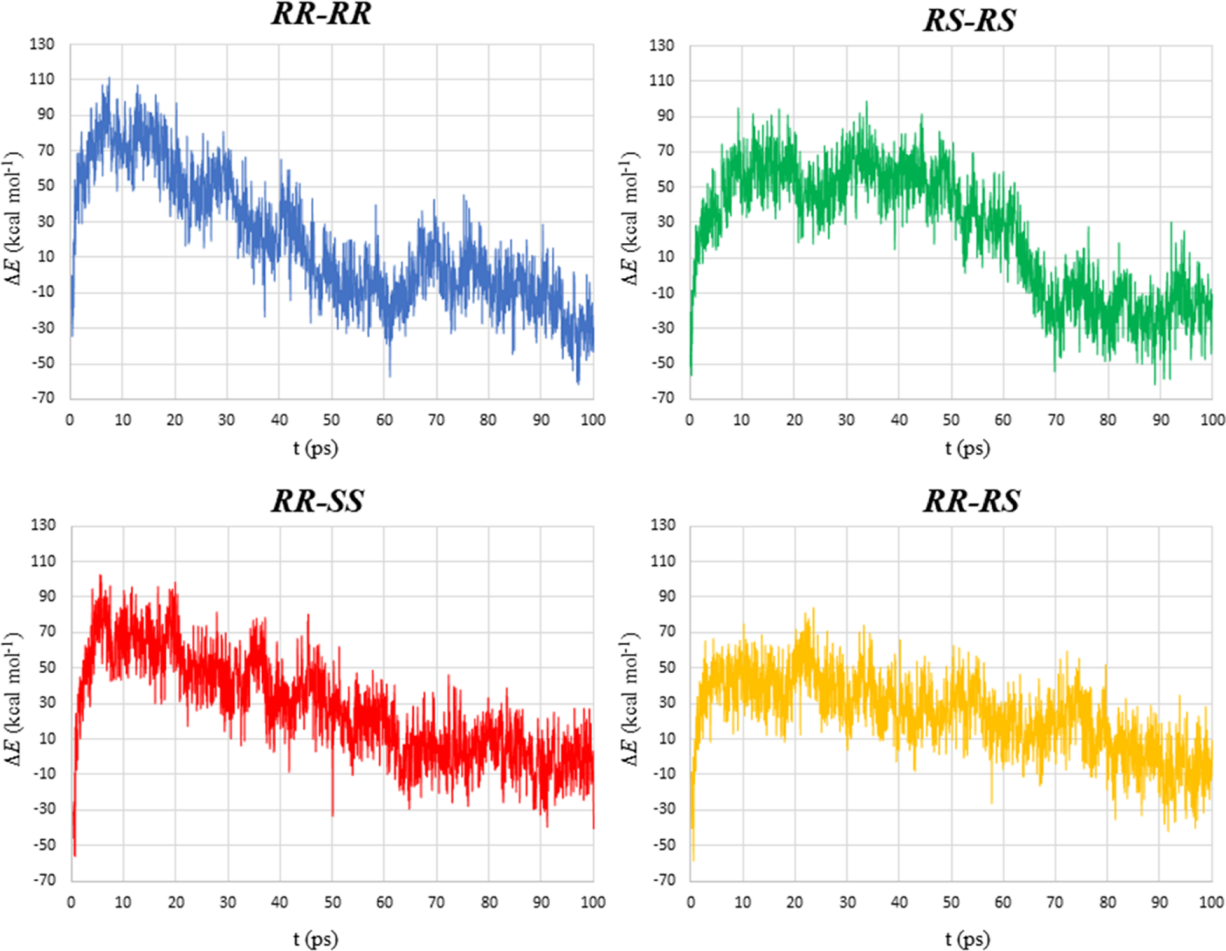
Relative energies along 100 ps of MTD/GFN1-xTB simulation in water (ALPB) at 298 K for the clusters ***RR***–***RR*** (blue), ***RS***–***RS*** (green), ***RR***–***SS*** (red), and ***RR***–***RS*** (yellow). All energy data are given relative to that of ***RR***–***RR*** at 0.25 ps.

Structures shown in Figure 9 were used to generate the four clusters studied by means of aISS calculations (see the Computational Details Section). Figure 10 also shows the relative energy of such clusters for 100 ps of the MTD simulation. The relative energy is given with respect to the *RR*–*RR* cluster at 0.25 ps, which is more suitable for comparative purposes, even though the optimized structure refers to 0.0 ps. As inferred from such plots, the aggregates arising from the same stereoisomer (***RR***–***RR*** and ***RS***–***RS***) show a maximum between ∼5 and 20 ps. Then, the energy decreases notably from 50 ps onwards in the case of ***RR***–***RR*** and from 70 ps in the case of ***RS***–***RS***-containing clusters. After reaching 100 ps, the energy of the ***RR***–***RR*** cluster decreases to about −30 kcal/mol, while the ***RS***–***RS*** one moves to nearly −20 kcal/mol. On the other hand, for the mixture of enantiomers (***RR***–***SS***), this variation in energy is less pronounced at the simulation onset and decreases more steeply than for the two previous clusters. For the ***RR*****–*****RS*** diastereomeric cluster, this energy change is even less pronounced than for the ***RR***–***SS*** counterpart and follows a straight-line trend with a low slope through the entire simulation. When the dynamics reaches 100 ps, the energies of the ***RR***–***SS*** and ***RR***–***RS*** clusters are higher than those of ***RR***–***RR*** and ***RS***–***RS***, being *ca*. −10 kcal/mol for both mixtures.

**Figure 11:**
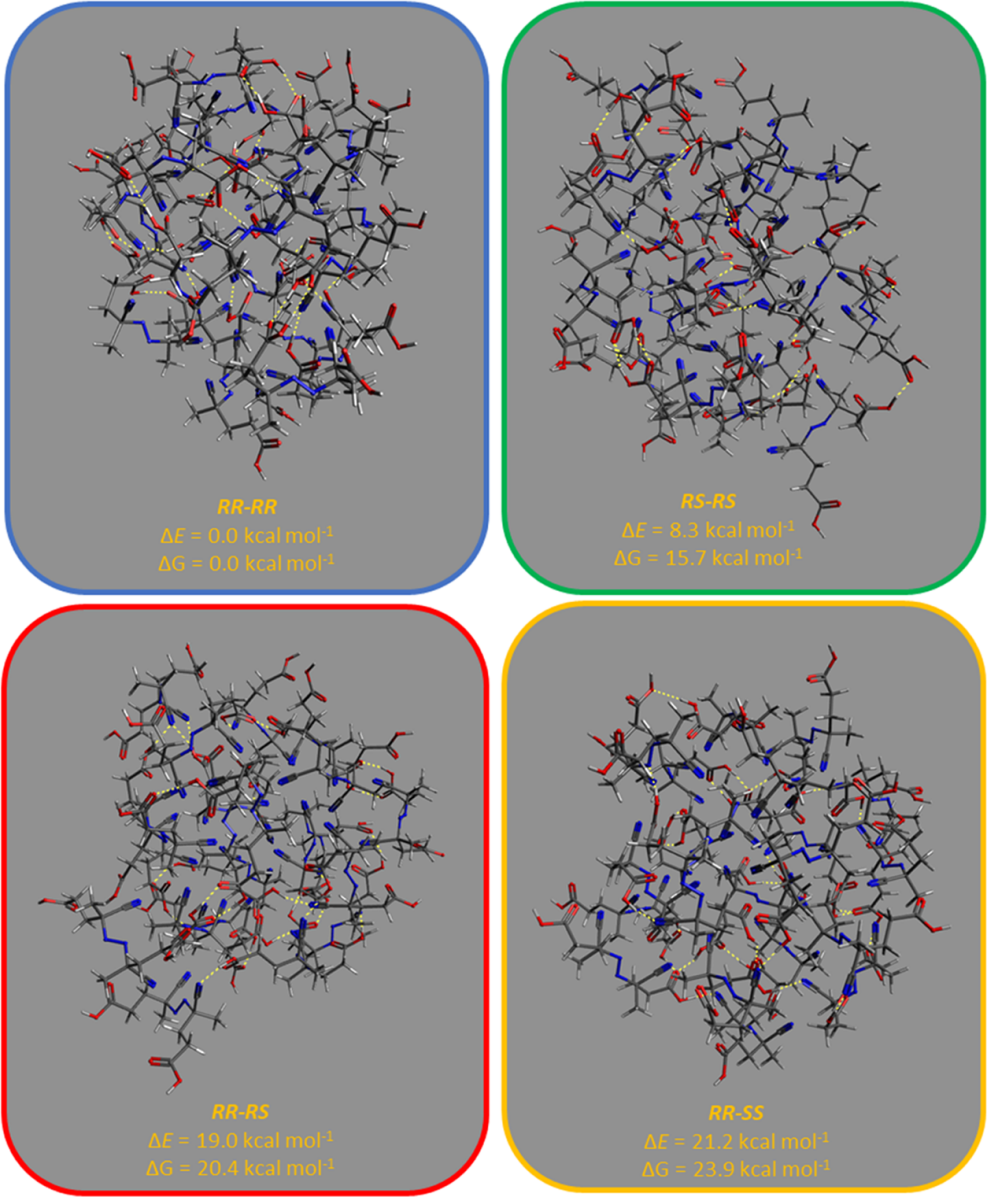
Optimized geometries at 100 ps of the MTD simulation at GFN1-xTB in water (ALPB), with a change in the RMSD of 0.5 Å with respect to the input structure. Relative energies are given with respect to the most stable (***RR***–***RR***) aggregate. Yellow dotted lines represent hydrogen bonds. The color code of the frames refers to simulations shown in Figure 10. Gray backgrounds are included to visualize hydrogen atoms (white).

Furthermore, we computed SPH for the final cluster geometries within the mRRHO approximation. The smooth optimizations prior to the Hessian calculation were performed for a change in the RMSD of 0.5 Å. The resulting structures are shown in Figure 11 together with the electronic and free energies computed at 298 K with respect to the most stable cluster, *i.e.*, the ***RR***–***RR*** aggregate. Such optimized geometries exhibit very similar structural features. The ***RR***–***RR***, ***RR***–***SS***, and ***RS***–***RS*** aggregates (Figure 11) have 14 out of 40 carboxylic groups in which the OH bond points away from the cluster, and 15 for the ***RR***–***RS*** aggregate. The density of the four clusters is quite similar as well, ranging between 0.040 and 0.042 atoms/Å^3^. Also, the number of hydrogen bonds are surprisingly identical, amounting to 25 in ***RR***–***RS***, ***RR***–***SS***, and ***RS***–***RS*** clusters and 26 for the ***RR***–***RR*** aggregate. In addition, the dipole moment shows no significant trend to justify energy differences associated with polarization; thus, values of 42.4, 58.0, 20.1, and 55.6 Debye were obtained for the ***RR***–***RR***, ***RS***–***SS***, ***RR***–***RS***, and ***RR***–***SS*** clusters, respectively.

**Figure 12:**
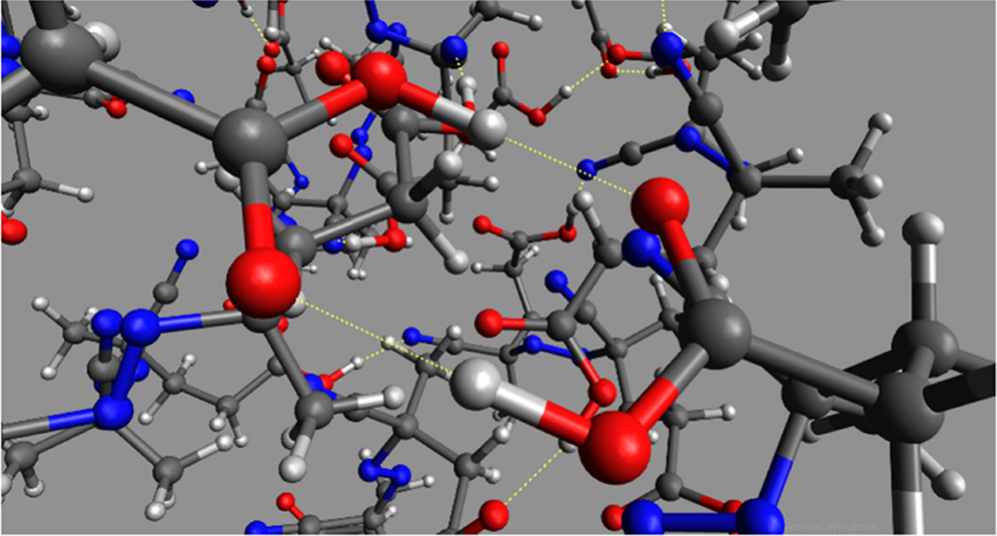
Dimer-like interaction between two carboxylic acids at the supramolecular level leading to the ***RR***–***RR***-configured cluster.

The main structural difference at the supramolecular level is, however, related to the type of H-bonding when aggregation takes place. Thus, the typical interaction involving carboxylic acid dimers has been found in all four cases, where the OH of the carboxylic group acts as a H-bond donor with the carbonyl oxygen of the other molecule (see Figure 12). Notably, these arrangements are more numerous in the pure stereoisomer aggregates, showing five of such interactions in both the ***RR***–***RR*** and ***RS***–***RS*** clusters. Conversely, the aggregates for which a solid-state structure could not be detected; namely, the racemic (***RR***–***SS***) and diastereomeric (***RR***–***RS***) aggregates, form exclusively two and three H-bonding interactions, respectively. This distinctive structural motif seems to be key for the stability of clusters in solution. In fact, these types of noncovalent interactions constitute the only H-bonds present in the crystal structures.

## Conclusions and Outlook

In conclusion, this study dissects for the first time the structural features of all diastereomers of ACPA, a valuable water-soluble azobis-nitrile dicarboxylic acid capable of initiating radical reactions and used as a stereoisomer mixture, which exhibits a tartaric acid-like behavior in view of its symmetrical arrangement. Crystallographic data are provided for the achiral isomer (monoclinic space group) and the two enantiomers, which crystallize in chiral Sohncke space groups (tetragonal *P*4_1_2_1_2 and *P*4_3_2_1_2 groups). While the spontaneous resolution of ACPA enantiomers by fractional crystallization was known, experimental results now available support unambiguously the occurrence of a stable conglomeratic racemate existing as separate homochiral domains. Levo- and dextrorotatory crystal excesses are arbitrarily obtained, consistent with the stochastic nature of Pasteurian resolutions.

The state-of-the-art MTD calculations show that all aggregates have, in general, similar behavior in solution. Remarkably, the most salient changes from both dynamic and structural viewpoints were found for supramolecular clusters that involve either ***RR***–***RR*** and ***RS***–***RS*** interactions, in line with the experimental results. Such aggregates exhibit more pronounced changes in energy at the beginning of the MTD simulation (5–20 ps) and then drop sharply to about −30 kcal/mol at 100 ps. On the other hand, the aggregates formed by racemic (***RR***–***SS***) and diastereomeric mixtures (***RR***–***RS***) show a smoother energy change along the trajectory, reaching *ca*. −10 kcal/mol at 100 ps of the MTD simulation. These energy variations are mainly associated with the number of dimer-like intermolecular interactions between the carboxylic acids, up to 5 in the homogeneous clusters involving ***RR***–***RR*** and ***RS***–***RS*** stereoisomers and 2 and 3 for ***RR***–***SS*** and ***RR***–***RS*** clusters, respectively.

It should be finally pointed out that ACPA molecules are of interest and offer further room for stereochemical studies. *Meso*-ACPA should undergo chemical desymmetrization,^63^ thus paving the way to other enantiomerically pure derivatives. Moreover, a solid-state conglomerate coupled with deracemization in solution should enable symmetry breaking through the Viedma ripening leading to complete enantiopurity.^64,65^ While this has proven to be successful with numerous compounds, most cases, if not all, involve stereogenic carbons adjacent to an acidic α-H that can easily be deprotonated with DBU (the usual base employed in organic solvents) to induce liquid-phase deracemization. The strategy can even be extended to conglomerates containing two chiral centers.^66^ However, ACPA has two quaternary chiral carbons, which should then undergo simultaneous racemization, not an easy task indeed. So long as deracemization can be achieved, mechanisms other than enolate formation would be required. To the best of our knowledge, there is only one example that reports Viedma deracemization of isoindolinones containing one quaternary chiral atom,^67^ for which Sakamoto and his associates unveiled that racemization should be involving ring opening to an achiral intermediate followed by ring closure.^67a^ A similar bond-breaking recombination pathway would hardly be plausible for ACPA. Summing up, these challenges are currently under investigation in our laboratories.

## Supporting Information Available

NMR and IR spectra, crystallographic data, and computational simulation results (PDF)
