# Supplementary material for: Revisiting Homochiral versus Heterochiral Interactions through a Long Detective Story of a Useful Azobis-Nitrile and Puzzling Racemate
Source: Cryst Growth Des. 2023 Jun 27;23(8):5719–33. doi: 10.1021/acs.cgd.3c00372 (PMC10402293; doi:10.1021/acs.cgd.3c00372)
Supplement: Supplementary file 1 — cg3c00372_si_001.pdf [file cg3c00372_si_001.pdf]

Supporting Information

Revisiting Homochiral versus Heterochiral Interactions Through a Long Detective Story of a Useful Azobis-nitrile and Puzzling Racemate

Juan García de la Concepción,[a,*] Mirian Flores-Jiménez,[a] Louis A. Cuccia,[b] Mark E. Light,[c]

Cristóbal Viedma,[d] and Pedro Cintas[a,*]

[a]*Department of Organic and Inorganic Chemistry, Faculty of Sciences, and IACYS-Green Chemistry and Sustainable Development Unit, University of Extremadura, 06006 Badajoz, Spain*

[b]*Department of Chemistry and Biochemistry, Quebec Centre for Advanced Materials (QCAM/CQMF), FRQNT, Concordia University, 7141 Sherbrooke St. West, Montreal, QC, H4B 1R6, Canada*

[c]*Department of Chemistry, Faculty of Engineering and Physical Sciences, University of Southampton, Southampton SO17 1BJ, UK*

[d]*Department of Crystallography and Mineralogy, University Complutense, 28040 Madrid, Spain*

CONTENTS

NMR and IR Spectra

Crystallographic Information

Computational Data

NMR and IR Spectra

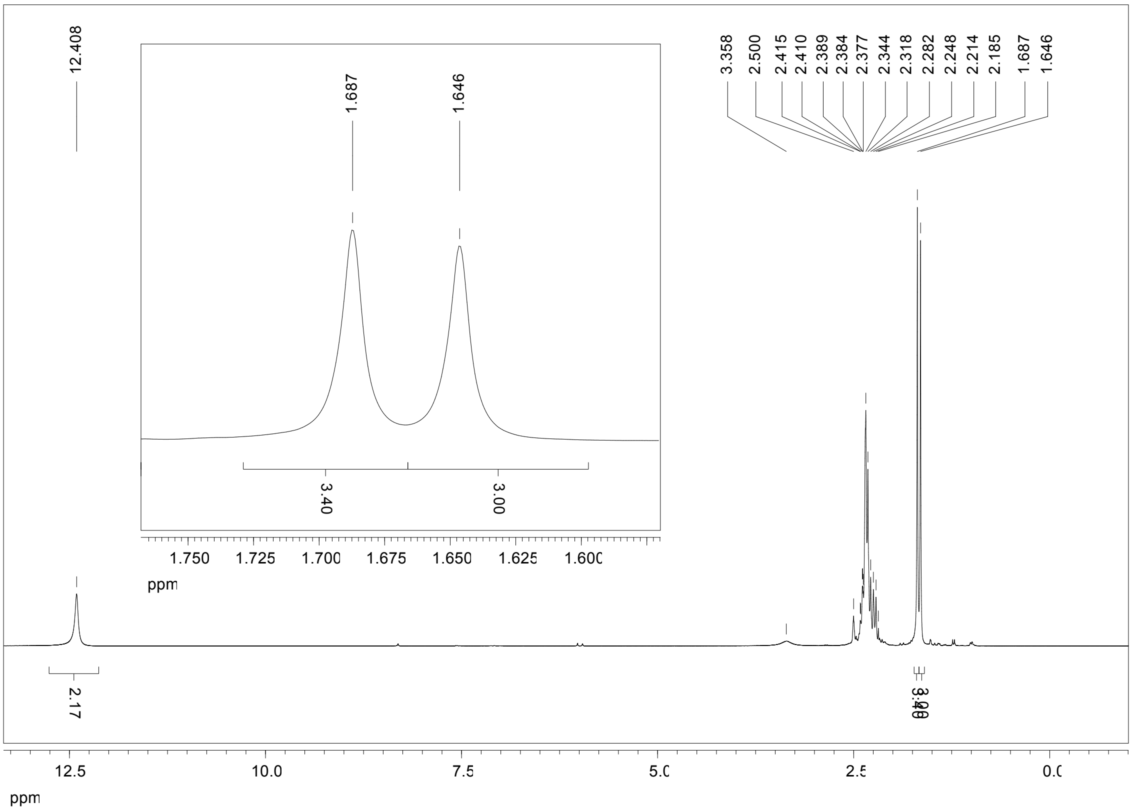

Figure S1. Proton NMR spectrum of ACPA in DMSO-d_6 at 300 MHz. Inset: magnification of diastereotopic methyl groups and signal integration to determine the *meso*/*d,l* ratio.

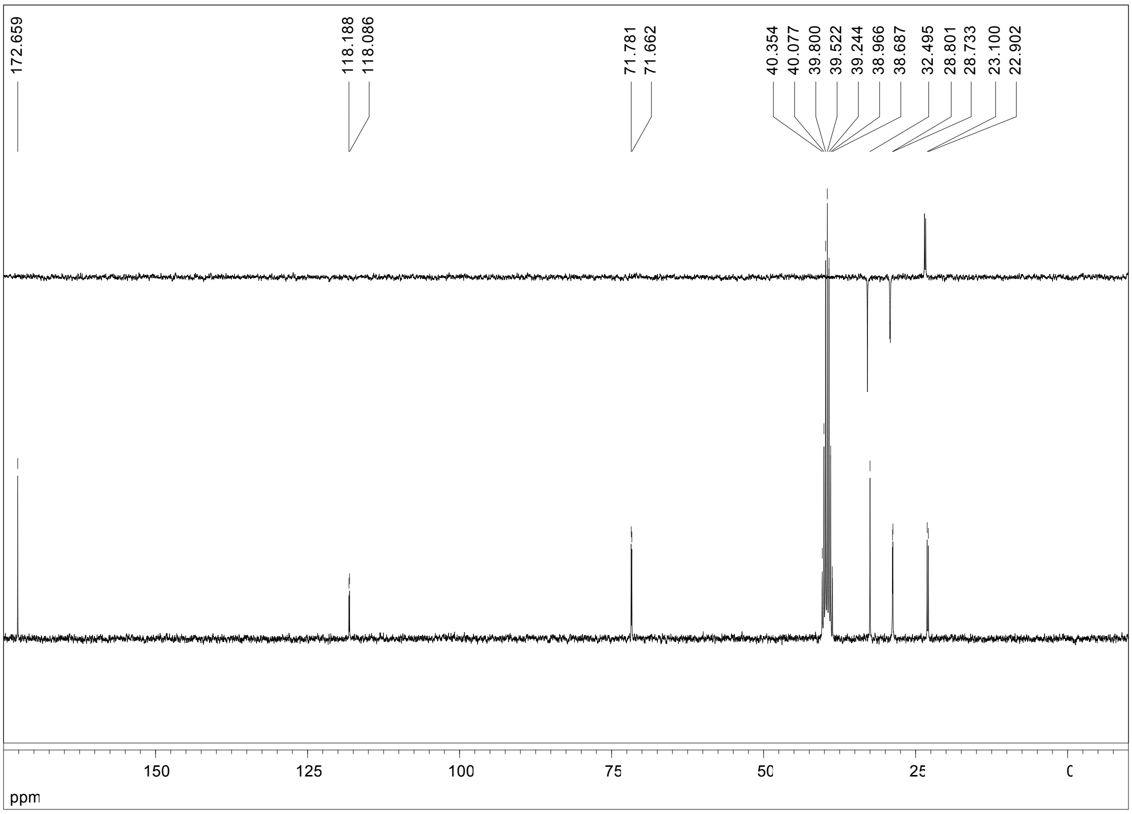

Figure S2. ^{13}C NMR spectrum of ACPA (Aldrich®) in DMSO-d_6 at 75 MHz. Top: DEPT experiment of polarization transfer.

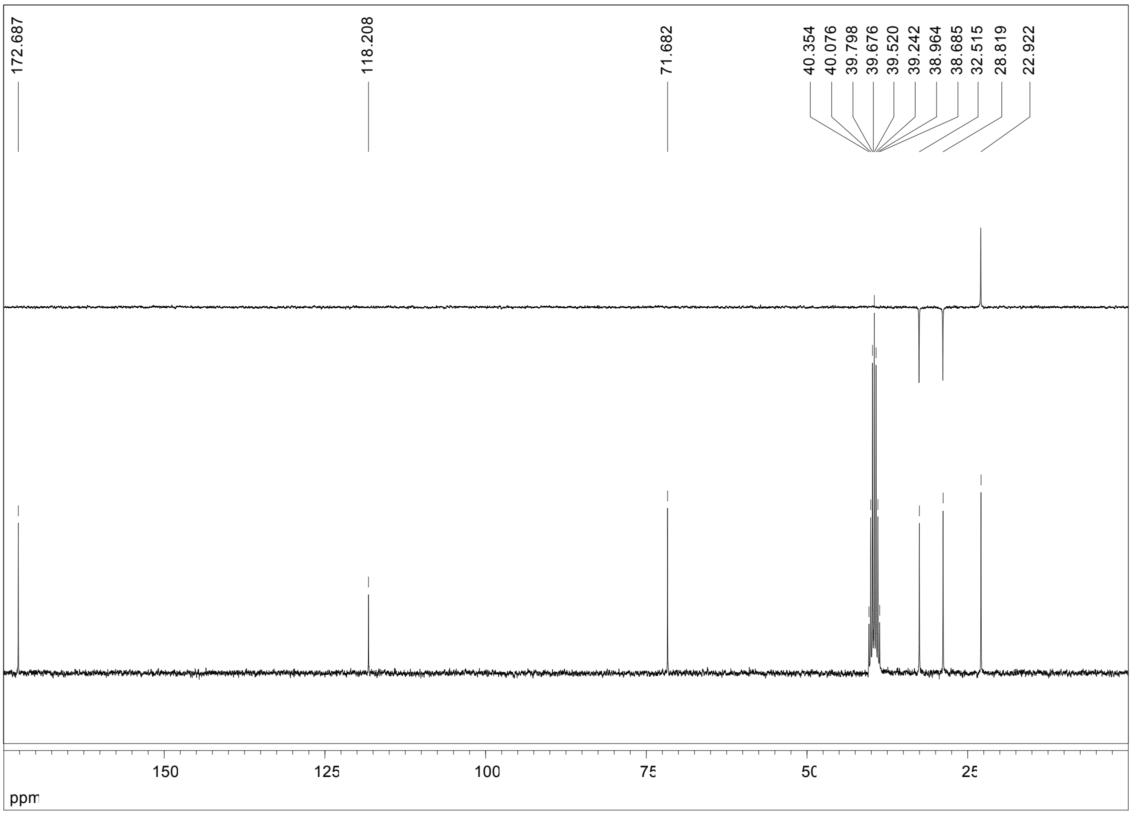

Figure S3. ^{13}C NMR spectrum of racemic-ACPA, (*d,l*)-stereoisomer, in DMSO-d_6 at 75 MHz (bottom); Top: DEPT experiment of polarization transfer.

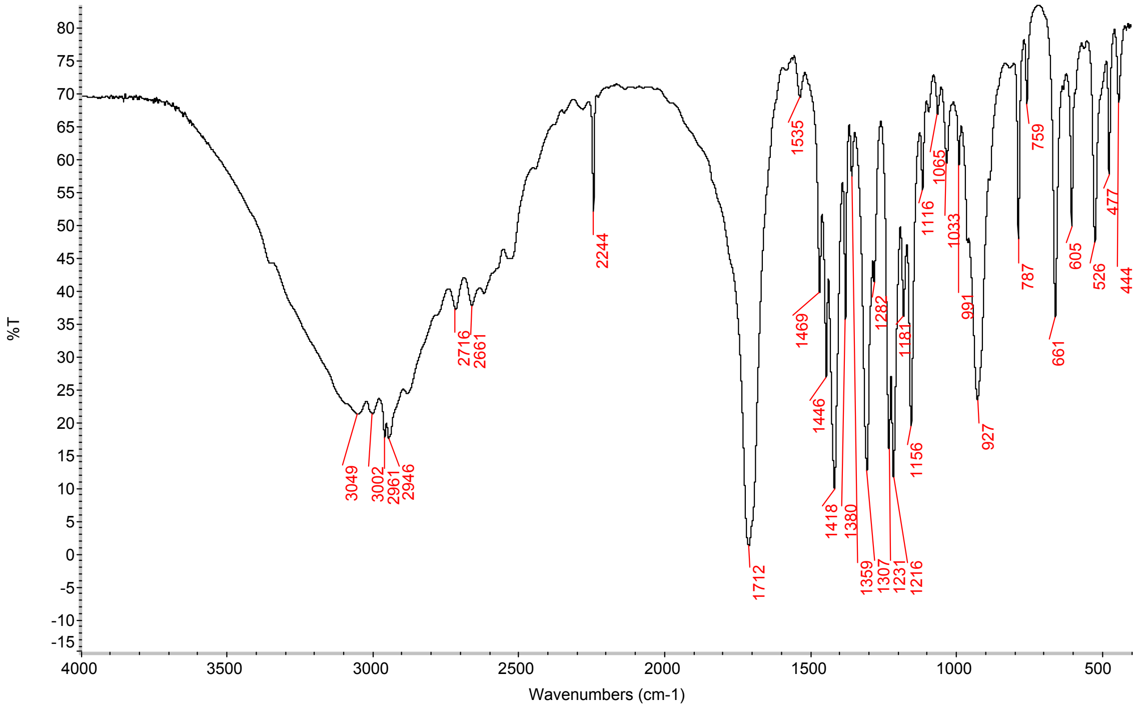

Figure S4. FT-IR spectrum of *meso*-ACPA recorded on a KBr pellet at ambient temperature.

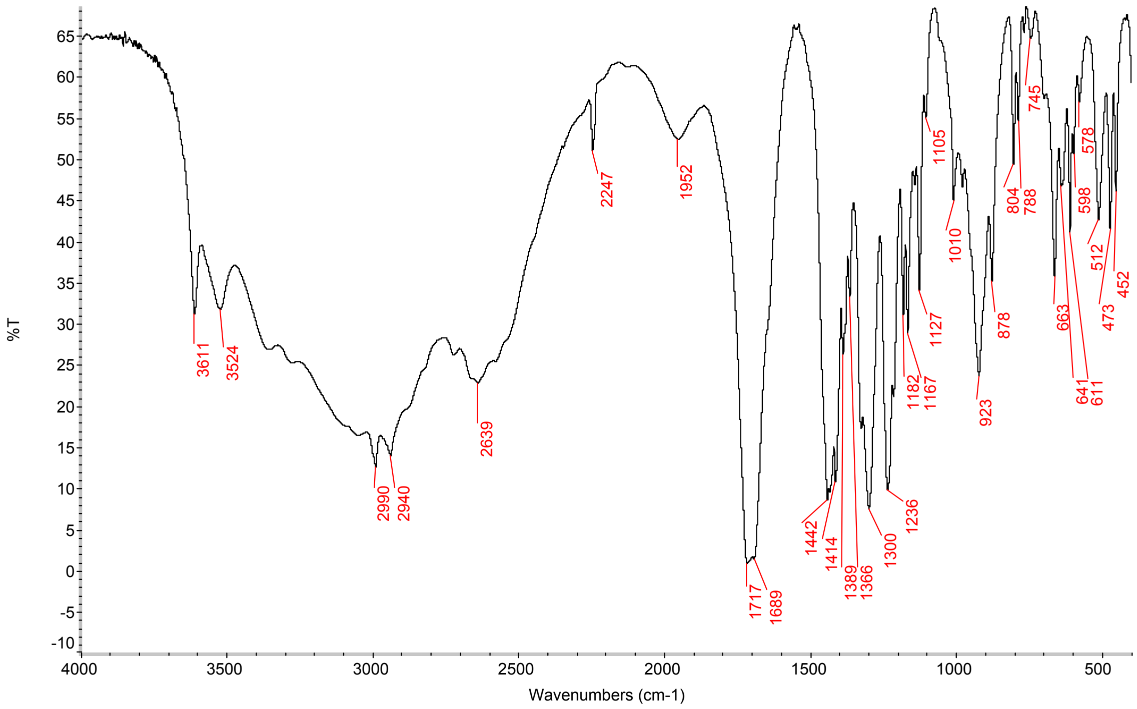

Figure S5. FT-IR spectrum of racemic ACPA (*d,l*-pair) recorded on a KBr pellet at ambient temperature.

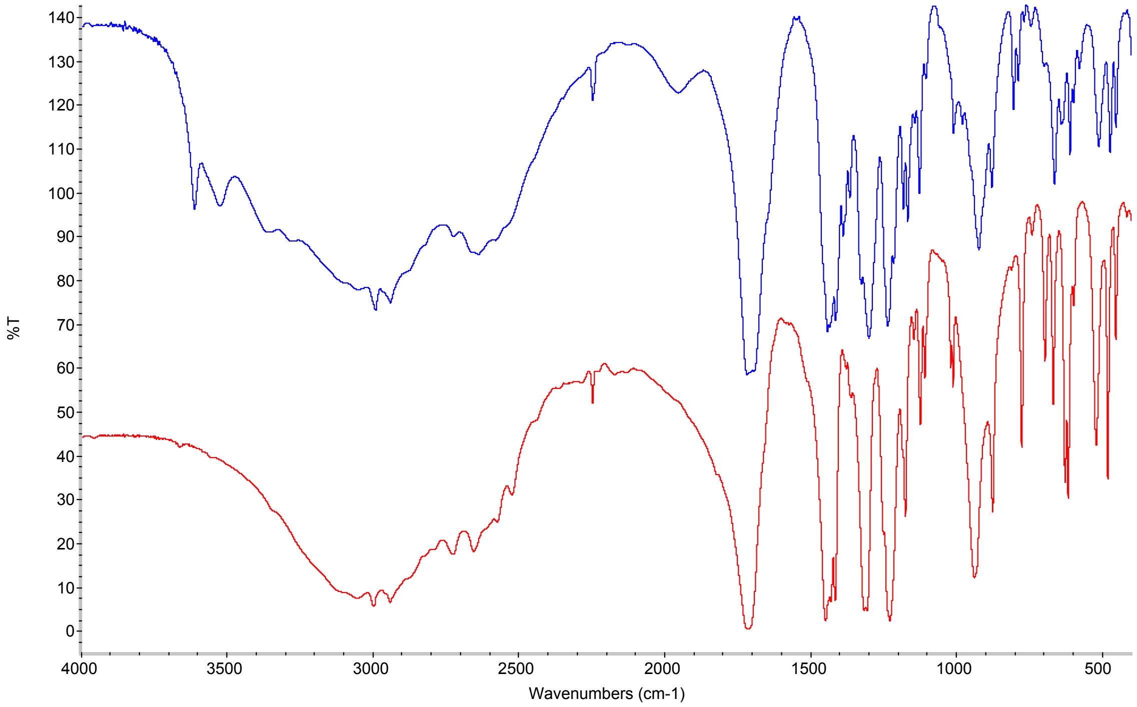

Figure S6. Comparative IR spectra (KBr pellets) of racemic ACPA (top) and a scalemic mixture enantioenriched in the levorotatory enantiomer (bottom).

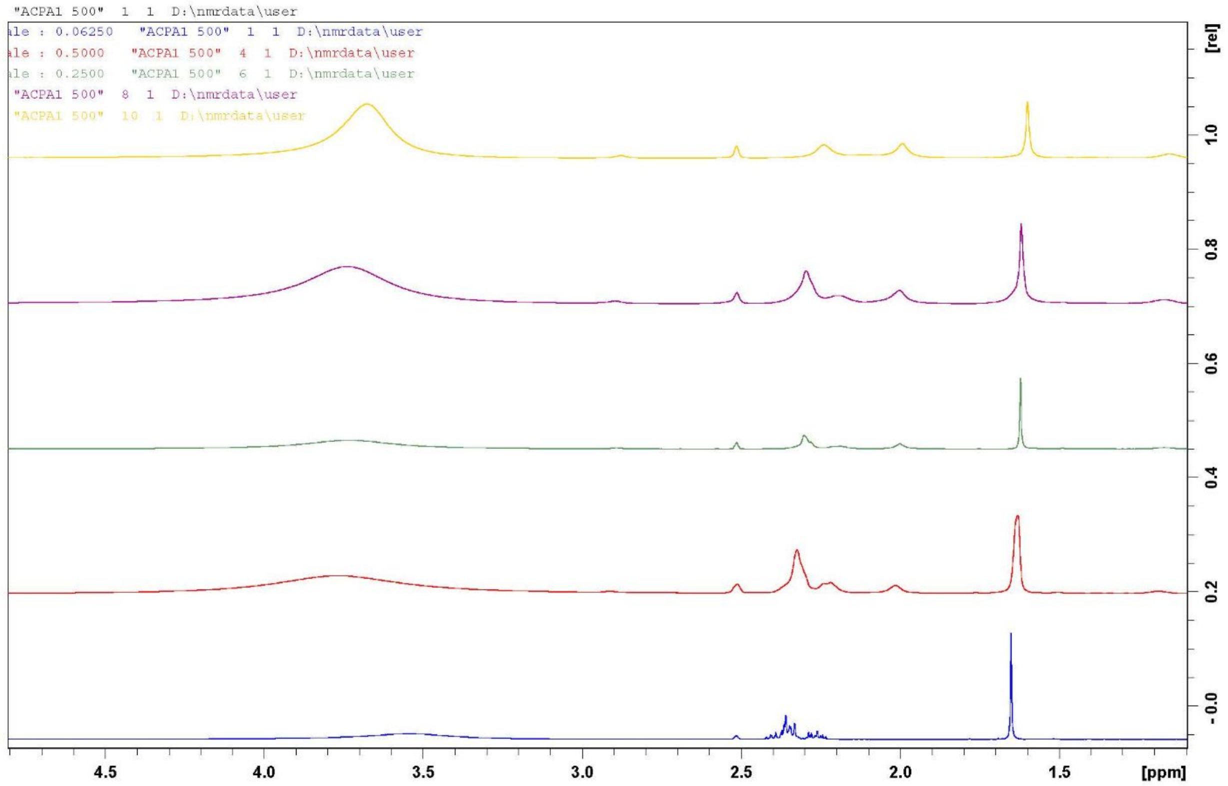

Figure S7. ^{1}H NMR spectrum of *(d,l)*-ACPA (enantioenriched in the levorotatory enantiomer) in DMSO-d_6 at 500 MHz (bottom), and spectra recorded after successive addition of Eu(hfc)$_3$ in 0.25:1, 0.5:1, 0.75:1 and 1:1 reagent:substrate molar ratios.

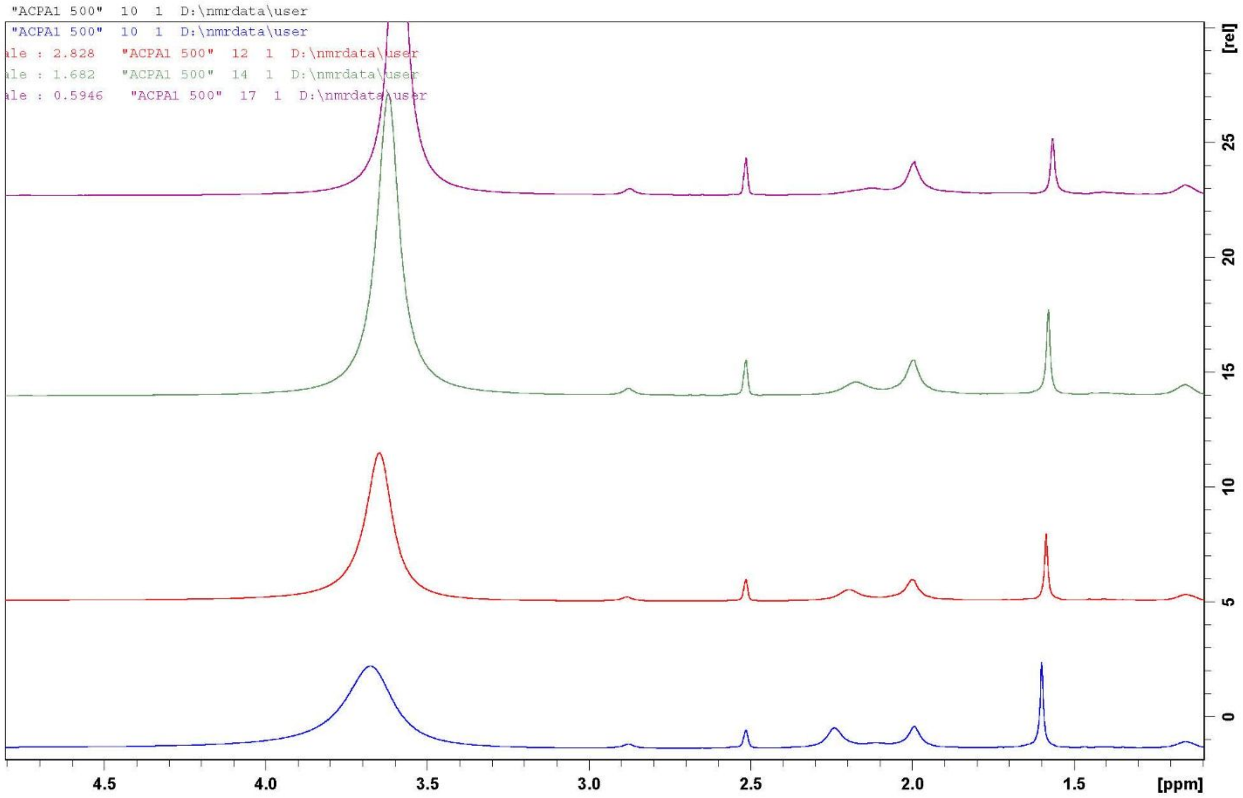

Figure S8. ^{1}H NMR spectrum of *(d,l)*-ACPA (enantioenriched in the levorotatory enantiomer) in DMSO-d_6 at 500 MHz (bottom), and spectra recorded after successive addition of Eu(hfc)$_3$ in 1.25:1, 1.5:1, 1.75:1 and 2:1 reagent:substrate molar ratios.

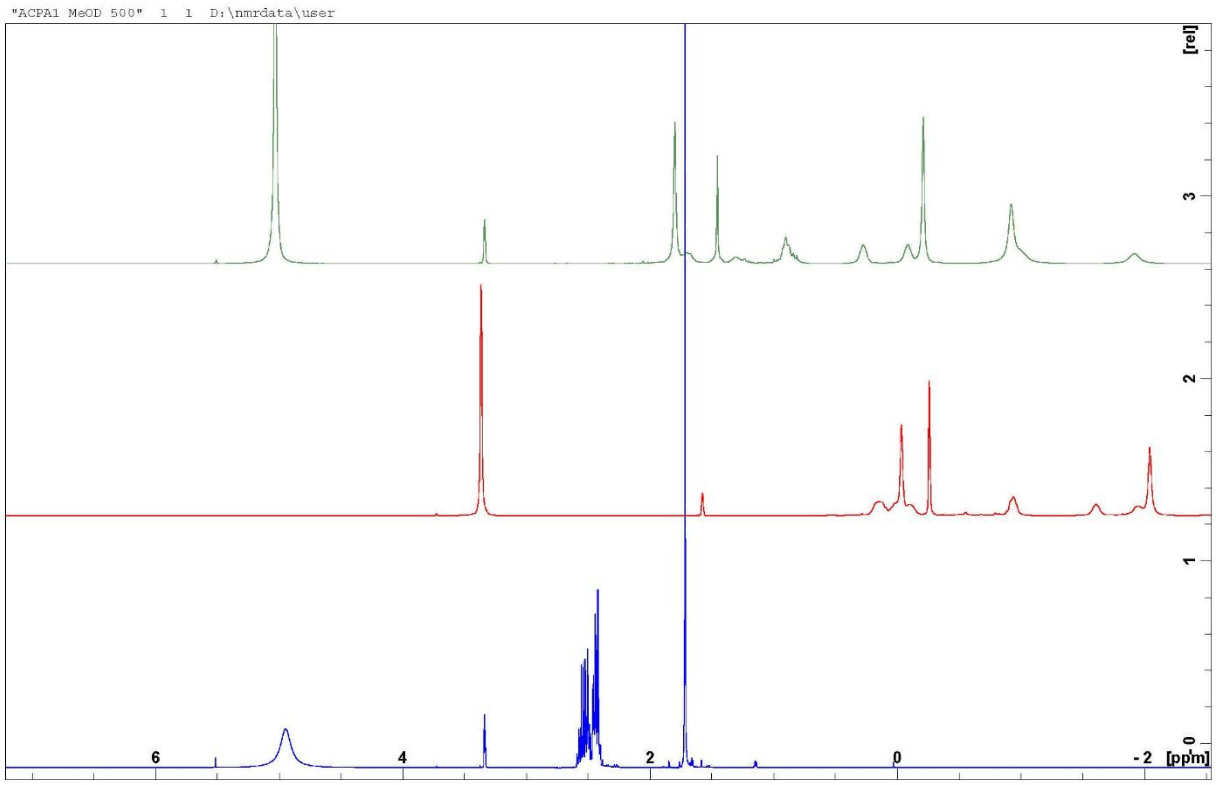

Figure S9. ^{1}H NMR spectrum of *(d,l)*-ACPA (enantioenriched in the levorotatory enantiomer) in CD_3OD at 500 MHz (bottom), and spectra recorded after successive addition of $Eu(hfc)_3$ in 1:1 and 2:1 reagent:substrate molar ratios.

Crystallographic Information

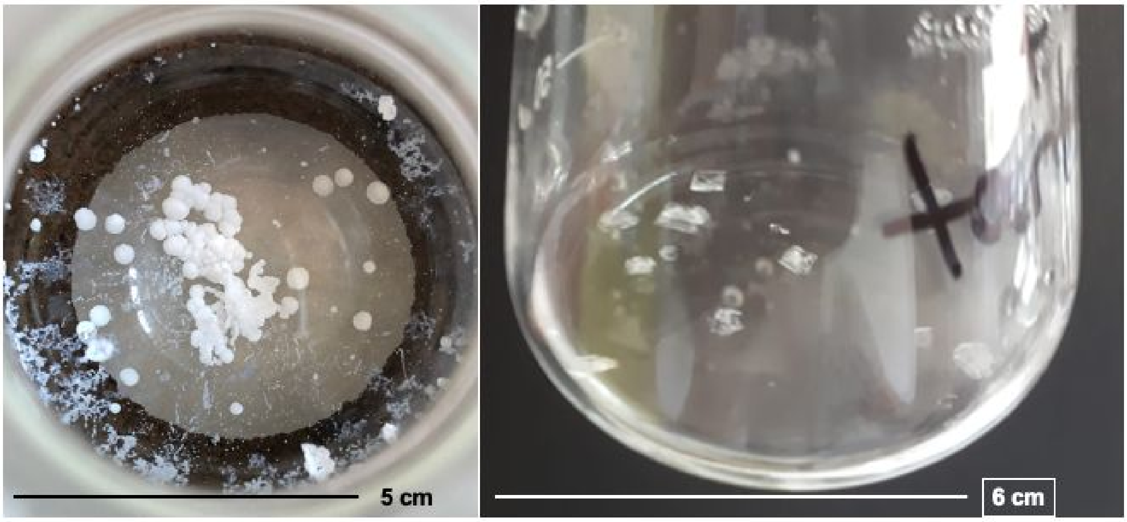

Figure S10. Left image: spherical aggregates of tiny ACPA crystals (d,l-racemate). Right image: larger (mm-sized) prismatic crystals of ACPA showing enantioenrichment.

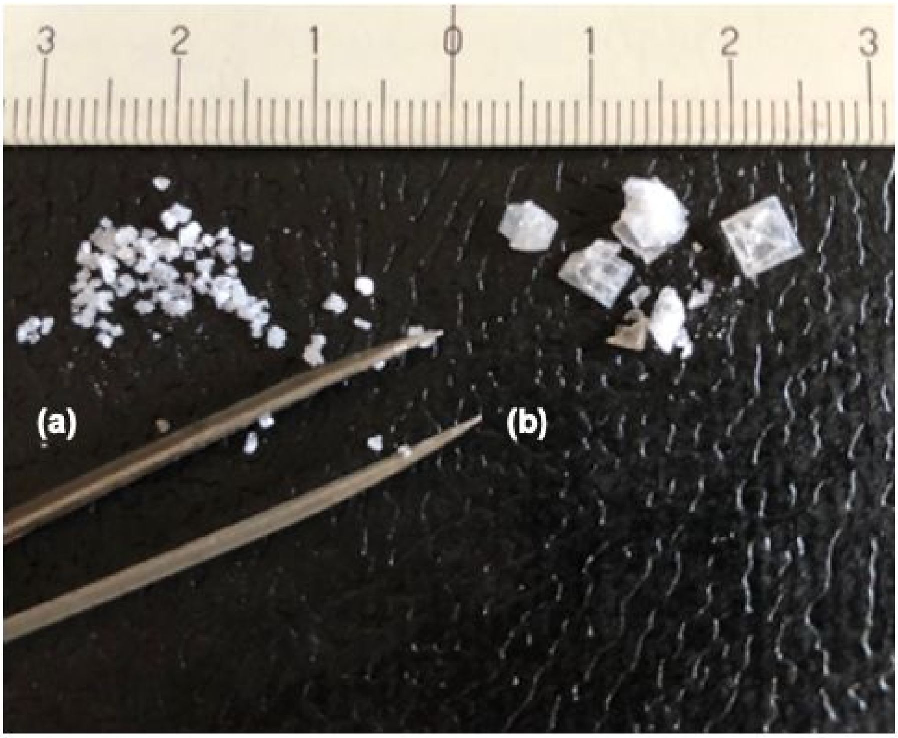

Figure S11. ACPA-crystals of arbitrary handedness, small and low *ees* (a) and large and high *ees* (b) obtained by spontaneous resolution of the *d,l*-racemate.

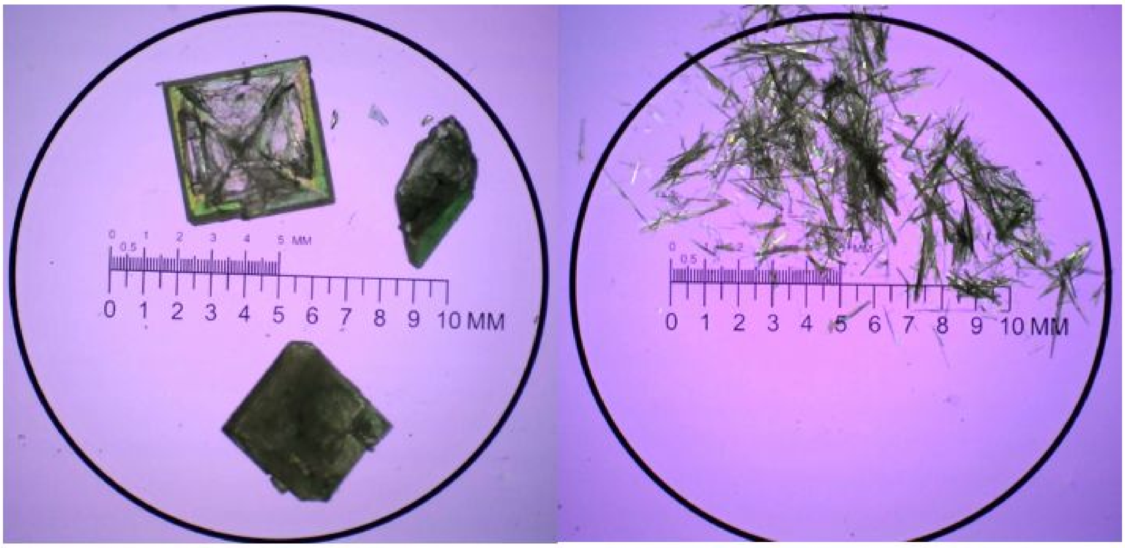

Figure S12. Observation under polarizing light (polarizer turned counterclockwise) of an enantioenriched mixture of ACPA prismatic crystals (left) and large thin crystals of the *meso*-stereoisomer (right), shown at 16.7x magnification.

Table S1: Fractional Atomic Coordinates (×10⁴) and Equivalent Isotropic Displacement Parameters (Å²×10³) for the (*R,R*)-configured ACPA enantiomer. U_{eq} is defined as 1/3 of the trace of the orthogonalised U_{ij}

Atom	X	y	z	U_{eq}
O1	3519.7(12)	1360.9(12)	5297.7(3)	30.7(3)
O2	5065.3(11)	2525.7(10)	4907.6(3)	22.4(2)
O3	10706.8(10)	4522.9(10)	5714.8(3)	20.8(2)
O4	9554.8(12)	2965.0(12)	5321.2(3)	31.2(3)
N1	4102.1(12)	6421.9(13)	6205.7(4)	25.3(3)
N2	5642.8(11)	3401.0(12)	6585.8(3)	16.6(2)
N3	6477.1(11)	4342.3(12)	6626.8(3)	16.1(2)
N4	7927.5(13)	1233.4(13)	6943.9(4)	25.0(3)
C1	4381.2(14)	2257.5(14)	5270.0(4)	17.6(3)
C2	4792.3(14)	3143.2(14)	5648.6(4)	18.2(3)
C3	3870.6(13)	2924.2(14)	6045.8(4)	17.1(3)
C4	4250.6(13)	3833.3(13)	6438.0(4)	16.0(3)
C5	4194.5(13)	5300.7(14)	6312.3(4)	18.8(3)
C6	3293.8(14)	3564.9(16)	6826.5(4)	22.6(3)
C7	7883.5(14)	2389.8(14)	6874.0(4)	17.6(3)
C8	8111.2(15)	4677.7(15)	7214.6(4)	21.7(3)
C9	7851.5(13)	3887.6(13)	6788.4(4)	15.9(3)
C10	8907.4(13)	4264.3(14)	6434.5(4)	17.4(3)
C11	8630.0(13)	3536.6(14)	6002.5(4)	17.4(3)
C12	9745.8(14)	3730.5(14)	5671.3(4)	18.5(3)

Table S2: Anisotropic Displacement Parameters (×10^4^) for (*R,R*)-configured ACPA enantiomer. The anisotropic displacement factor exponent takes the form: $-2p^2[h^2a^{*2} \times U_{11} + ... + 2hka^* \times b^* \times U_{12}]$

Atom	U_{11}	U_{22}	U_{33}	U_{23}	U_{13}	U_{12}
O1	32.0(6)	36.7(6)	23.5(5)	-11.9(5)	6.4(4)	-16.3(5)
O2	29.4(6)	20.9(5)	17.0(4)	-2.8(4)	1.7(4)	-4.6(4)
O3	19.3(5)	24.2(5)	19.0(4)	-0.4(4)	3.0(4)	-4.2(4)
O4	32.7(6)	40.9(7)	19.8(5)	-9.6(5)	9.8(4)	-16.7(5)
N1	18.7(6)	21.8(6)	35.2(7)	-1.5(5)	-0.1(5)	2.1(5)
N2	15.1(5)	19.5(6)	15.0(5)	-1.8(4)	-1.0(4)	-1.0(4)
N3	14.2(5)	19.3(5)	14.8(5)	-2.4(4)	1.9(4)	-0.7(4)
N4	27.5(7)	23.4(7)	24.0(6)	1.2(5)	-0.9(5)	0.0(5)
C1	16.1(6)	18.5(6)	18.2(6)	-1.2(5)	-1.8(5)	2.9(5)
C2	16.9(6)	19.5(6)	18.2(6)	-3.0(5)	-1.1(5)	-1.7(5)
C3	14.9(6)	18.0(6)	18.4(6)	-2.4(5)	-2.0(5)	-0.4(5)
C4	12.5(6)	17.1(6)	18.5(6)	-1.7(5)	-0.7(5)	0.1(5)
C5	14.0(6)	21.9(7)	20.4(6)	-4.7(5)	0.3(5)	0.6(5)
C6	18.4(6)	29.4(7)	19.8(6)	-2.9(6)	3.7(5)	-3.0(6)
C7	15.7(6)	23.6(7)	13.6(6)	-1.1(5)	-0.6(5)	-1.1(5)
C8	23.7(7)	23.5(7)	17.7(6)	-2.8(5)	-1.0(5)	-4.7(6)
C9	14.8(6)	17.0(6)	15.9(6)	-0.2(5)	-1.0(5)	-1.5(5)
C10	13.9(6)	20.8(7)	17.3(6)	0.7(5)	0.7(5)	-2.1(5)
C11	15.1(6)	21.3(6)	15.7(6)	1.1(5)	0.3(5)	-1.7(5)
C12	19.4(6)	20.1(6)	15.9(6)	0.8(5)	0.2(5)	0.4(5)

Table S3: Bond Lengths in Å for (*R,R*)-configured ACPA enantiomer

Atom	Atom	Length/Å
O1	C1	1.2160(18)
O2	C1	1.3190(16)
O3	C12	1.2224(18)
O4	C12	1.3180(17)
N1	C5	1.1449(19)
N2	N3	1.2334(16)
N2	C4	1.4922(16)
N3	C9	1.4962(17)
N4	C7	1.1489(19)
C1	C2	1.4986(18)

Atom	Atom	Length/Å
C2	C3	1.5252(18)
C3	C4	1.5363(17)
C4	C5	1.4831(19)
C4	C6	1.5327(18)
C7	C9	1.4848(19)
C8	C9	1.5342(18)
C9	C10	1.5379(17)
C10	C11	1.5228(17)
C11	C12	1.4982(18)

Table S4: Bond Angles in ° for (*R,R*)-configured ACPA enantiomer

Atom	Atom	Atom	Angle/°
N3	N2	C4	114.89(11)
N2	N3	C9	113.83(11)
O1	C1	O2	123.42(13)
O1	C1	C2	123.10(12)
O2	C1	C2	113.47(11)
C1	C2	C3	112.06(11)
C2	C3	C4	113.41(11)
N2	C4	C3	107.02(10)
N2	C4	C6	105.76(10)
C5	C4	N2	112.63(11)
C5	C4	C3	110.27(11)
C5	C4	C6	110.06(11)
C6	C4	C3	111.01(11)
N1	C5	C4	177.13(15)

Atom	Atom	Atom	Angle/°
N4	C7	C9	178.90(16)
N3	C9	C8	106.21(11)
N3	C9	C10	107.30(10)
C7	C9	N3	111.64(11)
C7	C9	C8	109.97(11)
C7	C9	C10	110.18(11)
C8	C9	C10	111.47(11)
C11	C10	C9	112.27(11)
C12	C11	C10	113.41(11)
O3	C12	O4	123.71(13)
O3	C12	C11	124.32(12)
O4	C12	C11	111.96(12)

Table S5: Torsion Angles in ° for (*R,R*)-configured ACPA enantiomer

Atom	Atom	Atom	Atom	Angle/°
O1	C1	C2	C3	-8.13(19)
O2	C1	C2	C3	173.27(11)
N2	N3	C9	C7	-1.43(16)
N2	N3	C9	C8	-121.29(12)
N2	N3	C9	C10	119.38(12)
N3	N2	C4	C3	128.92(12)
N3	N2	C4	C5	7.59(16)
N3	N2	C4	C6	-112.65(12)
N3	C9	C10	C11	-61.64(14)
C1	C2	C3	C4	-179.12(11)
C2	C3	C4	N2	-64.88(13)
C2	C3	C4	C5	57.94(15)
C2	C3	C4	C6	-179.83(11)
C4	N2	N3	C9	178.23(10)
C7	C9	C10	C11	60.09(14)
C8	C9	C10	C11	-177.53(12)
C9	C10	C11	C12	-172.06(11)
C10	C11	C12	O3	-8.67(19)
C10	C11	C12	O4	172.71(12)

Table S6: Hydrogen Fractional Atomic Coordinates (×10[4]) and Equivalent Isotropic Displacement Parameters (Å[2]×10[3]) for (*R,R*)-configured ACPA enantiomer. U_{eq} is defined as 1/3 of the trace of the orthogonalised U_{ij}

Atom	X	y	z	U_{eq}
H2	4826.64	1973.63	4710.83	34
H4	10182.06	3117.97	5139.28	47
H2A	4748.73	4117.5	5559.15	22
H2B	5752.14	2934.74	5730.12	22
H3A	3925.36	1951.01	6135.87	21
H3B	2909.96	3115.31	5961.02	21
H6A	3339.45	2594.13	6907.68	34
H6B	2351.48	3799.82	6744.53	34
H6C	3578.6	4129.15	7075.84	34
H8A	8042.09	5664.29	7158.36	32
H8B	9030.36	4462.41	7324.16	32
H8C	7426.91	4411.21	7433.44	32
H10A	8887.49	5267.48	6386.12	21
H10B	9835.69	4017.01	6538.84	21
H11A	8516.05	2544.09	6059.42	21
H11B	7758.18	3883.1	5879.03	21

Table S7: Fractional Atomic Coordinates (×10^4^) and Equivalent Isotropic Displacement Parameters ($Å^2×10^3$) for *meso*-ACPA crystal. U_{eq} is defined as 1/3 of the trace of the orthogonalised U_{ij}

Atom	x	y	z	U_{eq}
O1	2187.8(14)	5427.0(10)	737.1(7)	27.4(2)
O2	-96.3(13)	3678.0(10)	1144.5(7)	25.7(2)
N1	9530.5(17)	6236.1(12)	3891.2(9)	25.6(3)
N2	4739.1(15)	4559.4(10)	4635.5(8)	18.4(2)
C1	1670.3(18)	4442.6(13)	1334.9(10)	19.5(3)
C2	2964.4(18)	3966.4(13)	2360.7(10)	19.2(3)
C3	4909.6(18)	4918.3(13)	2610.0(9)	18.5(3)
C4	6230.3(17)	4488.7(12)	3687.5(9)	17.4(3)
C5	8065.2(18)	5495.9(12)	3836.6(9)	18.5(3)
C6	7108.6(19)	2973.7(13)	3658.5(10)	20.8(3)

Table S8: Anisotropic Displacement Parameters ($×10^4$) for *meso*-ACPA crystal. The anisotropic displacement factor exponent takes the form: $-2p^2[h^2a^{*2} \times U_{11}+ ... +2hka^* \times b^* \times U_{12}]$

Atom	U_{11}	U_{22}	U_{33}	U_{23}	U_{13}	U_{12}
O1	24.9(5)	33.1(5)	23.5(5)	6.8(4)	-8.8(4)	-5.6(4)
O2	21.1(4)	35.8(5)	19.7(4)	2.8(4)	-6.1(3)	-7.0(4)
N1	23.4(5)	31.2(6)	21.9(5)	5.3(4)	-3.3(4)	-4.2(5)
N2	18.6(5)	22.2(5)	14.0(5)	-0.5(3)	-1.9(4)	0.2(4)
C1	17.1(5)	24.6(6)	16.9(6)	-4.3(5)	-0.7(4)	1.3(4)
C2	17.3(6)	23.1(6)	16.9(6)	-1.1(5)	-2.3(4)	0.4(5)
C3	17.5(6)	23.4(6)	14.5(5)	0.1(4)	-2.2(4)	-0.3(4)
C4	16.0(5)	21.8(6)	14.1(6)	-1.1(4)	-1.0(4)	-1.2(4)
C5	19.1(6)	23.5(6)	12.6(5)	2.0(4)	-2.1(4)	1.8(5)
C6	21.2(6)	22.6(6)	18.4(6)	-0.9(5)	-2.0(4)	1.1(5)

Table S9: Bond Lengths in Å for *meso*-ACPA crystal

Atom	Atom	Length/Å	Atom	Atom	Length/Å
O1	C1	1.2199(15)	N2	N2[1]	1.2335(19)
O2	C1	1.3234(14)	N2	C4	1.4897(15)
N1	C5	1.1458(16)	C1	C2	1.4996(16)

Atom	Atom	Length/Å
C2	C3	1.5230(16)
C3	C4	1.5446(15)
C4	C5	1.4873(16)
C4	C6	1.5294(16)

----

[1]1-x,1-y,1-z

Table S10: Bond Angles in ° for *meso*-ACPA crystal

Atom	Atom	Atom	Angle/°
N2[1]	N2	C4	114.10(12)
O1	C1	O2	123.47(10)
O1	C1	C2	123.77(10)
O2	C1	C2	112.76(10)
C1	C2	C3	111.98(10)
C2	C3	C4	113.27(9)
N2	C4	C3	106.88(9)
N2	C4	C6	107.00(9)
C5	C4	N2	112.25(9)
C5	C4	C3	107.93(9)
C5	C4	C6	109.08(9)
C6	C4	C3	113.77(9)
N1	C5	C4	175.67(13)

----

[1]1-x,1-y,1-z

Table S11: Torsion Angles in ° for *meso*-ACPA crystal

Atom	Atom	Atom	Atom	Angle/°
O1	C1	C2	C3	3.91(16)
O2	C1	C2	C3	-176.23(10)
N2[1]	N2	C4	C3	-117.35(13)
N2[1]	N2	C4	C5	0.80(16)
N2[1]	N2	C4	C6	120.43(13)
C1	C2	C3	C4	177.87(9)
C2	C3	C4	N2	-58.90(12)
C2	C3	C4	C5	-179.84(9)
C2	C3	C4	C6	58.97(13)

----

[1]1-x,1-y,1-z

Table S12: Hydrogen Fractional Atomic Coordinates (×10^4) and Equivalent Isotropic Displacement Parameters (Å^2×10^3) for *meso*-ACPA crystal. U_{eq} is defined as 1/3 of the trace of the orthogonalised U_{ij}

Atom	X	y	z	U_{eq}
H2	-830(30)	4000(20)	486(19)	61(6)
H2A	3463.54	2981.27	2246.41	23
H2B	2033.87	3969.68	3016.79	23

**Atom**	**x**	**y**	**z**	U_{eq}
H3A	4407.61	5908.54	2691.33	22
H3B	5859.43	4886.55	1962.14	22
H6A	8056.56	2807.35	4326.16	31
H6B	7926.24	2843.35	2978.65	31
H6C	5905.81	2299.74	3651.97	31

Computational Data

Cartesian coordinates and thermochemistry for the optimizations of (E,R,R); (Z,R,R); (E,R,S) and (Z,R,S) azobis-nitrile.

(E,R,S)

```
:::::::::::::::::::::::::::::::::::::::::::::::::::::
::                 THERMODYNAMIC                   ::
:::::::::::::::::::::::::::::::::::::::::::::::::::::
:: total free energy        -65.308665626804 Eh   ::
::.................................................::
:: total energy             -65.532680279407 Eh   ::
:: zero point energy          0.274325782245 Eh   ::
:: G(RRHO) w/o ZPVE          -0.050311129642 Eh   ::
:: G(RRHO) contrib.           0.224014652603 Eh   ::
:::::::::::::::::::::::::::::::::::::::::::::::::::::
```

```
O       -4.719638245     -1.058929886     -0.035163401
H       -4.988215882     -1.908615999      0.337338557
O       -2.652165821     -1.869871271      0.091046064
N       -1.989323871      0.240408547      2.470691324
N        0.533669951      0.952973827      0.405162380
C       -3.410792585     -0.983622366     -0.228189769
C       -3.020522352      0.300216825     -0.919771530
H       -3.532192263      1.132512928     -0.433200581
H       -3.426693808      0.230154943     -1.931975258
C       -1.507871978      0.504392834     -0.991025810
H       -1.263439117      1.140544345     -1.842969198
H       -1.026922595     -0.462457992     -1.156413588
C       -0.921362494      1.157044699      0.274182144
C       -1.518648576      0.623689668      1.497287595
C       -1.102179250      2.683769129      0.260695060
H       -2.158614868      2.935657138      0.211395922
H       -0.593916160      3.090827973     -0.610748544
H       -0.669820328      3.118764546      1.159774804
O        0.360772172     -2.290402112     -1.381451824
O        0.009275946     -2.262542120      0.820456532
```

H	-0.938902737	-2.357415861	0.560331639
N	3.418327645	1.054809766	1.819498966
N	1.069267444	0.592190382	-0.657724796
C	0.779716514	-2.197597860	-0.251453893
C	2.244326891	-2.003924146	0.075943235
H	2.360031882	-1.689361294	1.114490239
H	2.727395045	-2.976019262	-0.044466165
C	2.888876382	-1.008820238	-0.894201068
H	3.974205006	-1.109513523	-0.860896102
H	2.552028477	-1.227695887	-1.910561049
C	2.526763064	0.451269208	-0.560292026
C	3.017753067	0.794157580	0.776229611
C	3.119897866	1.410556448	-1.599226831
H	2.723775656	1.162952273	-2.581520547
H	2.844740077	2.434277740	-1.353957827
H	4.203375073	1.321591574	-1.614324305

(Z,R,S)

```
:::::::::::::::::::::::::::::::::::::::::::::::::::::
::                    THERMODYNAMIC                 ::
:::::::::::::::::::::::::::::::::::::::::::::::::::::
:: total free energy         -65.293794817632 Eh   ::
::.................................................::
:: total energy              -65.519510128456 Eh   ::
:: zero point energy           0.274622735165 Eh   ::
:: G(RRHO) w/o ZPVE           -0.048907424341 Eh   ::
:: G(RRHO) contrib.            0.225715310824 Eh   ::
:::::::::::::::::::::::::::::::::::::::::::::::::::::
```

O	-0.833269664	2.832983552	0.141675665
H	-1.745276102	2.572115069	0.397280252
O	-0.209628717	1.445759840	1.775484714
N	3.024039218	-2.148722037	-0.435440875
N	0.588384294	0.319421227	-1.104893027
C	0.065968736	2.177016719	0.854028028

C	1.485575033	2.452674636	0.406914684
H	1.912323343	3.164730476	1.117006160
H	1.481630398	2.916299037	-0.579514297
C	2.322347675	1.170925458	0.440937944
H	2.131551123	0.653684458	1.383726639
H	3.382337290	1.427143491	0.405573411
C	2.025041910	0.231654599	-0.751606118
C	2.534466453	-1.111729478	-0.515674386
C	2.743790593	0.739019932	-2.017868263
H	2.397880608	1.742925114	-2.254027232
H	2.517631870	0.083253288	-2.856063119
H	3.819460136	0.758098876	-1.858525770
O	-2.953651932	1.116286673	0.090910239
O	-3.456260490	-0.061626574	-1.733973871
H	-3.695222839	0.790754824	-2.121297784
N	1.464215289	-1.433357970	2.217165537
N	-0.340620061	-0.345081350	-0.672019091
C	-3.068037635	0.050044266	-0.468831070
C	-2.875127899	-1.291714774	0.193985894
H	-3.756313146	-1.438826877	0.824398725
H	-2.871348820	-2.077982766	-0.560442545
C	-1.637974898	-1.339703444	1.093059994
H	-1.611616662	-0.432952866	1.704440499
H	-1.713341719	-2.200368724	1.759228700
C	-0.304409842	-1.445857005	0.308609660
C	0.738428972	-1.414074015	1.326277237
C	-0.229098338	-2.748168881	-0.503619773
H	-0.445898068	-3.596312343	0.141945043
H	-0.955268401	-2.718180957	-1.312705761
H	0.762094313	-2.870558627	-0.933306250

(E,R,R)

```
::::::::::::::::::::::::::::::::::::::::::::::::::::::::::
::                     THERMODYNAMIC                     ::
::::::::::::::::::::::::::::::::::::::::::::::::::::::::::
```

```
:: total free energy         -65.303641516938 Eh   ::
::.............................................::
:: total energy              -65.530072058529 Eh   ::
:: zero point energy           0.274552549109 Eh   ::
:: G(RRHO) w/o ZPVE           -0.048122007518 Eh   ::
:: G(RRHO) contrib.            0.226430541591 Eh   ::
:::::::::::::::::::::::::::::::::::::::::::::::::::::
```

```
O       -1.723271497     -0.951046627     -1.684872196
O       -3.223522603     -0.869380168     -0.031414591
H       -2.996755692     -1.819674442      0.094388832
O        1.723329605      0.950527072     -1.685367056
N        1.539553462      2.845985628      0.804336611
N       -0.593994033      0.185155263      0.705631505
N        0.593705921     -0.184886245      0.705304101
N       -1.539647717     -2.845492732      0.804862839
C       -2.466146219     -0.318799637     -0.971801927
C       -2.652720232      1.179793476     -1.068700504
H       -3.438991146      1.503175500     -0.387409530
H       -2.956909839      1.413041372     -2.090725853
C       -1.334381346      1.912210759     -0.791341497
H       -1.491026812      2.985974813     -0.900682360
H       -0.585582243      1.593214466     -1.522611380
C       -0.768787430      1.639118191      0.621649413
C        0.514558545      2.331718434      0.738677690
C       -1.716110381      2.061651887      1.747261628
H       -2.599344783      1.426197209      1.735960137
H       -2.014471746      3.099040482      1.617766968
H       -1.217353349      1.949114925      2.707870403
C       -0.514557921     -2.331579708      0.739417236
C        1.716140658     -2.060574508      1.747930851
H        1.217357553     -1.947583110      2.708471763
H        2.599274508     -1.424985583      1.736285301
H        2.014662386     -3.097987402      1.619015001
C        0.768688587     -1.638854947      0.622103616
C        1.334314493     -1.912571853     -0.790750182
```

H	1.491051328	-2.986372254	-0.899601297
H	0.585514116	-1.593966609	-1.522188335
C	2.652607914	-1.180171842	-1.068386726
H	3.438916179	-1.503328849	-0.387031352
H	2.956758874	-1.413745319	-2.090349886
C	2.466056172	0.318462397	-0.971986547
O	3.223318706	0.869325005	-0.031651546
H	2.996755078	1.819693657	0.093661306

(Z,R,R)

```
:::::::::::::::::::::::::::::::::::::::::::::::::::::
::                  THERMODYNAMIC                  ::
:::::::::::::::::::::::::::::::::::::::::::::::::::::
:: total free energy         -65.294207277843 Eh   ::
::.................................................::
:: total energy              -65.519654157044 Eh   ::
:: zero point energy           0.275015030643 Eh   ::
:: G(RRHO) w/o ZPVE           -0.049568151442 Eh   ::
:: G(RRHO) contrib.            0.225446879201 Eh   ::
:::::::::::::::::::::::::::::::::::::::::::::::::::::
```

O	-3.753690401	-1.339300826	1.391328540
H	-3.677166976	-2.299206890	1.470640212
O	-2.896904415	-1.636026805	-0.646548470
N	-1.116354642	1.641765100	-2.132558491
N	-0.765123215	-0.212487262	0.912214491
C	-3.350655983	-0.906585055	0.199376145
C	-3.553550925	0.581323938	0.034978104
H	-4.556471259	0.827181923	0.386619351
H	-3.474960399	0.831786271	-1.024028353
C	-2.540803598	1.390577026	0.857157002
H	-2.627818504	1.120241240	1.911748598
H	-2.771309506	2.451557953	0.750581247
C	-1.076797730	1.144354527	0.419144561
C	-1.019048643	1.383355048	-1.016298082

C	-0.146845419	2.090580236	1.194611259
H	-0.326834585	1.967264274	2.260788421
H	-0.353541527	3.120811492	0.913572214
H	0.896111197	1.860752271	0.986360033
O	3.003066013	0.843568115	1.591853981
O	4.495244042	0.042332690	0.140798123
H	4.922882382	0.905187727	0.234008624
N	1.873703262	1.462191315	-1.380207984
N	0.057155811	-1.000100376	0.471684359
C	3.416426725	-0.062332069	0.911835314
C	2.796430366	-1.438221078	0.854364724
H	3.590410714	-2.169372357	1.012832297
H	2.064729471	-1.524314415	1.659576015
C	2.125245452	-1.739263971	-0.492490566
H	2.840188489	-1.606833039	-1.306862797
H	1.802617581	-2.781943889	-0.483814712
C	0.879984140	-0.854533574	-0.745651149
C	1.364243785	0.477334132	-1.077128235
C	0.047994844	-1.454065305	-1.890369720
H	-0.254966383	-2.462883861	-1.614876512
H	-0.846806252	-0.860632206	-2.067988473
H	0.646396115	-1.491857694	-2.798669034

Cartesian coordinates and thermochemistry for the smooth optimizations of all molecular aggregates

RR-RR

```
:::::::::::::::::::::::::::::::::::::::::::::::::::::
::                   THERMODYNAMICS                  ::
:::::::::::::::::::::::::::::::::::::::::::::::::::::
:: total free energy       -1371.571599729226 Eh   ::
::.................................................::
:: total energy            -1376.834686130813 Eh   ::
:: zero point energy           5.788824828921 Eh   ::
:: G(RRHO) w/o ZPVE           -0.525738427334 Eh   ::
:: G(RRHO) contrib.            5.263086401587 Eh   ::
```

::

O	1.50493321137313	1.48820972572350	-4.49031047236237
O	1.29190869401211	0.97408313695026	-2.32925498539064
H	2.25175258864006	0.87143499394668	-2.33467247799413
O	-7.55398605767029	2.56640442967335	-0.28813042290520
N	-3.76009627982888	1.03555159067992	-5.33842921595359
N	-3.29291423007407	1.37429855105582	-2.07054324043950
N	-3.24069270552903	2.50180869546707	-2.59679807034122
N	-3.66455694264311	5.79528477863956	-2.95862346446583
C	0.81053744576109	1.29887532077555	-3.52187096317250
C	-0.69469810451023	1.41384470667393	-3.53095350189108
H	-1.02385631947873	1.57369482612424	-4.56046739631539
H	-0.96299373375913	2.30075213200703	-2.94702217286605
C	-1.37246776158126	0.18046131704264	-2.93470567560221
H	-1.07912065731702	0.05807405969777	-1.89145642297999
H	-1.07514346988690	-0.71674504796997	-3.47936987725346
C	-2.90790288515629	0.29790464836228	-2.99345060061292
C	-3.35830798823637	0.66269257449736	-4.33117586681964
C	-3.58335271701358	-0.98657640753615	-2.51856062163297
H	-3.27358542194132	-1.19288214523565	-1.49775034180532
H	-3.29715411018743	-1.82104762465475	-3.15429537154482
H	-4.66591127852360	-0.86930098114395	-2.54169698498153
C	-3.63851993532445	4.81514257985126	-2.36389877783263
C	-2.51251517557860	3.61017055356612	-0.55493424934817
H	-1.55144425657713	3.85520099009654	-1.00480322319015
H	-2.44449850610540	2.63379172162341	-0.08167543802394
H	-2.75972971631629	4.36157467140918	0.19026926959014
C	-3.60116818709932	3.55449815124930	-1.62858888757695
C	-4.97684447781115	3.22687668288878	-1.02254067117225
H	-4.86165855589290	2.36087936766097	-0.36908361405545
H	-5.31441855060942	4.06577725991226	-0.41076164077417
C	-6.01271511580047	2.90972940435701	-2.09988374607198
H	-6.15527352927127	3.76192422455848	-2.77555726873760
H	-5.70161271077120	2.04902955644932	-2.70211170832623
C	-7.34998146242589	2.57220197268722	-1.49153480223701

O	-8.26748269449652	2.28030878121095	-2.37582455001153
H	-9.13049907055041	2.08825329635250	-1.90697839843860
O	-2.24697235538007	12.31224497069999	-9.73640279861825
O	-0.09863550924031	12.82796413124659	-9.41560865948592
H	-0.39786438723046	13.77081637082896	-9.51942845595639
O	-0.00976192066986	6.11317479293287	-8.36070302055304
N	-3.55857156260517	6.85169473727441	-10.65361913827728
N	-0.72795838311874	8.60913639136552	-11.59888526164806
N	-0.11985674198660	7.70523186306759	-10.99389367213299
N	1.70647262809154	9.15115391634626	-13.47984678519319
C	-1.09208672123400	11.98185200177054	-9.59298851600217
C	-0.61578121557102	10.54666972816239	-9.58151900032547
H	-0.00867412408983	10.41239296877288	-8.68404600815286
H	0.05693504100278	10.42193346541400	-10.43465933272660
C	-1.75542088676345	9.52438124533282	-9.60443629727683
H	-2.67106243440847	9.97406906459054	-9.20642115704150
H	-1.47776838273512	8.68879198380840	-8.95977305073255
C	-2.04402301273882	8.94092012588948	-11.00390720556303
C	-2.86373535950757	7.74708847441745	-10.82318502193121
C	-2.75973592091099	9.91067754406193	-11.94617674608013
H	-2.78696974875500	9.49804058401480	-12.95562009930295
H	-2.22508059329793	10.85690773610364	-11.97901066517999
H	-3.77992358796221	10.09903142311425	-11.60438321776553
C	1.51372931067432	8.45088844061902	-12.59088971627882
C	1.43757916981398	6.09552597105305	-11.98195886586063
H	0.90066575737091	5.95739509821038	-12.91969352404971
H	1.05582577233057	5.38970437762543	-11.24734313894287
H	2.49314332926008	5.90321268689519	-12.15140524349230
C	1.24740551241092	7.52876392994533	-11.48282635775261
C	2.19285837059609	7.87979500565844	-10.31483770495510
H	3.21356399808092	7.86504481100224	-10.69593240601292
H	1.97215630840966	8.89465849621038	-9.97449033649193
C	2.11556055568427	6.91541635654092	-9.12407216755433
H	2.26829870384452	5.88491722782760	-9.44293022898947
H	2.91388354710257	7.18510035969901	-8.43229319354532
C	0.81514930775606	7.01149952858059	-8.36976684232135

O	0.66725806187579	8.13225039091964	-7.71129351761182
H	-0.18428433913844	8.14179494185556	-7.16132272440158
O	-1.59574268732021	-2.18283917981730	-11.69848543608820
O	-1.99929446047913	-2.69066411370976	-9.57959824298135
H	-2.77473655036468	-3.21172188976760	-9.97987595872276
O	-0.95697126752000	4.16865131184199	-4.64839786917506
N	-2.54220441268699	-0.34898750985784	-7.63807213444828
N	0.76191158430484	0.42244706519780	-7.84725920818825
N	0.15524675225369	1.23680974239616	-8.56641947394507
N	-0.34886133983916	3.96365943066452	-10.43607297206343
C	-1.28127341061736	-2.13802846109961	-10.50971211636086
C	-0.01810467519185	-1.45009045891813	-10.06740945418094
H	-0.17994082025539	-0.36632659020090	-10.14967066687163
H	0.74599655903103	-1.69999049665333	-10.80484789695640
C	0.45668836809344	-1.82362839710857	-8.66219591937762
H	1.54653747051752	-1.85666149169362	-8.64987708207195
H	0.08527818946939	-2.81066777711161	-8.38128919171307
C	0.02143347912616	-0.81287600051415	-7.58229731396403
C	-1.41729187444538	-0.57293342978613	-7.62067473808560
C	0.45169191916632	-1.29121991723884	-6.19548739834304
H	1.52850315838631	-1.43996756587601	-6.19168857681213
H	-0.04090396529174	-2.22599749610600	-5.94470079543507
H	0.20107686646469	-0.53989044372753	-5.45042408145560
C	0.25219592501194	3.29482770965461	-9.72252511536118
C	2.32350339522796	2.03241569660781	-9.41356695286428
H	2.94955631368455	2.90735633111395	-9.56939197006724
H	2.15655644934981	1.53614397477515	-10.36785868026301
H	2.82694502713133	1.34195820319600	-8.74101462369735
C	0.98645583132079	2.44272445068908	-8.79450350581411
C	1.17877597117961	3.16486651810088	-7.44391046344988
H	1.79755273011627	2.53047286360618	-6.80633142466148
H	1.70398967585116	4.10631626061484	-7.61469536705388
C	-0.16937989397953	3.41066466393830	-6.77034538925165
H	-0.84287547495037	3.92161268554999	-7.46849638993780
H	-0.63410954490469	2.45854611594569	-6.50351443163871
C	-0.13524826782428	4.27676262740676	-5.53131203037142

O	0.82074477049834	5.18543246051675	-5.52827483197070
H	0.80020330392004	5.71390925493919	-4.69071167563456
O	-4.01806330731880	-4.00917136805618	-10.59795797754649
O	-3.22928599382049	-4.00384983044581	-12.68118765515742
H	-2.54233621885788	-3.35706037486051	-12.32083743570810
O	-13.17238790352775	-1.50629669937872	-13.86931579963777
N	-5.20864080972312	-1.60595062266859	-12.89054189382657
N	-7.53672395239343	-3.92330040836977	-12.51323792179005
N	-8.70099699412282	-3.97205836836757	-12.93887628423354
N	-9.28579406008107	-2.55710000946718	-9.95072666442112
C	-4.07061626415991	-4.38444277541962	-11.75962037638055
C	-5.11127283215742	-5.36059190688284	-12.24111851634933
H	-5.90021408294748	-5.44205543021361	-11.49211245519906
H	-4.61434778825737	-6.32966159379769	-12.31557891457308
C	-5.68472933132289	-5.00312812726567	-13.61322369317435
H	-6.32055010315721	-5.82186406377956	-13.95082875127099
H	-4.87639886844927	-4.87479857626261	-14.33462730873633
C	-6.54720922955292	-3.72651259062399	-13.58887325045629
C	-5.76968315745085	-2.55729870055683	-13.19551401131596
C	-7.18199502033405	-3.47147679616663	-14.95788428634617
H	-6.39871247782147	-3.32204380868933	-15.69709918532054
H	-7.82183428932242	-2.58938973797417	-14.92402088585805
H	-7.78394098752169	-4.33062049614427	-15.24231950838238
C	-9.47292317531909	-3.32702011678845	-10.78064163633930
C	-9.42169041158737	-5.70175311710895	-11.39470233742355
H	-10.13154355014293	-5.97187815885565	-10.61736049994266
H	-8.40781612406743	-5.77469823297838	-11.00602051907470
H	-9.52801017736657	-6.38485659951836	-12.23356214730538
C	-9.67885293231311	-4.27011639182329	-11.87281560433469
C	-11.07862201684232	-4.11131336246032	-12.48124475923981
H	-11.19677543524065	-4.87113284873874	-13.25464116762564
H	-11.83369413395187	-4.28530045954274	-11.71329005823922
C	-11.25362517429048	-2.72213649222555	-13.09891804747554
H	-11.16254539092062	-1.93639123517404	-12.34355777150553
H	-10.47082384846298	-2.55872848106775	-13.84886254606253
C	-12.57687229982846	-2.55494975800235	-13.81013686233582

O	-12.99831905559134	-3.66962192561456	-14.38774441974702
H	-13.83282604021058	-3.56428494846233	-14.86024992996787
O	-1.03064320840250	-4.16882384358644	-6.64773356261733
O	-2.53316543681041	-5.54389712171760	-5.74796035109766
H	-1.77841195050074	-6.12840702673978	-5.60302291838317
O	-9.82065357860511	-0.12215626363503	-5.86097688108518
N	-4.93430127229917	-1.31126813331341	-9.34485192369838
N	-6.19840962630408	-2.60256658943268	-6.58321156711866
N	-6.20607205891426	-3.35777128252906	-5.59461146128404
N	-6.62457003924740	-0.26381805314256	-4.20017275742032
C	-2.17231418995750	-4.41274804002889	-6.34197941726790
C	-3.33684153715722	-3.48002648247068	-6.58226901664855
H	-2.97662467034510	-2.59475238327962	-7.11239718856307
H	-3.73114249723597	-3.17601801987259	-5.60799592588854
C	-4.43228760657309	-4.19672991786324	-7.37594459901791
H	-4.83179517463920	-5.01568581830231	-6.77614523867805
H	-4.01725534760113	-4.61946003835365	-8.29208364967139
C	-5.59157902192470	-3.26671563666449	-7.75673980728556
C	-5.17874154766603	-2.19758040164777	-8.66043567591501
C	-6.71352728908695	-4.07778036187860	-8.41798336945122
H	-7.55608899283313	-3.43555854841515	-8.67268968835759
H	-7.04905482124795	-4.84272099516114	-7.72212488041399
H	-6.33721885045167	-4.55305441321119	-9.32146426582232
C	-6.79035274250214	-1.39464505039744	-4.30418508081069
C	-6.40099534568267	-3.54218293062113	-3.19699987513423
H	-6.53405915565578	-4.61527534613971	-3.30804008987237
H	-6.92613411513736	-3.20519999162280	-2.30675437773514
H	-5.33940809079529	-3.33111044119196	-3.08958495217296
C	-6.94855754963459	-2.83808085889742	-4.43976307226033
C	-8.41546467690314	-3.25068722081529	-4.69275387758059
H	-8.39877840132517	-4.29884851465589	-4.99474256585695
H	-8.96820732222045	-3.18253162726196	-3.75502446644444
C	-9.10369611878402	-2.41799603338349	-5.77689848297411
H	-8.40620086190104	-2.15455503074103	-6.57711949594421
H	-9.90107847265418	-3.01983409468922	-6.22039365972599
C	-9.78694859515428	-1.16905492071485	-5.25175752678132

O	-10.37397484879309	-1.38594000824589	-4.09765453357771
H	-10.99534999426978	-0.65489664545286	-3.77049645098012
O	-10.65610247041906	10.74265373068176	-12.49233605853514
O	-11.90634584367850	12.55330194457763	-12.87257183948075
H	-12.26052367051917	12.02841227474504	-13.64665871695392
O	-10.18407336121327	12.44274310200451	-4.02883719790337
N	-7.27897399004523	13.66374443114003	-9.87062444028893
N	-10.26085896259979	14.93603899109400	-9.02158537495724
N	-9.95497996270845	14.01431028424205	-8.24650689186244
N	-11.73526412853625	11.81580212107251	-6.55160062010976
C	-10.98029022899829	11.87617736336740	-12.22068899643375
C	-10.36679645826514	12.65628443029812	-11.08094617540684
H	-9.35063777675122	12.28073616589151	-10.93011338477754
H	-10.94596360082274	12.43389756346712	-10.17859478825132
C	-10.36564489133518	14.16351504309472	-11.33248632659159
H	-11.38141710972821	14.55861621890746	-11.29662168211878
H	-9.95831642557707	14.37707852735541	-12.32079065708905
C	-9.51813452753886	14.91999676507517	-10.29074846121362
C	-8.24375497118614	14.24708773196548	-10.08334571529047
C	-9.30004202138674	16.37785571570294	-10.69430611607251
H	-8.76884038895923	16.90383345630097	-9.90469343276380
H	-10.26470040160292	16.85395231217068	-10.84727495466051
H	-8.72146604093535	16.43382645517096	-11.61298541796956
C	-11.25412288260655	12.84993411800086	-6.68559934480928
C	-11.71519068815134	15.23141222624892	-6.95464410402015
H	-12.36566870730955	15.09791215971489	-7.81590769901664
H	-11.24384551475798	16.20751958032723	-7.03004716265368
H	-12.31074405794300	15.18386568498010	-6.04723912355890
C	-10.64253281341754	14.14401896140615	-6.94389699608878
C	-9.48105605032906	14.44844324701604	-5.97060829401845
H	-8.79577160406200	13.59464526197131	-5.95781768298036
H	-8.94863065954111	15.30800141402273	-6.38082147310827
C	-9.91830532940611	14.77807971881091	-4.54806348126493
H	-9.03643454559277	15.09381801928871	-3.98315091601956
H	-10.62938524830498	15.60145920306090	-4.51304170333166
C	-10.48013975246850	13.59919289069370	-3.79557332474522

O	-11.29998677963325	13.96763483933232	-2.84323386621942
H	-11.61942697992425	13.20734247885260	-2.28175621821637
O	-5.00317163246850	11.04561892169764	-17.57433386362651
O	-4.70336102142037	11.29964719312984	-15.38128816429815
H	-5.12179998470372	12.20691109751075	-15.55447475742014
O	1.28324386611772	3.40640329742424	-17.47483505878935
N	-5.06588169140682	6.18057023195586	-15.21375119214372
N	-2.29219187090273	6.82987182249060	-16.90363459454149
N	-1.56049257088667	7.69313767170644	-17.41783504152813
N	-0.28789966274701	7.00271813553181	-14.49895900026578
C	-4.57331878933713	10.64275774369229	-16.50560735503676
C	-3.83842844403559	9.33490905805322	-16.37025644492991
H	-4.13092857590401	8.85866222526993	-15.42746737764759
H	-2.77226910067346	9.57544173417234	-16.29678936103209
C	-4.09784977246521	8.42906818923675	-17.57679248668445
H	-3.52795107897631	8.78973581116704	-18.43319300399428
H	-5.15618618545409	8.45714115463880	-17.84622254941689
C	-3.70618261808055	6.97177794491909	-17.29155672934824
C	-4.48517437028202	6.48673617902055	-16.15277470281181
C	-3.88219036438527	6.08234849580148	-18.52570898723317
H	-3.60993494440536	5.05371497432859	-18.29524360562746
H	-3.22809254294085	6.45213253514185	-19.31099167080272
H	-4.91112707407610	6.10809481835526	-18.87528288510041
C	-0.14357332684037	7.21950353734387	-15.61627539086041
C	0.63113989135592	8.75167104459615	-17.40846130452021
H	0.15047357637585	9.62025600576353	-16.96475993052883
H	0.64045215522349	8.87462138454409	-18.48877507629766
H	1.65499273611585	8.68626766181225	-17.04857117114465
C	-0.14344510658569	7.48648891323184	-17.05302350891085
C	0.35169808186898	6.26229920137954	-17.84385174579911
H	-0.38947603245868	5.46554366069092	-17.74291249995718
H	0.40971307834501	6.53226584683779	-18.89919795622172
C	1.71787808086494	5.76698067132600	-17.35439848735739
H	2.51929643761995	6.41217847155899	-17.71135594299056
H	1.73624191688969	5.74437934212522	-16.26129829043171
C	1.93624276735885	4.35076267868986	-17.84403335476500

O	2.91672523128115	4.26471829939741	-18.72926883112867
H	3.05855142733152	3.36540619255352	-19.04787424724942
O	-10.67315325492884	9.23799313911590	-2.33098544420988
O	-11.84883054515209	10.29841973524917	-3.90654563050900
H	-11.26316028799252	11.07989003527924	-3.72287229944330
O	-9.45405273654065	3.78646668256487	-4.74048869791217
N	-9.19668725657426	7.13723015037636	-4.04244871590168
N	-12.04123972920549	5.33249231996931	-4.10515293800035
N	-11.88427944041834	5.83262595254802	-5.23521021307286
N	-12.13032979383214	6.08142946021179	-8.58420524023357
C	-11.60147194976958	9.28110184304595	-3.10715254822327
C	-12.62637809129004	8.17752064805941	-3.25997136338671
H	-12.70718872948416	7.89429498918698	-4.31554014034230
H	-13.58717049492405	8.60522579352074	-2.97027946824221
C	-12.31296847759917	6.97024165460782	-2.38516525386119
H	-13.22549750456452	6.40627038546693	-2.19187741993273
H	-11.91281077726961	7.30684051064557	-1.42735712978767
C	-11.30438651550055	6.00035186534250	-3.02977280603620
C	-10.12868648527648	6.67810982386046	-3.55891052449328
C	-10.88523403445578	4.93458267366263	-2.01755822724159
H	-10.04363114966626	4.35156282534690	-2.39141552675573
H	-11.72889809698225	4.27389682463213	-1.83703164433426
H	-10.60036935522789	5.39522814614110	-1.07653470481458
C	-12.41971434577634	5.68678402575545	-7.54680199956839
C	-14.20198971364098	5.56614428487698	-5.87361921094568
H	-14.41282485071098	5.24862227250973	-4.85486606136784
H	-14.90459218823334	5.08884048450534	-6.55433748722937
H	-14.31837086286368	6.64605676457620	-5.93530270500599
C	-12.76584682315892	5.16975745239243	-6.22534133292619
C	-12.62084735418564	3.63721696494080	-6.19255810330367
H	-13.05325675494633	3.27747340210846	-5.25742985185256
H	-13.21701024833653	3.22777136854280	-7.01123591211333
C	-11.17639627895863	3.13677400390535	-6.30991665835777
H	-11.20254056036776	2.08489399816876	-6.61306914327167
H	-10.60852359875872	3.70146285221316	-7.05731927683023
C	-10.47258206778952	3.18211915998902	-4.97304036599772

O	-11.12590660076189	2.45735555269208	-4.07670988031061
H	-10.73440434585869	2.45210190380451	-3.18583500366645
O	-5.81625075411727	3.55569175034933	-19.10718407244264
O	-5.58773974798540	1.35018494823331	-19.33759064500038
H	-5.65385157536737	1.51723220567243	-20.28522426330204
O	-1.08382842232711	0.99345281012210	-11.91901348170148
N	-6.48571312240035	0.96037827034644	-13.90858248802996
N	-3.64025144265480	2.21437516600980	-14.97420911030730
N	-2.50242972175389	2.06595663691919	-14.49823713417655
N	-3.25567128343524	4.99424133584950	-12.99803526843822
C	-5.66199775067229	2.46223519366155	-18.62039159244445
C	-5.54682623123162	2.21399594730560	-17.13267230815050
H	-6.55789964614785	2.00916196589123	-16.75821847828456
H	-5.19373219053333	3.13517711318758	-16.65753476505629
C	-4.61598675132256	1.04271307801933	-16.81755425520540
H	-3.66910880564682	1.16937078398464	-17.34457356060709
H	-5.06402064769715	0.10637333512764	-17.14980698073822
C	-4.29713139002788	0.94021027832382	-15.31361567323991
C	-5.52912041224080	0.91223294316588	-14.54043938084830
C	-3.44002173622313	-0.29147024603609	-15.03005480683726
H	-2.49558721723680	-0.20133737407674	-15.56157893886038
H	-3.96170351085109	-1.18038525826671	-15.37447154930289
H	-3.23516864280999	-0.39588784584553	-13.96615477665031
C	-2.60249112867180	4.29232568445818	-13.62488929916517
C	-1.58119912995797	3.85650141535855	-15.81202643296649
H	-1.13458471556719	4.84680403472824	-15.79277716190810
H	-2.53765328062424	3.90229325386902	-16.33173939758262
H	-0.92084024722692	3.17975968594108	-16.34766448975573
C	-1.78297262786984	3.35377931375942	-14.38098434599199
C	-0.43318362616915	3.14992487664312	-13.66710234983639
H	0.27916583698793	3.87463354435344	-14.06405874182781
H	-0.53618772705192	3.33727437032211	-12.59264784774128
C	0.11594146597035	1.74132795276080	-13.87065065271286
H	0.05254177370825	1.42376189032651	-14.91176159366379
H	1.16920432324224	1.72069885711670	-13.58238455900300
C	-0.57870098205965	0.72827607813027	-12.98892093717417

O	-0.53603092258702	-0.48543184957422	-13.49905987742115
H	-0.90316156121642	-1.16854732172465	-12.87146395178040
O	-0.95547331768911	15.29805400481988	-10.07744112788495
O	-2.18665932217129	17.05161622081671	-10.65950953071387
H	-1.35908266945555	17.39572165281963	-11.02397826992268
O	-1.00178373607051	10.34915002847348	-14.79932546547657
N	-5.00178682242951	12.04356267501966	-10.82136309821437
N	-3.16334642301189	14.57480664380463	-12.17563963772765
N	-2.84160750291347	13.51347851354384	-12.73497777625786
N	0.08125556127144	12.04190156584005	-12.28296069637959
C	-2.02584714954725	15.87102173987338	-10.08097110486296
C	-3.28112302016455	15.36263568657233	-9.42354711832021
H	-3.43730921431697	15.99197480672062	-8.54333430841743
H	-3.13003731407025	14.33290758325806	-9.08937258678134
C	-4.49891714028184	15.49283410336279	-10.34192090610807
H	-4.53118034149530	16.50394184226153	-10.74973851553541
H	-5.41208508166762	15.34156831626654	-9.76413843444427
C	-4.47681056532352	14.50145840608494	-11.51594867884772
C	-4.76648049333057	13.12963492834941	-11.10082989272207
C	-5.48508665230700	14.92915049486892	-12.59036340502279
H	-6.48880637799401	14.96468548332576	-12.17251397743885
H	-5.46113517841128	14.23220154091313	-13.42658611775333
H	-5.21722616384855	15.91782352778838	-12.95312010811142
C	-0.64013384843775	12.74779064141394	-12.82958191396439
C	-1.03906877972960	15.05099536650823	-13.55229514778249
H	-0.73069744055256	15.37636947460757	-12.56079380369239
H	-1.81431142917341	15.72143743344827	-13.91533437250619
H	-0.18480770615357	15.09388472346907	-14.22298976732949
C	-1.58823716772220	13.62735545851441	-13.50552623177587
C	-1.94556843123680	13.09018047554821	-14.90840428252626
H	-2.67344843608719	12.28102137636887	-14.79571543002936
H	-2.42341433861156	13.89657102832627	-15.46492812370529
C	-0.73925612655997	12.56666727704876	-15.68801511334010
H	-1.04653442381515	12.37462292301354	-16.71788755135272
H	0.07753158138742	13.28712127483376	-15.70856603853539
C	-0.25404852463000	11.25124993438430	-15.11458920357303

O	1.05566327319891	11.19280088365930	-15.01409559943870
H	1.36518118118421	10.35178067154763	-14.54215866395736
O	-7.22341663277763	12.33332199786126	-6.96824145013385
O	-5.57047073754504	13.49574605282959	-7.90714132782694
H	-6.19424223904741	13.53472144738381	-8.70441979608863
O	0.59388241042440	6.79048244325446	-3.31261573768923
N	-4.50128518662385	10.41003060434719	-7.90555598780052
N	-3.74641676773314	10.89237714582468	-4.73028156714039
N	-2.77105344613353	10.59064679905887	-4.02147655325387
N	-5.15483753921314	8.35631418111426	-3.22295815868265
C	-6.16543557433780	12.91869637008990	-6.88160696362771
C	-5.42960086138825	13.12105858287551	-5.58072728119321
H	-5.78901582642789	12.41232622787335	-4.82956671523243
H	-5.71134522128543	14.12224643222562	-5.24724229437132
C	-3.91083632490489	13.07975856504213	-5.71305715406290
H	-3.47175186706329	13.46415738611585	-4.79298092241519
H	-3.58909472159064	13.72218147182231	-6.53468908256828
C	-3.35381054352528	11.66202822452617	-5.93123905877441
C	-4.00303375784154	10.99936452271748	-7.05711931152473
C	-1.84092539397931	11.70280880369815	-6.14604226206936
H	-1.36908032398539	12.16478048984166	-5.28170338184664
H	-1.61392449455125	12.29604773510036	-7.02928441351674
H	-1.44286353908181	10.69499182057102	-6.26669798989427
C	-4.27000320066651	9.04550134909974	-2.98119391853686
C	-3.56266886504901	11.07437233371045	-1.80059461416699
H	-3.85727881630775	10.66115494389331	-0.83877590075913
H	-4.39449852198906	11.63228973392186	-2.22839219366624
H	-2.72287696866849	11.74944660917153	-1.65433952245131
C	-3.14790842116885	9.94698607714336	-2.75224236921559
C	-1.91138717343410	9.21003584918457	-2.21499696604674
H	-1.17007649691262	9.96979526015183	-1.96219786750293
H	-2.17163786946640	8.67561392583789	-1.29879555265943
C	-1.32233263134672	8.24114467288402	-3.24333732220459
H	-1.93188269629752	7.33595438146223	-3.34028450423835
H	-1.28809466319442	8.72897659082999	-4.22627125192832
C	0.10172353168401	7.83583465158152	-2.94742270566393

O	0.79602840313021	8.75001040067686	-2.28736352227123
H	1.70448681565646	8.46946542662924	-2.12048345587324
O	-12.02866423946157	11.99219490391815	-1.20995412131024
O	-12.26581980309616	11.69631306626879	0.98213233581346
H	-13.17889892734102	11.98051056944573	0.84948124849223
O	-2.61494554006382	6.82858936632766	0.45422625231592
N	-6.55459616222377	11.03694685540824	-3.18550826288188
N	-8.11576125664039	9.51661901241175	-0.63316895084897
N	-7.15485580734305	8.77490834781495	-0.88906344619678
N	-7.05548376230764	5.83755885763134	-2.49224895144734
C	-11.55164718703870	11.71082927992241	-0.12962966460353
C	-10.12170364848406	11.31390603281635	0.12427447159022
H	-10.12852441070401	10.22809085059873	0.27324745605670
H	-9.80233332470520	11.76696017440251	1.06300766227803
C	-9.19003796817191	11.67322264543478	-1.03210092448755
H	-9.00529078323558	12.74873707455568	-1.04803888431351
H	-9.67315923966211	11.39698829703033	-1.97323354094118
C	-7.84841919206851	10.93189668358677	-0.92881786586028
C	-7.12267930423070	11.03018767389226	-2.18937654954302
C	-6.97023150619575	11.43816452668119	0.21973560280919
H	-7.50763732206103	11.34945663563567	1.15985872409306
H	-6.70084157775555	12.47854269558760	0.05754527026342
H	-6.06497953436580	10.83483178028547	0.27613656897524
C	-7.20480417481689	6.52011216274277	-1.58414244040461
C	-8.75147226687784	7.18339043658970	0.20053482812541
H	-9.53671791573656	7.45478927899230	-0.50528422333434
H	-8.84612320973167	7.81625200406360	1.07920905096476
H	-8.86713998782740	6.14397783063574	0.49818654878238
C	-7.37492912165466	7.39090895082875	-0.42672429347866
C	-6.24548607062858	7.10973747194751	0.58295019189185
H	-6.41939795188690	7.71851338670718	1.47069540527028
H	-6.28527302889314	6.05974805758447	0.87832824862239
C	-4.87881054909333	7.44558685166607	-0.01369719347553
H	-4.73408442815354	6.96515838456949	-0.98751427384269
H	-4.80611057762794	8.52687726330918	-0.17026064937501
C	-3.72238963913104	7.05777758111685	0.87693719201097

O	-4.04124954700263	7.00995070435974	2.16211988901339
H	-3.28962478042860	6.79512444199464	2.72864351966021
O	-4.86342027311033	14.09596558819774	0.34093608895395
O	-4.79718492681730	16.12964959876300	1.25740478915749
H	-3.92773793143271	15.86631222216017	1.59237226386912
O	-1.93889388175003	17.30472790883172	-7.12778574575267
N	-7.09022038810625	14.01925709771607	-3.10507985651254
N	-4.63818455395732	16.11783351046714	-1.92532901972972
N	-4.00998414614712	15.49617231390609	-2.79829675900751
N	-1.52991900082448	13.23258224123019	-3.16304762552137
C	-5.39033969786834	15.15569275823081	0.57510704046878
C	-6.78818885411647	15.53520297067247	0.14879209225874
H	-7.32335510159050	15.89040377547246	1.03010666603053
H	-7.29653190801787	14.65289230961539	-0.24387310594822
C	-6.77289109594417	16.65857117041722	-0.89513275778835
H	-6.24605751506925	17.52526016708731	-0.49197063143870
H	-7.80008865155646	16.94966468058130	-1.11604736934759
C	-6.07738608293853	16.24292344454872	-2.20075578180741
C	-6.63451562639978	14.99500994033387	-2.70806074824533
C	-6.20981428359308	17.33976581587278	-3.26583439076464
H	-5.67813281599750	17.03694328120723	-4.16654376708464
H	-5.77129094267765	18.25902491217647	-2.88574284520413
H	-7.25781572010390	17.50990092674639	-3.49888486140022
C	-1.98589839331163	14.25465036006926	-2.90750183460920
C	-2.19962585151939	15.87503500514942	-1.10278331787651
H	-2.64792499117664	15.13672750539827	-0.43994134788346
H	-2.58227859987590	16.85867722008124	-0.84328498460525
H	-1.11970280705662	15.86571100832444	-0.97777695735185
C	-2.55120332522276	15.55278513035354	-2.55658778169108
C	-2.01973723308237	16.64619380190000	-3.50693388177072
H	-2.48311836498042	17.59016075918282	-3.21528413109216
H	-0.94118423245519	16.73904257070942	-3.36997572283760
C	-2.34232154450189	16.34635514816558	-4.97013057207636
H	-1.90084366534898	15.39610180227213	-5.28510711808839
H	-3.42433418823220	16.26429243336416	-5.10907040552748
C	-1.85255930900632	17.40900056001340	-5.92913245101272

O	-1.32373309171743	18.46459131426292	-5.32616053537684
H	-1.02878732882636	19.13742191960212	-5.95360149026927
O	-10.63081367966785	1.98877404308739	-1.23453591394287
O	-9.79935370416659	1.76655880818600	0.81674665306748
H	-8.91067594838970	2.04197912614976	0.40172083393125
O	-18.85804053730188	-1.74280824755815	-3.86000762675897
N	-12.20431137715383	0.18289396067260	-3.07824753827348
N	-14.94229804693380	1.72479050520263	-1.98795895032862
N	-15.38727722481945	1.09387733398737	-2.96005033341367
N	-15.49991144299594	2.53260805624815	-5.93975602336025
C	-10.77699876672058	1.76116368542643	-0.04084030426725
C	-12.11623193676062	1.44586009258956	0.57714733410083
H	-12.13134480846633	1.88029038127361	1.57702628759362
H	-12.16534693273258	0.36235973074273	0.70461781905131
C	-13.30036487920619	1.96877472370357	-0.24174226775746
H	-13.00777236880048	2.87908326340309	-0.77218806754591
H	-14.11242102097092	2.23160843509739	0.43706787226195
C	-13.88635212056158	0.97947200400414	-1.26290088788092
C	-12.91204150629931	0.52435257713730	-2.24285793885377
C	-14.56417746552721	-0.22568496723041	-0.60285542781851
H	-13.84136996315705	-0.82165491868033	-0.05221027721785
H	-15.02701516728971	-0.83986908671067	-1.37304739826258
H	-15.33246146114012	0.12805071762248	0.07998446097440
C	-15.94561801960279	2.21131299642757	-4.93396966034662
C	-16.98236109546314	3.02678125353992	-2.87491275801572
H	-17.84586425684906	3.45280705686078	-3.38016926177124
H	-16.19416462710713	3.77571175598357	-2.81446645962527
H	-17.26125235179998	2.73846998756362	-1.86471271044721
C	-16.49188860193237	1.79881687954011	-3.64245854596165
C	-17.59179569295129	0.74820192479328	-3.88337159325334
H	-18.41186163638567	1.20932813392586	-4.43662119578483
H	-17.17624831637501	-0.05683515744090	-4.49720951410657
C	-18.11479340916128	0.15599960425705	-2.57434613148381
H	-17.28341166871877	-0.08928352366097	-1.90752914695495
H	-18.76516836905128	0.85048012339025	-2.04189959154047
C	-18.87549558751838	-1.12676882799861	-2.82518040826259

O	-19.55992149890674	-1.51323240872305	-1.75518233564887
H	-20.04676422995325	-2.33335384299762	-1.90510201521747
O	-15.05130166622834	12.59709859234450	-13.34424884272666
O	-14.54945851019206	14.77149729250275	-13.38713793227538
H	-14.66129389606309	14.69781272402555	-14.37458688344531
O	-13.47555580508887	8.97155540458093	-6.30350123696070
N	-12.63312697170749	11.30886357536226	-9.34637340669696
N	-15.94048083772731	11.49201037455799	-8.54315451934771
N	-15.54876045627826	10.31307243141240	-8.50369601354990
N	-15.64825896338225	7.03710515828595	-8.20313362801167
C	-14.77365638087913	13.62957674881563	-12.77811642340896
C	-14.60146206319263	13.74613868234603	-11.27442299480689
H	-14.77748161922766	14.77779669372653	-10.97271199003198
H	-13.54840441755352	13.51684137517433	-11.07743537554656
C	-15.51289474614378	12.77556926627796	-10.52281011352180
H	-15.61318259136664	11.84471219728254	-11.08957631615037
H	-16.50867014877677	13.20570910633360	-10.41035788589582
C	-14.97489998918784	12.43053896106671	-9.12057201240480
C	-13.65439304624598	11.81885339797465	-9.23871692401942
C	-14.94156655392508	13.63847162676117	-8.18380759336935
H	-14.58538890417886	13.33285594343141	-7.20105437393242
H	-15.94740185888568	14.03820263212133	-8.08486812115788
H	-14.28912885839234	14.41132613508954	-8.58045300224538
C	-16.10016632236110	8.08103849805929	-8.05344559233860
C	-17.88539017271426	9.61619154577803	-8.72166736898913
H	-18.64542442783864	8.92648886995894	-8.36537523110052
H	-17.69255364781695	9.43243771732931	-9.77745631943997
H	-18.23803481251964	10.63738236723700	-8.60252512756635
C	-16.59371586174437	9.44449521267856	-7.91715263763044
C	-16.80495449972727	9.84620243492010	-6.44546769895306
H	-16.85554610779289	10.93671451987704	-6.41718989752674
H	-17.76093148303229	9.44875115627903	-6.10554247721801
C	-15.70531510087550	9.34755767642539	-5.50297120957165
H	-15.88500197434211	9.77790577039229	-4.51713536824938
H	-15.73931238407800	8.26018702786414	-5.42063675194644
C	-14.32091618233174	9.76599150641029	-5.94886807447865

O	-14.13688443462066	11.06387349278844	-5.89288148467993
H	-13.17779055877766	11.32328341585364	-6.12215235345485
O	-5.82259097836946	13.62524531654326	-15.75728880069245
O	-6.17331738091121	13.33913206004462	-17.93889630691688
H	-5.69980532957620	12.44284070934077	-17.81927214600738
O	-14.70308849636083	14.65863283766219	-16.07535443878914
N	-8.13138883413375	13.33803836608366	-13.57964033179106
N	-9.37236038554568	14.58679298836694	-16.55666638949219
N	-9.69261173003271	13.38816225614699	-16.45986780836968
N	-11.06176702099327	10.43851007465140	-17.48151503394406
C	-6.22715132199325	14.03203187854542	-16.83661697656104
C	-6.81144303083980	15.40673920823253	-17.02687260074295
H	-7.49215274396583	15.39274765607007	-17.87846693386757
H	-5.97487424349586	16.06018771648687	-17.28575286662557
C	-7.50253518240150	15.96364203632225	-15.78365764476886
H	-7.82118519364600	16.98199008833241	-16.00455305191846
H	-6.79920763964656	16.00461273548961	-14.94987119216485
C	-8.74066876352344	15.14429072335003	-15.35808098416232
C	-8.38363646811558	14.11211758396090	-14.38768301471849
C	-9.79981168083073	16.06255652060247	-14.73095959360361
H	-10.65559515508823	15.47689806080880	-14.39911198127260
H	-10.13055664197713	16.78061073238669	-15.47615236781384
H	-9.37387104428062	16.59553228226463	-13.88506783798890
C	-10.75332486642571	11.54305547290581	-17.54781624390407
C	-9.41082908280917	13.07945103307144	-18.88127242693533
H	-9.88923257391217	12.72895469593876	-19.79213124531844
H	-8.50436123681185	12.50391281839583	-18.70006777057251
H	-9.14705363642557	14.12798534613828	-18.99509502116249
C	-10.37379465601045	12.94182160106168	-17.69605021956348
C	-11.63454922577871	13.79838632715188	-17.90910463144840
H	-11.31064322810043	14.82722491974475	-18.07411689575816
H	-12.14995169214734	13.45169057912781	-18.80578366309529
C	-12.57466329854251	13.74834770055055	-16.70550597066435
H	-12.85001422427020	12.71778490997335	-16.45730878758583
H	-12.08991568817858	14.16675863037754	-15.81880967016159
C	-13.85647369670930	14.51044187609219	-16.93010854542299

O	-13.99297735588645	14.99972804071382	-18.14912419825920
H	-14.82403268892289	15.48082855541225	-18.25617725964044
O	-1.68600199123750	8.20507875308486	-6.49033934011587
O	-2.44658756162454	6.34288367600579	-7.43405405925072
H	-1.47789610925536	6.24759782877226	-7.69954234837079
O	-9.30995038341591	7.30515338023895	-8.94267358806079
N	-6.02681500910385	5.79156004182530	-9.04853425031984
N	-6.80677528353213	7.73397645160129	-6.55874284402781
N	-8.04766991089036	7.69473665854064	-6.53325498985090
N	-7.54834868793175	9.95981639699171	-8.76877762144248
C	-2.60957243016560	7.44365213136321	-6.74831792098930
C	-4.03254698319961	7.74354353333589	-6.35854106921719
H	-4.50564735108635	8.14708447352307	-7.26479987076827
H	-4.02198480926144	8.54998740771964	-5.62318865224870
C	-4.81930337371183	6.53417159703421	-5.83814710182666
H	-4.98509989817386	6.65689034849807	-4.76558071724611
H	-4.24896258384853	5.61270369870244	-5.97305382452472
C	-6.18813877033200	6.38951023957137	-6.51606805002820
C	-6.05596985031431	6.03591892743640	-7.92840704110127
C	-7.06201252760522	5.35815252771287	-5.80368463242075
H	-7.98761772428614	5.17969181448854	-6.35437681313663
H	-7.31354596417072	5.72428595088055	-4.81014381646824
H	-6.51922687896504	4.41847009326082	-5.70498919162221
C	-8.08815737409298	9.63790466481997	-7.80780259883629
C	-8.23289190377488	9.83828786730309	-5.37672803219624
H	-8.62671031079735	10.85026891232159	-5.43312254127840
H	-7.14406972777733	9.88056516547930	-5.32474591193388
H	-8.61622335582512	9.35863547865790	-4.47717023743939
C	-8.65762854683770	9.03804844627468	-6.60788874757221
C	-10.18702771107610	8.85444044709451	-6.67589430005843
H	-10.42674927928074	7.86565732451925	-6.26591559566753
H	-10.66362872788875	9.59563046978721	-6.03363160816221
C	-10.78828691235057	9.00610095246960	-8.06792231175367
H	-11.85134090730097	8.75463908898420	-8.03139587660813
H	-10.73124570063309	10.04588310859504	-8.39951448270686
C	-10.15157055029412	8.15262459990287	-9.13578245116089

O	-10.61013291497797	8.46851915497442	-10.33255264272984
H	-10.07589953737677	8.00934508955495	-11.06276432430322
O	-6.26791333347875	9.11639682522420	-11.81911923000624
O	-7.69702504561823	10.73581460286078	-11.24421498510710
H	-7.49351651059456	10.50444508075466	-10.26940403344146
O	-12.52114956160528	4.09735157411952	-17.39397336218574
N	-8.93922891863728	7.44129812626403	-12.05200862326292
N	-8.01998795317969	7.05586393359171	-15.17088713328686
N	-8.93252060425586	6.53128620459737	-15.82984354355574
N	-6.95977402731940	3.95364960082711	-15.05994547830271
C	-7.04129424641929	10.00158172014183	-12.11382283057233
C	-7.35506600048939	10.40110011646223	-13.53871898268703
H	-6.62625122939423	11.16756438628220	-13.81197909657703
H	-8.33712858448833	10.87312930202446	-13.57597688746948
C	-7.24852949542215	9.23884571513087	-14.51965269316467
H	-6.33118434990860	8.66872119056609	-14.32560602631974
H	-7.18986552781484	9.62644285277834	-15.53683395908508
C	-8.45536870456376	8.28329456397690	-14.46335628746833
C	-8.73093431400066	7.84863746702539	-13.10432271744409
C	-9.70821870906771	8.88223523637060	-15.09601537575339
H	-9.51720267228074	9.04354672341848	-16.15371847027632
H	-9.96366763496470	9.83188559039544	-14.63042943529745
H	-10.55417470524154	8.20268848360580	-14.99519722741659
C	-7.60927636470016	4.55562837955387	-15.78804760089944
C	-7.62255358358888	6.01842556764533	-17.76053306041651
H	-8.21925587160839	6.75258131226811	-18.29496156896408
H	-7.29152395941973	5.25209508012226	-18.45542041559638
H	-6.75940539961952	6.52551383690434	-17.33163941800817
C	-8.45094832502191	5.39803731212272	-16.63337505086740
C	-9.65836957959614	4.60542133894150	-17.14588498651525
H	-9.30468448298834	3.79180522147699	-17.78190465378411
H	-10.18215528486742	4.16113396934578	-16.29269293472598
C	-10.62951333914998	5.49288441990899	-17.92959529222510
H	-10.87672015582388	6.38161512737692	-17.33825741873213
H	-10.19928727097600	5.82247716879649	-18.87427472694278
C	-11.91997004921032	4.75416042699778	-18.20869563682600

O	-12.32695425136134	4.91359931857138	-19.45893798480338
H	-13.13526863735397	4.42881669860305	-19.66312458240763
O	-18.45071959029196	3.73626849260120	-11.56672144203002
O	-20.40124353440260	4.07687618755324	-12.59763955555564
H	-20.43542752934576	3.12196155645358	-12.73274991224344
O	-12.48549517692949	10.94207164730377	-14.91907911407190
N	-16.19996513214284	4.69862418937263	-13.61942960559163
N	-16.05516563444893	7.68921444912929	-12.08374667082419
N	-14.98412127960435	7.43774458925187	-12.66336642355509
N	-11.68886533764866	7.36805306356777	-13.12819305831280
C	-19.33437820621125	4.47762167379576	-11.91779909792198
C	-19.40861175812945	5.95351849739333	-11.60953701495539
H	-20.03339654877846	6.03326897644245	-10.71588549986425
H	-19.94230554401690	6.46378471464871	-12.41060380238402
C	-18.05502691587195	6.59397929724871	-11.31200566741995
H	-17.49976976275949	5.96133058538738	-10.61290820144501
H	-18.22445315530909	7.55839502852062	-10.83180428423786
C	-17.17861963311243	6.86245881540743	-12.54740790746390
C	-16.67353751079018	5.63500630620918	-13.15510538784579
C	-17.88050695684530	7.72807568381897	-13.60067594851611
H	-18.68880981887647	7.17770405076014	-14.07348582527379
H	-17.15972567355025	8.02364948005990	-14.36091736661049
H	-18.28026541248967	8.61998640668141	-13.12548128737502
C	-12.66728500191641	7.86262411880183	-12.79110026921025
C	-13.81107138212215	8.51701987885978	-10.74666982084166
H	-14.75545325544384	8.88643924495092	-10.35380526937604
H	-13.02350593452910	9.22617990914641	-10.49175146899410
H	-13.58805180268182	7.54769201644182	-10.29379346978496
C	-13.91972936877602	8.39690708765888	-12.27068159145482
C	-14.30471129769991	9.74970987069691	-12.88899434320557
H	-15.25986452966655	10.03329102285498	-12.44448692541148
H	-13.56882483084901	10.51057984355420	-12.62076248892684
C	-14.47612397541281	9.69706228164946	-14.40977851959295
H	-14.90400138251513	8.74735563886169	-14.73461612448315
H	-15.16660997937840	10.49533732362029	-14.69427687684531
C	-13.21491833422125	9.99901329470614	-15.17396650228677

O	-12.99000075872872	9.19385033983138	-16.18676961717956
H	-12.24854515678512	9.57979271804009	-16.75578736656439
O	-14.64607240346646	3.82059279603322	-8.76217926654333
O	-15.19429834354044	5.41410470344169	-10.22331932273670
H	-15.32927571202599	5.98529002085180	-9.40573383264157
O	-9.21545760154521	1.92908289437936	-15.57646747775837
N	-11.66049696191751	2.86359344909754	-9.28663218827861
N	-12.84973890236566	1.89413077249576	-12.25789672877870
N	-12.28352749340781	2.99954979903439	-12.34836244041846
N	-9.65834383825879	4.57904407918027	-13.54840693267241
C	-14.95101093741833	4.16057650620655	-9.88235446310688
C	-15.14391064941420	3.21236823292547	-11.03810017943563
H	-14.63414736239945	3.60028981082771	-11.92403459797077
H	-16.21413109429353	3.23412422707924	-11.25887091268552
C	-14.72674882312191	1.78153459171836	-10.72729142500078
H	-15.24152980135935	1.10548897475201	-11.41031264850547
H	-15.01959064350956	1.52160139366928	-9.70717355143264
C	-13.21771807484167	1.52516716545218	-10.88617695546230
C	-12.40096512835961	2.28278334049846	-9.94211441007442
C	-12.93696822336957	0.02689646093141	-10.74220423012002
H	-13.47271256418189	-0.50855730174840	-11.52271942660287
H	-13.27089682233646	-0.33068052501311	-9.77115515501375
H	-11.87252895668959	-0.17707603246583	-10.85191614264640
C	-10.64966395054930	4.01762572527535	-13.67176217825231
C	-13.00271925478090	4.38944592996770	-14.17235289761099
H	-12.74234470577079	4.80146508206142	-15.14458857192677
H	-13.06930525011893	5.20054286863387	-13.44588179987317
H	-13.97303397924333	3.90287725743318	-14.24699018843591
C	-11.95185494880279	3.35652655273603	-13.74069867152141
C	-11.96209352942624	2.15471439468362	-14.69640107106824
H	-12.98659610919622	1.78120759926252	-14.73729366958057
H	-11.69639888446293	2.50347863285579	-15.69655656272803
C	-11.04777728220771	0.99948842157122	-14.29073788120476
H	-10.96583882523161	0.90193364929620	-13.20350069704337
H	-11.48011611897591	0.06241048337192	-14.64555126847805
C	-9.65973900043370	1.05965217237528	-14.86860480123366

O	-8.95746103015275	-0.02410832727694	-14.52753266331762
H	-8.08077825911364	-0.01905770614482	-14.95362685723140
O	-4.67836625258296	3.74643615973464	-7.21190728625863
O	-2.92840649636608	2.38822465156194	-7.47771445152501
H	-3.23726162213622	1.92097135575922	-6.64546567875601
O	-8.97618131961091	1.87483396591592	-11.91013064811345
N	-5.93205223050491	3.90346839852444	-11.92189627252059
N	-5.23064579512440	1.84394756860924	-9.38998079547959
N	-6.44903979615647	1.73648394514644	-9.61760853917896
N	-8.56080008052233	3.58522858128879	-8.05885676590304
C	-3.72829783905438	3.36290085180182	-7.86062010183394
C	-3.30733449311833	3.94537833163038	-9.19024872142479
H	-2.33851063161342	4.42792481992427	-9.04511718237397
H	-4.02540758388157	4.70714683631202	-9.51627401285188
C	-3.13846703400349	2.83209229409885	-10.22315908827254
H	-2.47717285736412	2.06069382970069	-9.82129193456389
H	-2.66542791415257	3.22665712131236	-11.12537078840727
C	-4.47389812671967	2.18837361043487	-10.60993908315154
C	-5.27628181845684	3.12680321892688	-11.39214624765810
C	-4.23368350926588	0.88958684057281	-11.37273113615921
H	-3.73219293585783	1.09888960872757	-12.31505626493713
H	-5.18102816936366	0.38971407022046	-11.56260160322576
H	-3.60378125068198	0.22651080162853	-10.78321136371079
C	-8.02211445839165	2.57993055098011	-8.17800633587066
C	-6.42305949858455	0.99144480718511	-7.20798560357203
H	-5.87655886217801	1.84824050242866	-6.81555036287626
H	-5.71282802383374	0.20814958045216	-7.46658338158537
H	-7.08947760909380	0.61630434997854	-6.43355173695912
C	-7.24778791775537	1.36729263563418	-8.43590722244620
C	-8.15364762999401	0.19931347854578	-8.85484826097005
H	-7.52150802623423	-0.60880038679791	-9.22947085570810
H	-8.67678768543704	-0.16967640858359	-7.97116999223996
C	-9.19203290076894	0.57325134648390	-9.90907217965320
H	-9.81916192392076	-0.30510636063066	-10.07573474781596
H	-9.83673170594935	1.38779703286723	-9.56674449626143
C	-8.61348235007843	0.92219310259656	-11.25848388004269

O	-7.71470258717310	0.04722933858715	-11.67581540999282
H	-7.40493626021143	0.25068171727894	-12.60261068701578

RS-RS

```
:::::::::::::::::::::::::::::::::::::::::::::::::::::
::                  THERMODYNAMICS                  ::
:::::::::::::::::::::::::::::::::::::::::::::::::::::
:: total free energy       -1371.546636290120 Eh   ::
::.................................................::
:: total energy            -1376.821436260697 Eh   ::
:: zero point energy           5.797001855756 Eh   ::
:: G(RRHO) w/o ZPVE           -0.522201885180 Eh   ::
:: G(RRHO) contrib.            5.274799970576 Eh   ::
:::::::::::::::::::::::::::::::::::::::::::::::::::::
```

O	1.09290925887009	0.12612138743251	1.47703408137714
H	0.36468410659911	-0.20978423200724	2.07846867771496
O	1.46802917179457	-2.03684491613608	1.11103497126565
N	3.51964402993590	-0.85004046354121	3.77354440298293
N	5.61120386404837	-0.66341627905030	1.14047093470803
C	1.74498441555515	-0.86659375069958	0.92284817463956
C	2.85353673424716	-0.42506853223476	0.00292789120043
H	2.37822908148480	-0.06928023797325	-0.91229339584726
H	3.46991034166952	-1.28823838295654	-0.25446769401204
C	3.69797840832139	0.71150735285233	0.57638732835801
H	3.04688452258406	1.50711964835409	0.94240924726292
H	4.32146491716854	1.11964634423401	-0.21940288306283
C	4.64097202096157	0.28390102715228	1.71849715850605
C	3.95481104965806	-0.34972684317385	2.83849520902181
C	5.41987349105409	1.49651683612860	2.23357469670473
H	5.97017316919441	1.93506430932822	1.40539563911629
H	6.12733461150046	1.19603731055624	3.00809770377756
H	4.73867885253382	2.24198339836704	2.63949893333340
O	4.34411618413052	-6.89054310086214	-0.69211179073485
O	4.66567394648116	-5.73992641975399	-2.57925162270812

H	4.03919362254561	-6.34795141729939	-2.99257855348873
N	7.08991699381639	-3.90441578997219	2.53286201508947
N	5.15916007768489	-1.81805697076307	1.05720723865189
C	4.89734295357869	-6.00893607221627	-1.30350913306385
C	5.91186688969983	-5.06119668779082	-0.70281639387286
H	6.29943965258505	-5.49683773258063	0.22049495060516
H	6.73175451895700	-4.92477455211261	-1.40779575924395
C	5.22710203070568	-3.71682037818594	-0.42362353340448
H	4.98944264412321	-3.22749711974426	-1.36911655633965
H	4.28679659940769	-3.88089108133715	0.10800972352976
C	6.09738370209446	-2.76260320537815	0.41413205728622
C	6.68919207370172	-3.44076641057044	1.56405307707487
C	7.15876260050970	-2.06760266387820	-0.43393465227303
H	6.66518367969175	-1.43857530870350	-1.17067490501201
H	7.80321195666218	-1.44318940735198	0.17900549292654
H	7.76494769210269	-2.81139280710154	-0.94410552708153
O	-0.09200767223482	-2.96109400158566	3.05527533867968
H	0.26291077201770	-2.62875840835639	2.18272213862868
O	-0.40712129288679	-0.79702680200304	3.46425810102039
N	-1.16247547747766	-3.74978153016582	8.35378207343723
N	-2.94171268877322	-1.16269258903204	7.11382569565275
C	-0.44767495956095	-1.96717165102273	3.82381227422464
C	-0.87904791775178	-2.36979480805623	5.20856456953289
H	-0.62932466714665	-1.53865050861174	5.87684492787580
H	-0.32500971601014	-3.26791077729095	5.50263815030392
C	-2.38400715034639	-2.64680125348226	5.29720524604048
H	-2.61314352464333	-3.63104383176971	4.88448179917263
H	-2.94524145769357	-1.89847757237369	4.73545862936840
C	-2.86733852075192	-2.58793805477179	6.76467548003856
C	-1.93216351008169	-3.27013632144809	7.65193637071197
C	-4.27782055315808	-3.15955224895015	6.89960010187083
H	-4.28892262369164	-4.21427089767424	6.63394573623872
H	-4.94441474519604	-2.61761794657129	6.23371558414409
H	-4.63282763275554	-3.04084160021803	7.92158617695385
O	-3.53912506122445	0.30209668094954	4.21452678405488
O	-2.33582824228346	1.85664057849786	3.16872713691055

H	-2.92341177734194	1.66951955753515	2.42464324449067
N	0.27894813504103	1.70336255546358	8.50112014276552
N	-1.89255139481432	-0.69243495455914	7.58898827251508
C	-2.66503599334390	1.13529338187134	4.23203796849112
C	-1.83035520809344	1.45643721172573	5.45137221239064
H	-1.08059640318333	0.66095348137354	5.52478886743389
H	-1.30695100221542	2.40252670299872	5.30706203601915
C	-2.70172760806722	1.48286137152706	6.71509651747014
H	-2.89517494904931	2.51467645467748	7.00849523723256
H	-3.66156324712622	1.00157841271503	6.51163504772137
C	-2.06550294140119	0.74171740898682	7.89975813687431
C	-0.75276902144859	1.28942894469921	8.21784063108054
C	-2.96339229723283	0.82772686874146	9.13880356881971
H	-2.50268369897941	0.31215405643688	9.98126288578718
H	-3.13048165232770	1.86804034760911	9.40409251856833
H	-3.91490224815933	0.35155686042596	8.91200305051573
O	11.59523300958207	-2.74147734622219	5.28689417620442
H	12.20207200454640	-2.07519480896822	5.71385641178943
O	10.52378495054724	-3.10325450808162	3.38762947034426
N	8.13516399402873	-2.04307555492954	5.34625607839246
N	8.39559804243924	1.07143986169253	4.18156647038082
C	11.21074265567911	-2.38158639762499	4.07598202203051
C	11.61997224724024	-0.99639194605603	3.62755809145242
H	11.59056967868367	-0.95805006369520	2.53940873841280
H	12.63886911176873	-0.78904417259140	3.95275096052867
C	10.69860862955458	0.07232520635289	4.22978899854401
H	10.98183511937969	1.04008849551454	3.81242429532432
H	10.85916887214966	0.12208212435753	5.31015345053920
C	9.20569373939260	-0.13929435947652	3.92432709438834
C	8.62247334091928	-1.21122518073237	4.72662893328076
C	8.94336292114542	-0.42414498834279	2.43750232262825
H	9.42510729129805	0.34396885239830	1.83860483168546
H	7.87161737758836	-0.39567468183683	2.24102330418755
H	9.33342614080957	-1.39935252296145	2.15433296791293
O	10.97499030264387	4.80002679981772	7.93770753970922
O	10.57653466906719	2.70066266090247	7.32779982972665

H	11.46823044884546	2.54716412227231	7.75243280881027
N	5.89748232186260	2.70553666095456	5.62537747941066
N	9.02312367096137	2.01904877347830	4.68764495035940
C	10.23186037724147	3.96107234660177	7.45049692310886
C	8.87004465256231	4.30873002390250	6.92442948747510
H	8.14735569861561	3.55624732658315	7.25135735343234
H	8.58847911816558	5.27436223697955	7.34541559169240
C	8.91762435463849	4.41209296970518	5.38910733613490
H	8.42205459290573	5.33692811116208	5.09155173676796
H	9.95178808383879	4.46866373078165	5.04519042780883
C	8.23361442455329	3.26985368084757	4.62886313548967
C	6.90710615186299	2.99290705807733	5.16189283436831
C	8.17183357577642	3.65253465939697	3.14372685709736
H	7.52790961896795	4.51835634413946	3.01127553601347
H	9.17669326164518	3.89218212866916	2.80246622650518
H	7.79472500996047	2.81717889554538	2.55893272214600
O	-5.04393377332208	-11.99799805057448	9.99662441513793
H	-5.29286082851360	-11.08639242626980	9.63061679915009
O	-3.07560000632270	-11.10358653758449	10.53985527146262
N	-0.47011286435382	-12.32973119257057	14.70449160723387
N	-3.45797975396856	-11.71044372579781	13.47676727460434
C	-3.85241576947576	-12.04460871941171	10.52756131596884
C	-3.51680597280798	-13.40026653292404	11.09712852163667
H	-3.28784583342208	-14.03877040609557	10.23945932577560
H	-4.40855469537492	-13.81553595218540	11.56604517577593
C	-2.31869101910945	-13.37653869997773	12.04628522842676
H	-1.56719956489782	-12.68250576063441	11.65278276028825
H	-1.87261713174229	-14.37102579141364	12.08048177448615
C	-2.68452520126189	-12.97574820326929	13.48977750857041
C	-1.44728971704735	-12.65229130879764	14.19586383721684
C	-3.45587947412118	-14.06770920587925	14.23177750999594
H	-3.64034631174137	-13.75558463022735	15.25739175805827
H	-2.88774537774688	-14.99392085428823	14.23096156563835
H	-4.41215367161162	-14.23310740671767	13.74276225462081
O	-7.93023741492933	-11.34552180466452	11.05866029044515
O	-8.49438624435806	-11.53421556449502	13.20635530892450

H	-9.37137866639888	-11.75950135827836	12.87105546809361
N	-3.52994906777364	-8.52470020402087	13.05899342217552
N	-3.96286836530521	-11.47006217213727	14.58929205394941
C	-7.63340174591309	-11.29224144414773	12.22373731469640
C	-6.23674079656345	-10.96522188248104	12.70655348267691
H	-5.63995326405922	-11.87173086523859	12.56304477658443
H	-5.82626543894169	-10.19700221984225	12.04464744743822
C	-6.17354664679596	-10.53826356419799	14.16878937290258
H	-6.78788389954010	-9.65105504694322	14.32858881739156
H	-6.56080386374371	-11.33687873641859	14.80363486212651
C	-4.73659259107077	-10.21991463257059	14.62520330035728
C	-4.09898905822574	-9.24685369559255	13.74382311162073
C	-4.74636816889024	-9.71672577444635	16.07093276674154
H	-3.72773656995856	-9.61464835093185	16.44283052026003
H	-5.24546166054905	-8.75136915097766	16.12573710659560
H	-5.27876346560597	-10.43090367289259	16.69430024572449
O	-3.62319254949374	-8.74875973024340	9.62479399522614
H	-3.45333345947442	-9.69662142344760	9.96917660485406
O	-5.58984239598171	-9.59370614246345	9.02800645075513
N	-2.50435026501026	-6.16995056737240	6.16187451592872
N	-4.22179008138096	-9.02618195418617	6.45546022922039
C	-4.81603979591107	-8.65345006063576	9.10287730724919
C	-5.17339315343324	-7.26296762561320	8.62865008936371
H	-4.31089027310327	-6.59919235509679	8.72226426861932
H	-5.96213148219066	-6.90157959582387	9.29098205437629
C	-5.71399922391714	-7.26338000782710	7.19676607807124
H	-6.10369398597847	-6.27487831888337	6.95536369985258
H	-6.52969406096134	-7.98466310908475	7.12167931744718
C	-4.64736080753919	-7.65099887807672	6.16454224564592
C	-3.48178303904757	-6.77541534270233	6.18022682321693
C	-5.23375251883294	-7.68481318442197	4.74726212445698
H	-5.61057493545233	-6.70249731328813	4.47628787505809
H	-6.04808777808135	-8.40371580100375	4.71833021692045
H	-4.46297125386048	-7.99130598606909	4.04294275390716
O	-1.51385297565484	-13.25097390964402	7.35055688483291
O	-3.01613739059496	-14.83137458786227	7.81143501836297

H	-2.27678390663278	-15.33314287279671	8.18418105589616
N	-1.18720932535671	-10.45172161762681	8.38161407353522
N	-3.07695784515201	-9.28459037109701	6.05360011968465
C	-2.63196015267569	-13.69041628834077	7.24439375737484
C	-3.74371826465488	-13.07795240009788	6.42765072732697
H	-3.51878062458029	-13.32532776539436	5.38752124344152
H	-4.67962617161986	-13.57503368351982	6.68388842167090
C	-3.93707411389734	-11.56748401103608	6.57122473640469
H	-4.71200828188909	-11.26468047818566	5.86408602547178
H	-4.30789086495300	-11.33752850206890	7.57268827950048
C	-2.69869528673585	-10.70127826918065	6.27947915164055
C	-1.83835353700036	-10.62226443887948	7.45275173523246
C	-1.92500071059917	-11.12106567679874	5.02742659045437
H	-1.17424680729870	-10.36811191209835	4.78890192722058
H	-1.43380767198674	-12.07949906898041	5.17434550031683
H	-2.61969645924579	-11.19751104724445	4.19501516723544
O	10.90580218515731	-6.82893366202975	14.04177831192995
H	11.67027009964348	-6.31573367690493	13.63900691555611
O	11.45864535842008	-8.30381697806630	12.47726942147570
N	6.27304349147910	-10.50438752093365	13.86219841131790
N	8.71280690102498	-12.38191820568621	12.71175133595384
C	10.81249540111678	-8.00375370001632	13.46280807428059
C	9.85302209819299	-8.95884387790106	14.14729804662116
H	8.91644973221008	-8.44049492507072	14.38316302071509
H	10.31951093985036	-9.24293913318665	15.09175987957316
C	9.65716060163068	-10.16234636730965	13.23130979379601
H	10.63206215648313	-10.61572244082302	13.04237012130476
H	9.25986509947126	-9.79348124191533	12.27899429190103
C	8.74836988291336	-11.29432897254142	13.72901533347077
C	7.36502537936771	-10.85168540335831	13.84387477392989
C	9.23231111305176	-11.94029878812600	15.02516755816560
H	10.30748653392949	-12.09396621834778	14.98183871569431
H	8.75194721920444	-12.90871551932183	15.14663546098651
H	8.99587531355216	-11.31657576566218	15.88400531466732
O	9.61772963642540	-9.46192318429537	9.97303715560558
O	11.46925850701072	-10.46789441289714	10.70841218195543

H	11.56503549807707	-9.66761868055944	11.28741148116257
N	6.82346440797418	-12.53987197930456	9.08237745752034
N	8.88519368605981	-11.97449693847866	11.55337595776783
C	10.40187036356090	-10.38018058321410	9.92970355377693
C	10.29573676705534	-11.55871544482535	8.99069908155853
H	9.43432767248656	-11.42881154548879	8.33194246858930
H	11.20041654702136	-11.56552676179988	8.38007034078816
C	10.21597930316415	-12.88658723186493	9.75124623713985
H	10.28811883573610	-13.70931009288732	9.04024349737275
H	11.05392945034571	-12.96375612236222	10.44627207489980
C	8.91105184709142	-13.05406031862890	10.55414505602495
C	7.75088298169767	-12.80003927042234	9.70427302163736
C	8.82292998811336	-14.44370367559644	11.18234023490664
H	7.89565687263701	-14.55083605802320	11.74262419608734
H	8.86433094643097	-15.20102534074689	10.40444689771079
H	9.65929017473467	-14.58205325800629	11.86314532897530
O	1.21578115109014	-11.36531723369388	4.43656503489021
H	1.51592472895979	-11.17178171813044	3.51706461128680
O	2.75698306296883	-9.89275009896632	5.09405559609939
N	3.14857457981142	-8.15929999788758	7.92207773976288
N	4.11809454975979	-10.90964265703562	9.36479652883071
C	1.88049706462504	-10.68756906452579	5.35125732130211
C	1.41767056355738	-11.01030841181981	6.75506071464811
H	0.59283517337132	-11.72071512532897	6.69556662350212
H	1.04408327446571	-10.08209105896354	7.20365279020279
C	2.51113918375182	-11.62417014775795	7.63257676286712
H	2.88890281825254	-12.52948489913202	7.15559373419573
H	2.04648775261807	-11.92100714046983	8.57874175463706
C	3.70436892502887	-10.70110014233499	7.95701433653991
C	3.35927610001639	-9.28432268514249	7.88460576779743
C	4.92969895437689	-10.99723789572382	7.09298292603323
H	5.75711404715389	-10.33805959657525	7.36678560529175
H	4.69992325160661	-10.86213270668286	6.03943628656631
H	5.23788534744077	-12.02783012282312	7.25453769642564
O	0.55493531781967	-9.10541537218197	10.66580251364410
O	0.05098693721278	-11.27732736537897	10.55053199157662

H	-0.47926651956442	-11.01257436873566	9.72501756443570
N	4.20933650651965	-8.67109422529694	12.33518596171930
N	3.16716152904271	-10.83224580002446	10.15853958893119
C	0.59137523049474	-10.22368625755915	11.12764516250223
C	1.24359947794990	-10.55860205615595	12.45079176570194
H	1.62576232324625	-9.64313788520525	12.91225544801346
H	0.46598460195017	-10.96970633276785	13.09706287638709
C	2.34989884880607	-11.60514729372402	12.31143494758500
H	2.65861215735992	-11.93580677348813	13.30554948521488
H	1.96805082644744	-12.47465743352945	11.77247378821692
C	3.57917446438294	-11.07474591661176	11.55944182405893
C	3.96046533770745	-9.75232223744255	12.04594040459984
C	4.74266485029142	-12.06155364607194	11.63316773545631
H	4.47780332482632	-12.96205820022593	11.08307555983365
H	5.64404014887324	-11.63560669577840	11.19303100188210
H	4.94210900957473	-12.32452347601149	12.67011025477657
O	-0.39155194633418	-7.56691784332895	7.87442598737476
H	-0.25725441143614	-7.77358694329743	6.91086130639367
O	1.23184843522950	-6.04354435207711	7.67949553610121
N	-0.49034105829222	-7.40940760143545	13.10895445636015
N	-1.42877196816170	-4.60196181598940	11.65890058933630
C	0.36240523632820	-6.58127048240616	8.32278574102809
C	0.02047419635201	-6.19000012632238	9.74372865152261
H	0.29277557667335	-5.14015772084195	9.89542077918076
H	0.63654375803845	-6.79558718557476	10.41973088793585
C	-1.46141436726531	-6.42296530218217	10.02386523040787
H	-1.69653472763632	-7.48094372639034	9.90153390258161
H	-2.06367302585978	-5.85923534539582	9.30695283113006
C	-1.87425343915034	-5.98962053857878	11.43640574425268
C	-1.18990683713485	-6.82504056222083	12.41132854406076
C	-3.39370529938668	-5.97564775673770	11.58895075360522
H	-3.80930368959655	-6.95281674777925	11.36125914616303
H	-3.81081234317885	-5.23945332726994	10.90483548694535
H	-3.66140545716727	-5.70077976901040	12.60544891213318
O	-4.05468310379043	-2.00586413188438	9.96362476336852
O	-5.71430389302840	-1.73871542411063	11.43312947366524

H	-6.28677242628705	-1.57266445831313	10.67242760177962
N	1.29036285725233	-2.74286047539156	12.42756692398294
N	-1.56314581351635	-4.24676865438470	12.84171302094111
C	-4.45169990714140	-1.96989614696655	11.10364929617629
C	-3.59350786499184	-2.20506120982609	12.32398933534200
H	-3.93063415704931	-1.54177680444221	13.12009311671703
H	-3.79616691243940	-3.22973574452538	12.65422838507196
C	-2.10536186055644	-2.01795521794796	12.02390629987982
H	-1.88826303833774	-2.36921653029943	11.00797210726397
H	-1.84506259650862	-0.95978720647159	12.08819906985362
C	-1.24163422967972	-2.81417023848993	13.01639054533502
C	0.17808443697269	-2.70370731680038	12.70287650788744
C	-1.52037437651835	-2.42155767864800	14.46298589918826
H	-0.72136763710562	-2.75809926497389	15.12256270316106
H	-1.61063855954879	-1.34169478048963	14.54086892368380
H	-2.45850092524421	-2.86790403689644	14.78328233048241
O	-0.88005845461828	-6.67695104801891	4.09354672584091
H	-1.49178409484030	-6.50907515584317	4.86896245326368
O	0.01108561193153	-8.30119643032421	5.33538588821920
N	1.23846857679098	-4.83745453330306	5.15702074808983
N	3.41851082445877	-6.77177676268125	3.52923654720043
C	-0.05679076869814	-7.68242082209293	4.28910978907751
C	0.77055978272885	-8.02982771181043	3.08119183266115
H	0.12777774407336	-8.63174038374686	2.43606461746687
H	1.61206433048493	-8.65923231628865	3.38002097781176
C	1.24746426564075	-6.81250653963962	2.29150222841339
H	0.39963349093993	-6.18010986650815	2.02552525399365
H	1.71685402718422	-7.16046772136618	1.37120611246675
C	2.27722848090708	-5.96219506751732	3.05322355747863
C	1.67721735008455	-5.32752205383071	4.21849057198784
C	2.87139499266779	-4.90578522233192	2.11676741958106
H	3.37759729280196	-5.43171366071263	1.31076049187429
H	3.58861835535124	-4.27407751083201	2.64227790680075
H	2.08333691363416	-4.28595415435656	1.69876596030712
O	1.99020902913860	-10.82781173300819	1.83666630678392
O	2.81290950677848	-11.46333349405949	-0.13068633413859

H	2.03802664320161	-12.03552216916653	-0.19368613639964
N	5.48967454665562	-8.36470264367023	5.50524458847794
N	3.76122830483599	-7.58122606567078	2.65061662111349
C	2.85425658209147	-10.76262005517081	0.98673568632344
C	4.06589627846522	-9.86960498654874	1.06571845665938
H	4.80792906222918	-10.20253199279385	0.34171099105032
H	3.72680152045140	-8.87476362384717	0.75565397626631
C	4.64017853793160	-9.82161131681956	2.48850266837559
H	3.92437330457842	-10.26541478459819	3.18599776457743
H	5.55763780905703	-10.40873304722858	2.52994793224210
C	4.94125658060644	-8.38639507269404	2.95565000038978
C	5.24977653714122	-8.38521028097004	4.38447262484048
C	6.09609379827946	-7.75961425847388	2.16686485618560
H	5.88277327010760	-7.82279153330246	1.10368085384147
H	6.21249077778804	-6.71031614538694	2.43941853590389
H	7.02182979832081	-8.29122502686692	2.37783185862557
O	0.42541433296730	-8.92131913844106	15.00723473846168
H	0.05810118392335	-8.29962295278493	14.27083088851848
O	-1.73660475951793	-8.95117174833957	15.50707339986482
N	3.16611989523230	-9.30862563965342	16.95706680992996
N	0.71360509312175	-7.13814640566622	17.32562832198273
C	-0.60004794655905	-9.31033969293096	15.73738583715360
C	-0.24826949185979	-10.20070945457572	16.89926849502935
H	0.66972246841858	-10.75681829951903	16.69891086538752
H	-1.06276895369423	-10.90979691429846	17.05107401068163
C	-0.10521609502935	-9.35834665308282	18.17218352155667
H	0.07009805515812	-10.03215569222206	19.01123620379686
H	-1.03135995439552	-8.81084416458950	18.36263555296571
C	1.04382141215396	-8.33824625943743	18.11893835514217
C	2.24000964006021	-8.90602740621727	17.50324388926606
C	1.35658837129661	-7.82500103452048	19.52905173796711
H	1.64702090863261	-8.64787732969228	20.17603391619097
H	0.46509803542990	-7.34511813418518	19.92564411059070
H	2.16378758677137	-7.09682648162543	19.49684382617376
O	-4.44992509369329	-4.65108860701921	14.62783376272969
O	-4.97849767545548	-6.07558352498724	16.26005023252909

H	-5.87130461342315	-5.72019054735016	16.15941651811317
N	0.65089227265067	-5.06208126229626	14.81331619674424
N	-0.38846397387809	-6.65996023169304	17.64575506322077
C	-4.11864591301255	-5.47747991014495	15.44483887407665
C	-2.69307880786087	-5.91186056072729	15.67700639070833
H	-2.65910146857750	-6.98476265905366	15.87651128828185
H	-2.11082490897757	-5.68115467640562	14.77773844253089
C	-2.14398240311182	-5.13742383025116	16.88338516637898
H	-2.34976240875932	-4.07247274892604	16.76360104574231
H	-2.64180479188742	-5.47367263773320	17.79385983066556
C	-0.63267669938101	-5.33135803778083	17.06492980564478
C	0.08478685245121	-5.16271555257038	15.80548790820671
C	-0.07954482417713	-4.34972463337698	18.10661553989349
H	0.97916028364704	-4.54733635052393	18.26794613730259
H	-0.21252415702690	-3.32448611677429	17.76830880115650
H	-0.60894196115597	-4.48838395304714	19.04497150594093
O	4.30511643747330	-3.90524996458756	4.95820397736749
H	5.04913187522008	-4.58746494822318	4.88906364400050
O	4.46408275213648	-4.17557928625299	7.16995723817614
N	3.48167575153755	-4.19327302198625	10.13183253805053
N	3.48686618887753	-1.25003803307234	8.49643019833599
C	3.94939362410401	-3.65489675750759	6.20656453375359
C	2.85378007986614	-2.61650968303585	6.25934780352121
H	2.29060830342632	-2.66874008408927	5.32619757307219
H	3.34893781888260	-1.64033361647504	6.25978481339437
C	1.88317004520314	-2.72716187959761	7.43377240122037
H	1.52322975622309	-3.75623949585884	7.53625331447594
H	1.02195811811742	-2.10376708277379	7.19282449491365
C	2.41974021280448	-2.22510885516444	8.79608704359364
C	3.02451390462125	-3.32418635683028	9.54037772892983
C	1.27838117562833	-1.60652592950368	9.60572396682915
H	1.64700004166420	-1.19292268034595	10.54525296218568
H	0.51969460098731	-2.36099697273700	9.81570382979686
H	0.81792803677338	-0.80384187017397	9.03272459519446
O	1.36776517536682	0.09295456236419	6.24216208205449
O	1.35570809362172	1.21049727742377	4.31533541520379

H	0.61411592784138	0.61464442885467	4.06436300435822
N	5.76789094907942	-0.42094613664793	6.55214228866061
N	3.04789203997971	-0.08762706235343	8.42742834329395
C	1.82670295669584	0.95021826584830	5.52382453099169
C	3.00156027772652	1.84732317141864	5.83762639716081
H	3.86276111505740	1.44540631618918	5.29210576235634
H	2.78626711662771	2.82054529387717	5.39340856748978
C	3.34008383514175	2.05074495833569	7.31523563835559
H	3.98036009960404	2.93223010981876	7.38030640963638
H	2.42360717483112	2.25759062496111	7.87380020645270
C	4.05849238710122	0.88870105508482	8.01784390216013
C	5.04116267673091	0.21891490590025	7.16888381519190
C	4.73490451446429	1.37602389387522	9.30461969752397
H	4.00734820087551	1.88930713504356	9.92953693376403
H	5.14743843854438	0.52964253907494	9.85893186656220
H	5.53511513567451	2.07026961666727	9.06067809871191
O	13.28996425913437	-8.66721015135393	5.99765099114094
H	13.72933177502411	-8.64320137907107	6.91871777422568
O	11.40417042616488	-9.20681198064773	7.05824809074768
N	8.27359820242276	-9.61900331048139	7.42801168795420
N	8.59475693724204	-8.32567161734211	4.40719725114605
C	12.02625423736728	-8.98973820331286	6.03106937221889
C	11.40865802441947	-9.04014024503156	4.65657038306958
H	12.20235266508366	-9.23226073765752	3.93546289521667
H	11.01940730258220	-8.03749442645105	4.44829562844788
C	10.31033402090911	-10.08843470539015	4.48881579775863
H	10.57096092538827	-10.99280085119993	5.04129563404222
H	10.26422428013259	-10.34832721221287	3.43106902231283
C	8.88109738765657	-9.67764800201427	4.90564435878822
C	8.63936549723013	-9.67354231595387	6.34256904398189
C	7.88591457218443	-10.64803269233316	4.26143454861542
H	8.13371665467963	-11.67064345609442	4.53358899517846
H	7.94433660222610	-10.54840403436342	3.18100716011763
H	6.86926279885404	-10.42745158735451	4.58563821630645
O	8.40509587606316	-3.52649108875869	8.54298867631717
O	10.51241064297325	-3.64446480339119	7.80840821575210

H	10.69716765784844	-2.80439362989264	8.30091310106725
N	6.27987080476007	-5.63999928728995	4.86841928448685
N	9.05486257331824	-7.43895925274576	5.14960073681689
C	9.26465329829707	-4.05654503152077	7.87342364270255
C	9.00204933920640	-5.27766671200590	7.02190887285168
H	9.54469863100283	-6.12269523946075	7.45654646075172
H	7.93031206988603	-5.50175741016243	7.06328352391345
C	9.47093962480916	-5.06204198719211	5.57849599206120
H	9.20793220007137	-4.05648068771104	5.24066609557705
H	10.55596251947057	-5.16041630213664	5.52074835931035
C	8.82579197269562	-6.07437589074153	4.61673540946726
C	7.39126365374583	-5.83976374675612	4.66450660913745
C	9.39098541852444	-5.97348081784566	3.20250550939577
H	9.01780410616764	-6.79747009546405	2.59922415239290
H	9.10066777647372	-5.02998380180919	2.74559468700881
H	10.47674786032101	-6.02604348929714	3.24074667077015
O	15.90329179206600	6.02037064953832	1.33541333301904
H	16.52462877856231	6.04544041109383	0.59695562143032
O	16.52248844878331	3.88816320708420	1.55641927599764
N	11.50737661343542	3.37423358138130	3.66174898431552
N	14.00350341119442	1.88142879830411	5.34013201189764
C	15.86263059171867	4.82799784991590	1.91835373532543
C	14.88693351174678	4.79745053831009	3.07333892493010
H	13.88665368425493	4.96889507480121	2.66627573246232
H	15.11674967478863	5.63205834412636	3.73735955996764
C	14.96460256940439	3.45756759726365	3.80125980798519
H	15.91531241642707	3.37065925993529	4.33148261903905
H	14.91587208857308	2.64430327491099	3.07156545248617
C	13.83575066303432	3.26040839872046	4.83074009429400
C	12.53438381633675	3.32580042141170	4.17238554656848
C	13.89067193323366	4.26767551034321	5.98193163965501
H	13.15571682007091	3.99952687699639	6.73918681832956
H	13.67570397672269	5.26870246092033	5.61747328015192
H	14.88068085449065	4.24952070099679	6.43098029957314
O	14.44624430408479	-2.44371870683046	4.13535249694819
O	16.66889337714245	-2.57075369575168	3.96907763215055

H	16.54650332645630	-3.55756156624620	3.87752050369486
N	13.04951261649329	-0.92485412207043	6.58036973219953
N	14.96529424674302	1.81537802233394	6.12636877149933
C	15.53673280770203	-1.91488187245592	4.09417206918790
C	15.76512255655605	-0.42371078865396	4.21426355564730
H	16.49072349535363	-0.12988705187855	3.45506352745854
H	14.83264028656587	0.11661020522296	4.03829646203686
C	16.32921539489402	-0.08957883798149	5.60570811220004
H	16.79888481671484	-0.97113801117112	6.04562699786352
H	17.09005464532513	0.68669881383814	5.51665630894873
C	15.25947123694287	0.44895498721732	6.57967377586904
C	14.04213831934395	-0.34854642499652	6.56687806377815
C	15.81931858839684	0.53240536844933	8.00054859258359
H	16.05540738507814	-0.46203716043680	8.37223418329181
H	16.72014502752226	1.14032161331110	7.99473392796692
H	15.08603531661282	0.99399185882613	8.65667104631364
O	6.09154594794651	0.97490531649224	16.25321605804486
H	6.67394672127328	0.30834081298999	16.73479493134699
O	8.03567869375530	1.73256113725066	15.47481885109482
N	5.02003652904627	0.92192941746333	12.45168706718257
N	2.79558445228743	3.05300980422913	13.69357276027921
C	6.84908153865924	1.89734835944870	15.69946123483012
C	6.16053704944990	3.20978535047386	15.44180962582238
H	6.69150793926878	3.73914441459032	14.65329709657640
H	6.32819063443147	3.77379200990213	16.36224631347726
C	4.65585741244413	3.14441278863199	15.18229247863569
H	4.18761857150208	4.04348536322358	15.58432463078526
H	4.21024525281394	2.28603910744582	15.69311011781159
C	4.27407117662007	3.08268692449977	13.69153182001787
C	4.74329817874959	1.87423362505711	13.02745926088102
C	4.70609465727628	4.33168067143697	12.92617003840462
H	5.78641856201460	4.44075325141691	12.92663538440802
H	4.26571161562027	5.20442764472123	13.40048016756974
H	4.36663905968410	4.28022546079626	11.89188887122955
O	1.83440466282616	-1.72126682556162	15.73049304509884
O	2.88042989748061	0.14298749956565	16.38380654103433

H	3.67406362330933	-0.43571135219688	16.52939402748583
N	0.10414108653962	0.09548447582450	12.65346995977612
N	2.34803203075334	1.97105369117847	14.10612650941839
C	1.81240378731183	-0.55144837979594	16.03975994263619
C	0.53330823729546	0.24461489059196	16.15031153207970
H	-0.25579914568279	-0.26216201393502	15.59509196249825
H	0.26681668292872	0.19833664902935	17.20889475740789
C	0.62043315033864	1.71898903320840	15.75926283853340
H	-0.32217740442216	2.19294301160126	16.03153360205694
H	1.41513059061170	2.20778084550878	16.32587699776711
C	0.88092220310555	1.98867356899682	14.26071104422680
C	0.40012725932983	0.92239009763981	13.39052967216978
C	0.25298271969739	3.31638202532020	13.83625817164268
H	0.66619037789385	4.11963394328967	14.44030503283489
H	0.46523417689220	3.52093682240571	12.78849830993066
H	-0.82316347973736	3.27358082891815	13.98186126461776
O	10.58166572380331	7.45816860482615	10.22389244793042
H	10.50085632221205	8.30548965802163	9.76571259320175
O	8.71954910462381	6.90576058712318	9.12915014575833
N	7.46012155794146	2.88148846916911	12.20453148515165
N	10.57022957128245	3.43811432626987	13.01808760474646
C	9.59434855437109	6.62832547386493	9.91595614456901
C	9.66590201506348	5.33044102000467	10.68602820429914
H	10.61668713100806	4.84008682611006	10.46783208174365
H	8.84187116251688	4.68638983221492	10.36521052825397
C	9.58927399271420	5.62823451334248	12.18756111386775
H	8.73399966887586	6.27479686953401	12.39329652548489
H	10.49466321957842	6.15060504976363	12.50054673102317
C	9.43373592447840	4.36425115533594	13.04307478941809
C	8.30034838398648	3.56026450887901	12.59058645100333
C	9.28922106275952	4.73815541772798	14.52530560915242
H	9.18807515367567	3.83865277853159	15.12927623680730
H	8.42091426310084	5.37563836765001	14.66752889915566
H	10.18227593987472	5.27493252546094	14.83587693000989
O	12.70572652873834	2.22727280848717	8.86706824789692
O	12.88732424993038	4.32554702987577	9.57900999324030

H	12.20167691239332	4.53066701027975	8.84810083087398
N	11.70942701879579	0.71084218469514	11.93307334423786
N	11.63176313697719	3.92269789312624	12.59071216634648
C	13.18806655676000	3.06451528857815	9.62652215300174
C	14.21506247125374	2.71656359774335	10.67013670362199
H	14.26214366706096	1.63426430956094	10.79678438834867
H	15.17439359068482	3.04979294865254	10.26780964044232
C	13.98761712740495	3.42443739222352	12.00921064295187
H	14.86637502466762	3.24945531997419	12.63027283348843
H	13.89874857660915	4.50042830120641	11.84783446800658
C	12.73828929312893	2.94874498978965	12.77152921325593
C	12.22570653309125	1.66431126689058	12.30765299916098
C	13.00513813811738	2.88953949210152	14.28150708425609
H	13.78623164424569	2.16432543601242	14.49468538821357
H	13.32061271852851	3.87136792733863	14.62543429486296
H	12.08972937210095	2.60834743127654	14.80023905088360
O	15.32454655870211	-7.08303582171470	3.09211508628131
H	16.01676631360586	-7.18563809820795	2.42624576293989
O	16.44184563874319	-5.23395757959236	3.60400008748222
N	11.91167967806043	-6.21103405124070	7.28970788777453
N	14.90675915053686	-5.53289267293824	8.66276233866734
C	15.51411902911863	-5.99005991665794	3.80640726348798
C	14.48878968442070	-5.77045155854598	4.88711789255806
H	13.69834021813201	-6.52002502231880	4.81803866656731
H	14.06394052970770	-4.77668272070538	4.71718585622327
C	15.17684797773348	-5.81960863883021	6.25941434639182
H	16.12101189314938	-5.27385317118247	6.21878593744329
H	15.39425385580162	-6.85579387668859	6.52891343397387
C	14.30583201013471	-5.19163687312166	7.35903578531665
C	12.95324188747369	-5.73267970436973	7.30416921995890
C	14.31160349065430	-3.66439797841957	7.27408617456302
H	15.31275571635707	-3.30129773988845	7.49160024895662
H	13.61895918533580	-3.24203884224755	7.99826074383123
H	14.02918461476657	-3.34010555722655	6.27645659539631
O	14.37626975089523	-8.49931963389911	8.34751856932259
O	12.39085270759739	-8.29167686788885	9.32546893248712

H	12.03787725151884	-8.55383790508703	8.41159819786844
N	12.89390685035929	-5.62002890515475	12.70182204073816
N	14.09693381573307	-5.46312085837861	9.60430707129136
C	13.69182113341274	-8.35617534354825	9.35193946717058
C	14.30268866719721	-8.28573734754875	10.72734263276078
H	13.52580865840957	-8.16616335330861	11.48468553802035
H	14.79655840769140	-9.24644412374666	10.88657114694894
C	15.36221881493454	-7.18696196551802	10.83549331998996
H	15.95103451810210	-7.34478493435861	11.73800692181429
H	16.02954418454480	-7.23153581364824	9.97202028036600
C	14.76139297838065	-5.77022182754509	10.88863814119976
C	13.73535263326499	-5.68748802286416	11.92340985702670
C	15.86475006658400	-4.73811316081605	11.13792741932965
H	16.38629393958090	-4.96328745282656	12.06376632041279
H	16.56469957907916	-4.77574189940596	10.30664102610479
H	15.44602938188931	-3.73692477125272	11.20048136739877
O	-2.07735355424090	5.31158812907304	7.69014977959340
H	-2.51735818725124	5.34483184535811	6.82977924567301
O	-0.42124042554063	4.22555917084176	6.65800987388359
N	1.77958010323541	2.26998505685541	10.91805649229416
N	3.55495542435307	4.85241490469814	9.88537294203986
C	-0.87814374696261	4.74364546283322	7.64629017500564
C	-0.18426458481019	4.80033484023219	8.98856950081545
H	-0.37372417596181	3.83831950910013	9.47840866064120
H	-0.64084753049974	5.58331646726365	9.59208294938294
C	1.32061484465604	5.01764423480711	8.82445265496201
H	1.51917729231737	6.05082679456299	8.53470192781008
H	1.69833298871280	4.36793185121177	8.02825418799528
C	2.09562747606720	4.73277353188126	10.12245442398292
C	1.88010966932862	3.36030632718153	10.57660698687041
C	1.74218597121335	5.72694035033948	11.23311525421335
H	0.67520065819568	5.70992340166696	11.43795953136178
H	2.03405265651434	6.72406808511292	10.91216859973227
H	2.27786128189448	5.47896254188855	12.14669190920690
O	6.14701901046009	5.91776542683939	4.55694752551533
O	4.73381040093318	7.53444984241544	5.15858744945476

H	4.95548971908266	7.93802346343752	4.31056483637700
N	6.71399051290470	4.03223603491701	9.54592615428575
N	3.83955792732836	5.93606187776282	9.34493219474305
C	5.39489038505078	6.40307055378303	5.36641174328898
C	5.09757317241294	5.81000722906461	6.72379715471390
H	4.01765275343740	5.65566768243456	6.80531314328663
H	5.60579561496455	4.84595715493075	6.80809306048240
C	5.55202378274445	6.77101153916437	7.82963785120804
H	6.62080990884099	6.97480390903715	7.74089487715397
H	5.01155285186054	7.71402563314646	7.73644840287974
C	5.27880447186500	6.18485266566134	9.22309711314077
C	6.07481139407822	4.97606031480892	9.41847507124756
C	5.60844784916450	7.20396665893813	10.32038167828167
H	5.41123364278371	6.77061839533644	11.29853340696770
H	6.65607136220507	7.48617737475061	10.25394973066314
H	4.98606277204040	8.08551037742673	10.19081054393043
O	5.64682386886934	-1.26472036090136	19.33719011176542
H	6.40490734276287	-0.84083935343228	18.87328234379174
O	4.81626525276337	-1.53130514166830	17.28859554209086
N	3.13933733427414	-5.56631662448547	17.69496462909562
N	5.83276673635098	-4.28284669608789	19.15984160633159
C	4.72622917639278	-1.67584026278342	18.49225458560814
C	3.52254304398241	-2.29812705134539	19.15487872549842
H	2.87216251112943	-2.72221450538941	18.38506347966369
H	2.98522521412902	-1.47780424179976	19.63584107765548
C	3.86880544797404	-3.32838350670270	20.23529553585721
H	2.95679810183663	-3.59031569346681	20.77204276523058
H	4.56753489958330	-2.88523016242574	20.94610299341075
C	4.50576970508116	-4.62045952790762	19.69504588326421
C	3.71498951523796	-5.18087019937752	18.60911934112959
C	4.70975784825153	-5.62689225370762	20.83037354001939
H	5.30240994185230	-5.16143702740542	21.61344852907951
H	5.24365250915339	-6.49856853435772	20.45970058677693
H	3.75110991796030	-5.93213616332370	21.24144104502045
O	7.72197081829917	-0.62420354897905	17.50026516298005
O	9.26430755216372	-0.62158306756980	15.90762351939424

H	8.95536418061019	0.32998247042618	15.81665304420319
N	8.33591768772793	-6.10677485308224	15.92813204828663
N	6.38078811773583	-5.19592623012048	18.51910325708028
C	8.58576052254656	-1.20702451981454	16.85584199626148
C	8.91454020386709	-2.66061899279287	17.09566814162986
H	9.14077064047608	-3.14817277288665	16.14416603973045
H	9.81087327771093	-2.70364245752504	17.71618138376766
C	7.70975535248834	-3.26907652997363	17.80644757998264
H	7.59544554694681	-2.76774191914247	18.76727371478919
H	6.81636482131387	-3.05442408077505	17.20988608952331
C	7.73956484074531	-4.77733516585049	18.08674545019869
C	8.08202610743282	-5.53137398833580	16.88699146654176
C	8.67992610423449	-5.14542830786213	19.23733614963500
H	9.70405815674709	-4.88560155413984	18.98351501832440
H	8.37573392328385	-4.59649386101306	20.12493623559791
H	8.62012323203954	-6.21154034162703	19.44586924755776
O	2.17420916559127	-13.65198541960191	20.03521736975387
H	1.42106243042362	-14.00776171534071	20.52263023863636
O	0.66929452543389	-12.37059239912553	19.00462948198034
N	4.27177022143601	-12.41284086033421	14.98744119773647
N	5.53451564418140	-11.07692340239836	17.69346401287877
C	1.81473295891049	-12.71560398493499	19.16711763461710
C	2.97529072840458	-12.11731436300652	18.40975046774272
H	3.21226255573045	-11.16387430467162	18.89356669821080
H	2.63336869686721	-11.88985525213355	17.39467474261011
C	4.21652966059503	-13.00781705802862	18.39258550659102
H	3.94632272815493	-14.03085767302989	18.12480208286091
H	4.66649183327320	-13.03038070808921	19.38608485359207
C	5.26763312896528	-12.49971862941955	17.39385224449006
C	4.71484032777423	-12.49049734600012	16.04210180910684
C	6.54139661947957	-13.33975969920875	17.46519092354476
H	6.30532126311717	-14.38715815457588	17.29658287853522
H	6.98431323392649	-13.22687394801223	18.45151099154496
H	7.25970058320698	-13.00779730159295	16.71968745158138
O	3.82052562509881	-9.55485671197873	20.49307489939953
O	5.19737964267212	-8.74691091552356	22.05103727870251

H	4.41872929168884	-8.71081772817046	22.62384167087330
N	6.32163884034451	-8.05238367991305	15.31542100097403
N	6.62935535783246	-10.68415763810162	17.26169966246668
C	4.93111511621685	-9.24555918850090	20.85275428940545
C	6.16740242173948	-9.38975327138996	19.99662317665833
H	7.03265269351849	-8.99762844253877	20.52809390134237
H	6.31088665330438	-10.46166130314881	19.83382486093220
C	5.96713909097130	-8.68773338628772	18.64845080644779
H	4.92466443261367	-8.80251757204278	18.33193230728940
H	6.17849494378252	-7.62010429164634	18.75262667689646
C	6.88516308944730	-9.25673499292909	17.55557340244947
C	6.58812811031081	-8.56665404279586	16.30325476023881
C	8.36931834226569	-9.12476593878886	17.89636983426721
H	8.98053966406808	-9.48975463914208	17.07314222984552
H	8.61975831557908	-8.08464757329636	18.09126017134889
H	8.59358988825638	-9.71532177695506	18.77965207482288
O	9.22376599627600	0.27304659048927	7.15478721258235
H	9.38835677472229	1.06876887956605	6.61017943078368
O	7.72958032766304	1.57619368970236	8.16429951544252
N	5.62562453440716	-2.00103160785645	11.16141061176736
N	8.89018780987345	-2.11225268132775	11.76660448456640
C	8.35725328783869	0.54369162969823	8.11760282154667
C	8.21598693971360	-0.55583776581824	9.14312536041735
H	8.95659275593687	-1.33461497055866	8.96385950066893
H	7.21344521549681	-0.98885545118268	9.02218467814615
C	8.38003703309122	0.02685783307237	10.54920028117523
H	7.77860494658260	0.93488453883207	10.65126836234208
H	9.42537964237504	0.29875020947940	10.71608641749813
C	7.96545846116020	-0.96051094401558	11.65074084044340
C	6.64220627588066	-1.51805551799742	11.38329985768185
C	7.98506271382232	-0.28164253777900	13.02262460508204
H	8.98334454963379	0.10991151684206	13.20339245217123
H	7.74458370681236	-0.99974634563679	13.80518909078744
H	7.26889325621135	0.53635140569654	13.05169457972290
O	11.26580080202485	-1.33478898869832	9.05364925923523
O	13.14237019686309	-0.31483705469787	9.70200270524588

H	12.83087702733378	0.50912707366737	9.24380458772980
N	10.26826323856684	-4.96288404185247	10.90172290941982
N	10.07483741781485	-1.74070482775619	11.83633995501788
C	12.32213641862319	-1.33729284019952	9.65197254979574
C	12.85026001841041	-2.51278173861958	10.43562132679966
H	12.49940210456178	-3.45326070799233	10.00130295337721
H	13.93679763493374	-2.48190816215180	10.38247716748515
C	12.44260898795022	-2.41047783744126	11.91173368636531
H	13.10470567942374	-3.05329005492843	12.49168292985767
H	12.58141159760434	-1.38540789001902	12.26051724170248
C	10.99544052097831	-2.82618164438710	12.20138083326247
C	10.62297558790151	-4.04447244917870	11.49028654560998
C	10.81446973315082	-3.00082932702170	13.71592376302517
H	11.47303896826701	-3.78354727535456	14.08354566169396
H	11.06309206152119	-2.06564048906784	14.21207565854313
H	9.77857778481010	-3.25278085913329	13.94193523952181
O	4.82775917304689	-1.07942717128741	14.54246980251181
H	5.45232422356234	-0.55918837795971	15.07807084037320
O	6.29063456098732	-2.74028467664728	14.82056634138530
N	2.11168683594411	-5.78897581175098	12.00794325178801
N	5.12133562620447	-6.03039719981224	13.51841090295367
C	5.20238424711151	-2.34428245210736	14.46335670862917
C	4.14589336293054	-3.24158054419839	13.85571087691580
H	4.54159022247408	-3.58029253718581	12.88475315586435
H	3.22934557724216	-2.67300700108587	13.67820696948214
C	3.87592526058042	-4.43974443647722	14.77328504758728
H	2.95491074775774	-4.27419104879906	15.33586403570383
H	4.68874249696172	-4.54773366946018	15.49642081440887
C	3.76993085053168	-5.77703893904416	14.01239120151157
C	2.81714042845534	-5.73323188614471	12.90992345425435
C	3.39861654238851	-6.92041304025157	14.95671718270810
H	4.12470165687329	-6.96767460770574	15.76561763086622
H	3.40643088974723	-7.87221672572306	14.42060715644560
H	2.41045008802521	-6.75770381461382	15.38380744305653
O	5.89039427624449	-8.34566175104973	9.27940931348276
O	7.88666508754630	-7.39148331038103	8.97540827016094

H	8.19950521038712	-8.30914139641487	8.76169867002632
N	7.39504346405192	-8.27129721319611	12.15321036134034
N	5.35185286648157	-5.57514206104195	12.38212323142919
C	6.59648949012840	-7.36516771812697	9.26896693138874
C	6.10285757094466	-5.96289663789199	9.56200690711394
H	6.13819697951751	-5.40662491338011	8.61631254957631
H	5.05791537582742	-6.00569679768308	9.89318021847799
C	6.97362170870855	-5.23123950190645	10.58656897618447
H	8.02842595060088	-5.33252702276193	10.32152475536914
H	6.72920600284975	-4.16435580219175	10.57155271096003
C	6.77612726471248	-5.74301879244244	12.02045375761582
C	7.12677429718647	-7.15578712986609	12.11216210964231
C	7.62166785198052	-4.95883303519650	13.02628631411808
H	8.66474507477966	-5.26003072594796	12.96007217286351
H	7.55089372357592	-3.88881396278429	12.83616841321150
H	7.26143664497467	-5.15898360973062	14.03391315710296

RR-RS

```
:::::::::::::::::::::::::::::::::::::::::::::::::::::
::                  THERMODYNAMICS                  ::
:::::::::::::::::::::::::::::::::::::::::::::::::::::
:: total free energy       -1371.539113008540 Eh   ::
::.................................................::
:: total energy            -1376.804432407017 Eh   ::
:: zero point energy           5.793060887059 Eh   ::
:: G(RRHO) w/o ZPVE           -0.527741488582 Eh   ::
:: G(RRHO) contrib.            5.265319398477 Eh   ::
:::::::::::::::::::::::::::::::::::::::::::::::::::::
```

O	1.33582718783512	-5.80501603001434	0.18745043507455
O	2.83399091229756	-7.19885416416442	1.07978607784157

H	2.47556630400583	-6.92854362732162	1.93716891164686
O	-5.00317477936655	-10.36953418676572	-2.70173064749935
N	0.59886150539984	-8.38088773239904	-4.03119725408599
N	0.64417960265118	-8.69090208986978	-0.66747339223835
N	-0.51490132680624	-8.53090082727896	-1.08498987520443
N	-2.41530010638027	-5.84566112839435	-0.57274175257124
C	2.24495735782424	-6.58736619254655	0.05790788833979
C	2.87101332913111	-6.96023533370926	-1.26660345152406
H	3.88720790224826	-6.55973859570415	-1.25844009713880
H	2.31245625089306	-6.48165481813532	-2.07467570575429
C	2.95172394527668	-8.47865713164294	-1.45902704624760
H	3.36928874167047	-8.93650587251029	-0.56150667651597
H	3.61110582187194	-8.70849403192663	-2.29728612226662
C	1.57541118202165	-9.10504471962006	-1.72763555959593
C	1.05376829086943	-8.69754935227208	-3.02550555750177
C	1.63368932482804	-10.63660758372207	-1.66494010597715
H	2.33524992644645	-11.00946259835099	-2.40637678949351
H	0.64431362956218	-11.04707335598246	-1.85628680838505
H	1.95864823817207	-10.93892341254707	-0.67316235617265
C	-1.98976233503517	-6.88091656025976	-0.31935004967892
C	-0.85490878800822	-8.22231071327574	1.38268858925039
H	-1.62923389282686	-8.04078324398656	2.12338120867633
H	-0.09042430226031	-7.45242528023462	1.46486637931260
H	-0.39960772845924	-9.19261644365876	1.56518196442497
C	-1.47420770379239	-8.20914121151599	-0.01363686344009
C	-2.61671729526580	-9.23819762531041	-0.13816601697003
H	-2.20788308298012	-10.22394534034810	0.08920646287605
H	-3.37651453357933	-9.00712825269074	0.60948924953134
C	-3.23560515143760	-9.24414370325261	-1.53624967939109
H	-3.60174489711632	-8.24788189500335	-1.80670188107653
H	-2.49075487583477	-9.52740777753030	-2.28648483030122
C	-4.40230312681110	-10.19572435179927	-1.66874386035547
O	-4.71089118831989	-10.81806570722593	-0.54070182284990
H	-5.45896662307963	-11.41962601836324	-0.64297438020212
O	-3.31364925547915	8.27600177337542	-12.26824818732940
H	-2.75200700745046	8.12364858453693	-11.46946296683672

O	-4.50700227781248	6.52826472786460	-11.57217002605598
N	-3.73773780772071	5.51623635632783	-15.92660806667609
N	-6.88613840810624	4.96953597582483	-15.48244949811702
C	-4.33012191361233	7.44023382452326	-12.34677278987640
C	-5.25168637290900	7.80305420408459	-13.49285879179659
H	-4.62820822287066	8.07294829312865	-14.34714909028072
H	-5.75370894463947	8.71987781458832	-13.17951174928835
C	-6.29135627276320	6.73060978012530	-13.85423735252064
H	-7.29843393085552	7.13088178177659	-13.72892162470569
H	-6.20165957016877	5.87313866964636	-13.18209660487310
C	-6.15870518431860	6.25786119815333	-15.31310392089430
C	-4.78810542586879	5.88084512498258	-15.63292159052978
C	-6.63271665684704	7.32800839330994	-16.29891081891878
H	-6.44788626839960	7.01176466010836	-17.32288766303123
H	-6.10833877113919	8.26133693140772	-16.11320509543850
H	-7.70125340653986	7.48263211321911	-16.16813091131205
O	-9.45272331228604	5.16944525133766	-14.01696337512216
O	-10.07909497774494	3.32174217297105	-12.93978750494284
H	-9.95183484165549	3.89169184009531	-12.10294286096392
N	-7.24193886677897	1.63457188027856	-15.75751714407364
N	-7.56985137662909	4.98611284400100	-16.51782262806793
C	-9.87824099441666	4.02397277067125	-14.01962392092040
C	-10.28233147656405	3.30379507362870	-15.27886373188275
H	-9.99036166736140	2.25314737615305	-15.21768883134271
H	-11.37506762425690	3.32674644500763	-15.28287641480518
C	-9.78052036527751	3.96789776067590	-16.55666536059411
H	-10.35964910849215	3.57531732935472	-17.39211739526316
H	-9.95650995120304	5.04434899767725	-16.50280892566807
C	-8.27988844213577	3.75155816840763	-16.86364841397470
C	-7.71840047682529	2.57624672373579	-16.21090325035178
C	-8.08435191271512	3.59124362068301	-18.37902516915082
H	-8.58383750329407	2.69209763562332	-18.73100679107456
H	-8.50761822552415	4.45784593526542	-18.87915977942895
H	-7.02401926743347	3.53263356585351	-18.61497018201694
O	-12.39090792666993	-0.36825470281651	-16.27079469746247
O	-14.56650709357739	-0.60934607987971	-16.67529183429621

H	-14.34080495022650	-0.46768659033142	-17.60443260593123
O	-12.13252085282353	-6.96405322534006	-9.16801780818122
N	-11.25900538717878	0.89329702295294	-10.69929713956164
N	-11.96149083769870	-1.84886987561268	-12.48984396515245
N	-12.65969679634005	-2.87772411695958	-12.55890362587386
N	-9.42319727215835	-3.95296717838953	-12.40403574031033
C	-13.51945056131633	-0.55160658832614	-15.87222316052167
C	-13.90568482014065	-0.77317557369713	-14.42943187335579
H	-13.74571184904119	-1.83888012449313	-14.23259679946718
H	-14.96981431587784	-0.57543784920125	-14.30899876057809
C	-13.06264631192943	0.06540973800737	-13.46794001906983
H	-13.58111189192529	0.99648857559317	-13.23720530267047
H	-12.10743106047759	0.31871631213864	-13.93524658645207
C	-12.77123844695378	-0.66927503239103	-12.13884875372309
C	-11.93224378792648	0.20977324350559	-11.32881369029264
C	-14.04013545279523	-1.02558655156113	-11.36473489223138
H	-14.69357232386423	-1.63831250280628	-11.97897162175869
H	-14.56402697842578	-0.11595533192559	-11.08353523329147
H	-13.77996220382134	-1.58561753262073	-10.46783617623405
C	-10.52213328192344	-3.99896183723721	-12.72655227523674
C	-12.18380932953085	-4.10136196239554	-14.56426648830616
H	-11.70458832119543	-3.24617103857813	-15.03897586225968
H	-13.25345809125678	-4.06137272431768	-14.75325733449052
H	-11.79132568215100	-5.02409343488630	-14.98531410269406
C	-11.94464030885501	-4.06904455289807	-13.05122135310783
C	-12.56792357549386	-5.30398005424099	-12.37871670920269
H	-13.64273418933331	-5.29272136659509	-12.56071175995616
H	-12.15390398152775	-6.20609383383178	-12.83392558984052
C	-12.28830998785363	-5.29543663046235	-10.87630158333773
H	-11.22478760231932	-5.13103598579031	-10.68077730009206
H	-12.84188351941250	-4.47748408146076	-10.40474294876530
C	-12.69341472909344	-6.56168767523123	-10.16442707190217
O	-13.72526528492458	-7.17669239942815	-10.71181404383452
H	-14.01973082845600	-7.94460412589296	-10.20546375653239
O	-10.34921168794831	3.21388773902738	-4.10512009186352
H	-10.94162791359108	2.52135326205620	-3.78294969574886

O	-8.83163385147832	1.59867957291897	-4.34094925049743
N	-3.78626626523377	3.20175810562597	-6.62320310705561
N	-6.75082236105798	4.89653489807541	-6.99042988536845
C	-9.14054989881693	2.76917330318713	-4.39889911782594
C	-8.22233438455003	3.89220071929467	-4.81432608961890
H	-8.25377821158020	4.65513879176270	-4.03548018670120
H	-8.64504545825803	4.34200318117285	-5.71695278012853
C	-6.79663665362514	3.40255939771354	-5.06571953115019
H	-6.83681792820850	2.46544147459752	-5.63164786101945
H	-6.30677089536017	3.20062073523127	-4.11192839337220
C	-5.95132131174411	4.42461963153920	-5.84874650026741
C	-4.72488661321739	3.76598031960111	-6.28129285485162
C	-5.61174717688307	5.67494063180745	-5.02904047484786
H	-4.96568735129394	6.32828539079868	-5.61414072023403
H	-5.10621240990071	5.39320434400525	-4.10829475654916
H	-6.52644421947635	6.20902050361826	-4.78753326767211
O	-9.71535844979284	4.73230944233719	-10.80356896713960
O	-9.22731014672456	6.62138138014179	-11.88394271753609
H	-9.34902659371475	6.06795220576138	-12.73588724600426
N	-6.48227892063678	4.64982072730639	-11.07590008848373
N	-6.09686498243844	5.43076790277706	-7.89782034302292
C	-9.45261129782645	5.92626873524350	-10.80810325318746
C	-9.36883783483041	6.71314007405931	-9.52456553581036
H	-9.06640571789661	7.73820247716032	-9.72926576276644
H	-10.37460820767635	6.73379247423572	-9.10062232787191
C	-8.44515028767275	6.02319447617617	-8.51844607521853
H	-8.52365944040336	6.52615877009186	-7.55404677728780
H	-8.77214142145512	4.98816309960084	-8.38552813971224
C	-6.96403029474630	6.04502578635134	-8.93187342351405
C	-6.72293259674649	5.27135476473116	-10.14201646230897
C	-6.42983034048760	7.46862740611529	-9.12827332799875
H	-6.59590366862010	8.04012628852657	-8.21896356775227
H	-5.36447111195485	7.43948062298861	-9.34314218891335
H	-6.93707223399894	7.95395327251431	-9.95755645679888
O	-7.16091476470904	-14.07393927775350	-14.16190178288339
O	-6.38723616129406	-16.15803797126740	-14.01402764662476

H	-7.27715943844499	-16.40023094472433	-13.72793229597836
O	0.72976781283586	-9.39443495688182	-19.65984008690735
N	-3.45539224274482	-10.81904578982681	-15.58446654356281
N	-3.77775980990455	-13.08933040682198	-18.08109767846002
N	-3.34803108532679	-12.02219363167603	-18.54587311135709
N	-5.04693556782209	-10.05018741646703	-20.60721160732981
C	-6.25831856572058	-14.87018921470540	-14.27964477229869
C	-4.86640521920435	-14.50571072124978	-14.74879704683379
H	-4.23152674733361	-15.39038579030881	-14.72880033898194
H	-4.46533016515847	-13.76313998653420	-14.05444962954923
C	-4.94963828520633	-13.91470268942396	-16.16097645160299
H	-5.77822317682565	-13.20258802277588	-16.21144461334614
H	-5.15038671894856	-14.71408828072476	-16.87655588056221
C	-3.66281287112173	-13.20268062360399	-16.61878664018160
C	-3.53749221092508	-11.88082031366302	-16.01290064780943
C	-2.38985586031674	-14.02365598677296	-16.37993721117509
H	-1.54611969874128	-13.52316725669221	-16.85245916032498
H	-2.50981318157781	-15.00875157423476	-16.82361368912832
H	-2.19503325898755	-14.12732261550525	-15.31534702036018
C	-4.31826527422301	-10.90084408594720	-20.35600761659279
C	-3.95300787160287	-13.29123181777171	-20.62270801841696
H	-3.97263837593348	-13.20675756133702	-21.70678013996296
H	-4.96105080952171	-13.48075814994983	-20.25717526385959
H	-3.31166493708396	-14.12102413517675	-20.33485062811742
C	-3.41288520502941	-11.99328102097192	-20.02102225757507
C	-1.98267433382608	-11.69556142818230	-20.51099560987128
H	-1.34757292788029	-12.53257968159620	-20.21285934945080
H	-1.98642103823459	-11.64672107806741	-21.60102662719031
C	-1.43301411175700	-10.39243964401645	-19.93044183522468
H	-1.98782854334252	-9.53702742503964	-20.33137196052814
H	-1.53797264086941	-10.36759313377745	-18.84181013714423
C	0.02088102142868	-10.15624854245981	-20.26647228012323
O	0.44064586588088	-10.86008007653896	-21.31480758901729
H	1.36933667050126	-10.69233926869738	-21.51873310451188
O	-2.01788322745685	-2.38280130536836	-4.76067037194254
H	-1.65323719383709	-3.19164647170772	-4.33106959114485

O	-0.40520040093041	-2.71873871486755	-6.26343921867226
N	-3.18823741208364	-0.68362423003554	-9.60707768578277
N	-4.22398717856730	-2.24703344482698	-6.89028079776420
C	-1.29595191177249	-2.03742487189563	-5.81270992996426
C	-1.64394411369719	-0.68255565359628	-6.37452555444070
H	-1.34341568496466	-0.65906778549083	-7.42990954332384
H	-0.98736059117351	0.01533729741633	-5.84448857296480
C	-3.08363727274177	-0.21511822935642	-6.17144063378667
H	-3.11268198408907	0.87323844554975	-6.27375861748104
H	-3.42383807585160	-0.45527892127350	-5.16320356484972
C	-4.09595505205931	-0.80368999248846	-7.16809541390176
C	-3.58266532070635	-0.72250035308069	-8.53227113536589
C	-5.42628653477095	-0.05815567176429	-7.08976473919611
H	-5.26827804524001	1.00342464356355	-7.26901605476092
H	-5.85814554339687	-0.18371036765382	-6.10130057638126
H	-6.12611996271469	-0.44468640949368	-7.83035804382713
O	-3.18413337936411	-3.08632380580078	-2.20042391041798
O	-4.30817196568643	-5.01027782216021	-2.21228169790465
H	-3.68351642657268	-5.24644763639003	-1.46592474328284
N	-4.45299912811819	-5.00257230370837	-8.41786948862631
N	-5.36871201713653	-2.56437283219136	-6.51806453519781
C	-4.04800963051844	-3.79846552179949	-2.66121416947989
C	-4.94157313173759	-3.41447650718871	-3.82311766496831
H	-5.97772943405726	-3.53334648531590	-3.50377762030672
H	-4.77001903410468	-2.36935451485343	-4.08850313892361
C	-4.63388013248259	-4.33883749449027	-5.00451578382410
H	-3.57742747494470	-4.23999454901990	-5.26982744136028
H	-4.81671863910435	-5.37541144335671	-4.71487070371209
C	-5.48123287193599	-4.01210836495969	-6.24446928453737
C	-4.92709538635974	-4.65565888541841	-7.43328313860556
C	-6.94622397813220	-4.38580148131325	-6.04506156200727
H	-7.38238159681653	-3.76731125433292	-5.26486115721225
H	-7.51379923827611	-4.23557753804926	-6.96216914641605
H	-7.02420959645226	-5.43119534748473	-5.75256937059210
O	-5.59008043932292	-0.13064314951441	-3.82472220030687
O	-6.77574988834896	1.28347732518536	-2.56991715461100

H	-7.53516101478539	1.09537489831513	-3.17334460019181
O	2.86838913011700	0.62571371228715	-0.40669701928530
N	-1.74992942160527	1.82251015027090	-4.38890609795928
N	-1.03540054185936	-0.50201707368452	-2.06305268486764
N	-1.09936380756552	-1.09173047205721	-0.97207167474555
N	-0.00586915080918	-3.45998544225332	-3.13713804079534
C	-5.66655249154975	0.65753023414602	-2.91161143745875
C	-4.49751996439571	1.09820001207603	-2.05862771331379
H	-4.86312513258227	1.37810388715204	-1.07200274772568
H	-4.12834833756229	2.01348262800157	-2.53212916964402
C	-3.39645250253841	0.04068341220172	-1.97620757564485
H	-3.51585901409975	-0.69228407407568	-2.78257971639719
H	-3.45758903487041	-0.50046908197813	-1.03047425838201
C	-1.98043317607188	0.63795140702554	-2.07929420461276
C	-1.83726799599069	1.31813307617433	-3.36139372960044
C	-1.64973338055066	1.58999193496875	-0.92960575074271
H	-2.30328352073813	2.45774665553795	-0.95381201828034
H	-0.61568075447611	1.92082555987926	-1.00651999220685
H	-1.78459816221292	1.06679675310169	0.01382929913017
C	-0.08583915218247	-2.94300238860292	-2.11448977993341
C	-0.76565952840697	-3.15566238834358	0.23236046145312
H	-1.73767127645855	-3.53092091217995	-0.08765861763319
H	-0.89139061280866	-2.58879673269085	1.15110116762082
H	-0.09918783324019	-3.99425165355819	0.41652791070283
C	-0.18887084526926	-2.23996330859184	-0.84553310506865
C	1.20659569964912	-1.68546723317965	-0.49715756117508
H	1.87149361200114	-2.51917634032206	-0.26499781187286
H	1.59831054784388	-1.16983986882905	-1.37897944815758
C	1.16536600998865	-0.70174077536255	0.67265719325515
H	0.25214778590929	-0.10066053352386	0.62716332839771
H	1.17548503528692	-1.21011638213574	1.63580335185060
C	2.31751549094795	0.27760637566365	0.60768355746899
O	2.63254205810242	0.73775447252296	1.81061589050313
H	3.36605430277884	1.36477606243402	1.79759250859975
O	-8.19000553162593	-13.05865452227509	-21.40264806639538
H	-7.90943542278253	-13.94075730836047	-21.12280854790489

O	-7.07781867221527	-12.30701954900938	-19.61815229476386
N	-8.12911192759783	-6.53278423294309	-18.54748448685863
N	-7.53173374876117	-8.03673898564114	-21.54263451978490
C	-7.77174743717555	-12.10039924412610	-20.58194830408184
C	-8.25893039179620	-10.74454509882698	-21.03698448584667
H	-9.26923726447762	-10.85476708126385	-21.43154011364879
H	-7.61260025697396	-10.44281472373251	-21.86730039352728
C	-8.19215384760027	-9.70327643916344	-19.92019683947571
H	-7.18603323452677	-9.71047275827114	-19.48818405615622
H	-8.90915147924155	-9.94795726003281	-19.13409634239902
C	-8.48429588173079	-8.28185083840664	-20.44684293527817
C	-8.28573754367724	-7.31160530195576	-19.37521041546250
C	-9.89584885684154	-8.12462411012672	-21.01621252228098
H	-10.63658157408340	-8.41363211238422	-20.27462531874686
H	-10.00560990578428	-8.74926237101730	-21.89887921817922
H	-10.06135890939770	-7.08807479633513	-21.30316305130549
O	-5.52239297693691	-10.61929300147883	-25.21815337788679
O	-4.56407275511565	-9.31465282200915	-26.75272439951749
H	-4.70482169148107	-10.05268016176051	-27.35829680819887
N	-3.05119664641610	-7.84394707580713	-21.43732731848321
N	-6.40618454866293	-7.68049069529587	-21.15674061698439
C	-5.01438703704344	-9.57355520067563	-25.53123419505114
C	-4.79723798485284	-8.41069954075538	-24.58598116995135
H	-3.76829748949455	-8.48508100646421	-24.22179996343392
H	-4.88647726322724	-7.47813117155628	-25.14386316167584
C	-5.78971646178185	-8.48341427646432	-23.42691570493681
H	-6.79858351659797	-8.29146464767054	-23.79621256908672
H	-5.77480384257749	-9.49084447756649	-22.99946846753313
C	-5.48596621053069	-7.48445354882172	-22.29870817563141
C	-4.12943010487256	-7.69201548509406	-21.80827686825880
C	-5.65762263259888	-6.01921178078948	-22.72185815227038
H	-5.48355505874147	-5.36651076540285	-21.86657176279327
H	-4.95488259538081	-5.76785512988220	-23.51193574965460
H	-6.67420823650674	-5.87764835739143	-23.08167545426929
O	4.87462997912507	-10.49697863573992	-6.84581877906614
O	4.16532474475346	-12.36506897233328	-7.83466668803764

H	4.45173975426728	-12.80455678847319	-7.02353885367943
O	2.75221678964069	-3.29518017367229	-4.86416729708050
N	-0.04234455164295	-8.10904659270184	-9.59495308200797
N	2.79275353302234	-8.24632787281374	-8.03587616985140
N	2.85873524737798	-8.00069385414562	-6.82235991229644
N	2.81027542038465	-4.82003419228864	-7.74858128800881
C	4.42461820959570	-11.06058695473007	-7.81269072917812
C	4.09691351840953	-10.39432273135835	-9.12688855769125
H	4.51438282853500	-11.00448657393024	-9.93016153885207
H	4.56009890692147	-9.40567243088066	-9.15255221590444
C	2.58701657235812	-10.27981836716617	-9.36310240146242
H	2.13116278468159	-11.27027835473126	-9.33131327189217
H	2.44652562388794	-9.87243809308862	-10.36792523725509
C	1.87356804685073	-9.35963931062748	-8.34852026310289
C	0.76646387894867	-8.69850798146014	-9.03521498873531
C	1.37562244448729	-10.09015413697819	-7.10441058424772
H	2.21953497864996	-10.49131762233534	-6.54957772393235
H	0.71636660160514	-10.90473519207092	-7.39573441929736
H	0.82777865630535	-9.40822882209807	-6.45416899886813
C	3.25894444292063	-5.71457949773189	-7.18877266882340
C	5.17863390005922	-7.21870810046858	-7.09381181660342
H	5.54625263944692	-8.13595070376696	-6.64020034816630
H	5.86772762319064	-6.40552002633782	-6.87660415621466
H	5.11357103395236	-7.36364992159128	-8.17157413928055
C	3.79603181549087	-6.89441855555786	-6.52420719557497
C	3.83276626587982	-6.73058073861879	-4.99528247464235
H	2.89464159815301	-7.10340233586151	-4.57732409860792
H	4.63346818080195	-7.35659627289584	-4.60243636178251
C	4.06110161799805	-5.28370873007765	-4.55346697707491
H	4.43155997579232	-5.28698232209394	-3.52649237514812
H	4.80719300094994	-4.78312336120532	-5.17362277284310
C	2.79679696784042	-4.46000553184698	-4.51016489591730
O	1.76979387753510	-5.09990870987965	-4.00474957417161
H	1.01150025221892	-4.45755355823247	-3.74595848063263
O	0.65934496615307	8.57798483767176	-6.45375170268168
H	0.28689852835990	8.17137086196065	-5.61844990657118

O	-1.48689593355254	8.69407337145487	-7.01544246774081
N	1.51275940346773	6.00180955619207	-8.04056480466746
N	1.33241139660928	7.90200262980909	-10.69840185889635
C	-0.30871870673859	8.87839238441939	-7.28151199466054
C	0.11809691973728	9.49228827695281	-8.58571434250870
H	-0.21497700115947	10.53130738321521	-8.55417523555191
H	-0.44547086035667	9.01432387571567	-9.39394185245017
C	1.62060150380831	9.44705153745505	-8.85557097331236
H	2.17879398520358	9.66025777121357	-7.94290111369648
H	1.86508015053806	10.21987868408386	-9.58586322442031
C	2.09503359563796	8.11185772351713	-9.44716385329883
C	1.78556225031674	6.95981065236718	-8.61007074898015
C	3.59498188875772	8.16640202717255	-9.74939617704999
H	3.80074629701025	9.03272031891946	-10.37340473115187
H	3.90783987220905	7.26828228489383	-10.27801307776345
H	4.15618410156127	8.24730693361285	-8.82214676688459
O	-1.19834230307294	10.05405435494176	-11.46435698830284
O	-0.27849600914280	11.50660122824493	-12.88258514516237
H	-0.83193366603027	12.17000225563813	-12.44966206486655
N	0.07101311997886	4.43476405475804	-12.52660417137365
N	1.79118300021766	6.98285321965784	-11.39187446642409
C	-0.43627159080797	10.29290442596375	-12.36828823835179
C	0.49410857635089	9.29194190571674	-13.01644817421778
H	0.67597145129366	9.58740586274213	-14.04885163965159
H	1.43877230554135	9.39907848791244	-12.47513891683674
C	-0.02238564921670	7.85239291528738	-12.91817034141801
H	-0.73131337084184	7.77924281112757	-12.08879586590103
H	-0.54296973001017	7.58492065240732	-13.84117615843524
C	1.09661571397770	6.82492182686080	-12.68588461653252
C	0.51399409875106	5.48701262537069	-12.63185525075536
C	2.19975049525985	6.88243335160419	-13.74704814893944
H	1.77235033993288	6.75859710824654	-14.73819515904285
H	2.69942240445543	7.84555926016959	-13.69309209292968
H	2.93552043055250	6.10127053894248	-13.56579816734429
O	-0.19401877615540	5.77666534688447	-15.73433030578082
O	0.39676122204631	6.87978689594319	-17.58481252632081

H	0.27072140066536	7.69400439426532	-17.07580963867497
O	1.78975498706746	-1.45553025130344	-15.77787870381396
N	-1.24650519300052	0.05696808361806	-16.77779155252430
N	1.51630352405856	1.99684803082806	-16.85417750491117
N	1.37166769485660	1.64825778096060	-15.67096149359732
N	2.21445665536813	0.52425495724911	-12.67567435979153
C	0.16933286756921	5.77779401051387	-16.88478848742501
C	0.48162164582023	4.53933682040244	-17.69428139931696
H	1.53194856511963	4.31006612685942	-17.49031375077711
H	0.39445401763435	4.77277752318144	-18.75436771800499
C	-0.40215510145677	3.35582965132596	-17.29889190104093
H	-1.34709001877976	3.41109636481100	-17.83533384782705
H	-0.60389646663814	3.39647071312120	-16.22231338770456
C	0.26342909516000	2.00428913703261	-17.61897875432405
C	-0.60171780929783	0.92040280019686	-17.16851522967205
C	0.59837835890497	1.83940276825305	-19.09896851010073
H	1.04445626799681	0.86304732851726	-19.27609374403227
H	1.31036431874782	2.60275967961932	-19.39848042864319
H	-0.30197453530670	1.93635108065012	-19.70093768751114
C	2.43537200460531	1.08765111062462	-13.64933393150317
C	2.78620166710294	3.30362735755243	-14.65178619044766
H	3.67092769926093	3.48952857369774	-14.04864173016701
H	1.90617094518121	3.70078486982372	-14.14627245733937
H	2.89943785782039	3.80732336147750	-15.60812968729046
C	2.62866108996203	1.80130761122663	-14.91023545250356
C	3.83110140446183	1.26748225030060	-15.70274671513758
H	3.78412038079970	1.69368092093624	-16.70608858118681
H	4.74803220427517	1.61635905711542	-15.22804553592191
C	3.87237820962504	-0.26000026076308	-15.78950951540260
H	4.72184690407501	-0.54198825618339	-16.41091289285202
H	4.00588627387883	-0.68535055272548	-14.79248943482368
C	2.62066392014415	-0.84391537221643	-16.41176283050749
O	2.54493407975011	-0.63681217851734	-17.70903708097039
H	1.72316219805922	-1.07772434206005	-18.10692445827874
O	-11.50040576766587	-4.77691352182954	-7.59785842487728
H	-11.45902135513894	-5.69825113388177	-7.94786688154094

O	-9.90515039685781	-4.24800564473836	-9.07318820102649
N	-7.13304888879776	-2.87374152420788	-8.73182347785448
N	-8.49793577294443	-0.03987947874167	-9.28199328183375
C	-10.74862771138721	-3.92445079444101	-8.27009796973460
C	-11.11041166741293	-2.49525106464950	-7.94629818587306
H	-10.93568335960260	-2.31766009647650	-6.88458230021122
H	-12.18769208189076	-2.41083954837985	-8.09915604049268
C	-10.39708464426114	-1.47806487207888	-8.83227854984657
H	-10.98134566309208	-0.55706491870503	-8.84186767554329
H	-10.34296149840592	-1.85079377911276	-9.86123825839843
C	-8.98594273146750	-1.09322382098645	-8.36460602690414
C	-8.00626440541854	-2.16201281999145	-8.51714218911344
C	-8.99494941303374	-0.51485077890293	-6.94871414732176
H	-9.87075563029479	0.11586415708474	-6.81813144898162
H	-8.11280906958952	0.09985501392700	-6.77947012994182
H	-9.02024868992240	-1.30654433093500	-6.20348427069328
O	-9.52309248385460	-2.24535040595206	-14.99416056543074
O	-9.98150317618237	-0.06692604638778	-14.91470663045251
H	-10.65738699926208	-0.27111512032554	-15.59927248872144
N	-5.34621374503584	-0.06997049033216	-11.62094403798621
N	-8.35533644652253	-0.45306110216256	-10.44577683586243
C	-9.36968950602565	-1.15813955877342	-14.48370930767589
C	-8.47842897532167	-0.93817707371122	-13.28233454220531
H	-8.78160324228523	-1.68517933558564	-12.53781587143860
H	-7.44878762375714	-1.17784081520919	-13.58271169917637
C	-8.57913182612013	0.46849518934681	-12.70232853906676
H	-8.16127266532639	1.19833000788085	-13.40068332660744
H	-9.62568733904436	0.72862125271742	-12.54505978407597
C	-7.85020798211938	0.59869369348365	-11.35635819723355
C	-6.43940769744410	0.25901244518430	-11.52170040936000
C	-8.02736083474218	1.99276721903769	-10.76471084269016
H	-9.06874345169404	2.12391097482338	-10.47845210663876
H	-7.40666123482827	2.12988875692181	-9.87922452766843
H	-7.76620906906415	2.74242813858610	-11.50751458425914
O	-0.39353975893536	6.95680021607253	-4.64948623155370
O	-2.32839430705098	6.53555807279226	-5.66916140227953

H	-2.14630158052301	7.42073981810747	-6.10301684007737
O	6.37718472296166	0.08110426437014	-5.80919100619535
N	-0.45702982321742	4.02220691732101	-7.05332884382132
N	1.10108763665257	2.56240431944278	-4.47364901882030
N	1.75758032328065	2.07456097667773	-5.40796012510671
N	1.58490744704915	-0.57599031081527	-7.26627803026665
C	-1.36985098703968	6.25000321365360	-4.82228110965153
C	-1.57316292760526	4.96648082184049	-4.05302611237946
H	-2.14356597553238	4.24596651037432	-4.64734504122690
H	-2.17687990924275	5.22208536263981	-3.17960158303128
C	-0.23239841877031	4.40351109164831	-3.57805584607678
H	-0.41127219837942	3.54211548429497	-2.93273055421731
H	0.26804602187961	5.16541062779613	-2.97987557619983
C	0.70615628625424	3.96381939861835	-4.72447205898917
C	0.04855894001454	4.02009083591074	-6.02450883419730
C	1.99490998063549	4.79210869343988	-4.76547135866352
H	2.50025170052695	4.71291611109031	-3.80643300906019
H	1.76201695915035	5.83539638271767	-4.96386152455399
H	2.65372079040071	4.40998623773351	-5.54499475879111
C	1.92112750923957	-0.05788263407491	-6.30144780862166
C	1.60845407197438	0.09784628212738	-3.86316365184663
H	0.52151987893929	0.15892309506230	-3.91963565448330
H	1.93472186990130	0.63619712869037	-2.97614699936217
H	1.89849631474041	-0.94828942751280	-3.77466545195686
C	2.25233376320618	0.71240116695464	-5.10440051177839
C	3.77300591569272	0.90450416290259	-4.94501546414576
H	4.20128028491962	1.15384038894272	-5.92022502236205
H	3.92440735998436	1.76525675093527	-4.29074131509494
C	4.49175370100569	-0.30978775991962	-4.35488486233752
H	4.42253253379234	-0.32452293318686	-3.26706182669908
H	4.05948612435052	-1.24557258575934	-4.72414326064943
C	5.94928869450391	-0.33459249072415	-4.75899057745227
O	6.71434439667900	-0.89800473710980	-3.83604223723975
H	7.65051376914636	-0.87555094502104	-4.07100849884584
O	4.05111096893866	-2.17387239487102	-6.96364342451053
H	3.66642119001012	-2.76913348237055	-6.26331699026974

O	5.54030093136365	-3.80958481409167	-7.22759068200472
N	2.72358530809660	-2.45069940866351	-9.99421885701669
N	3.45166118821307	0.74864056543980	-9.60001643535048
C	5.15963878820492	-2.68397023911222	-7.46452121943469
C	5.95778730515752	-1.72696174071064	-8.32022520146854
H	6.29349150122429	-2.24430487621757	-9.21963565396377
H	6.85356513945711	-1.51671655134253	-7.73138592587682
C	5.27754990875776	-0.39806401419354	-8.63253739417818
H	6.04330495170545	0.35076689203596	-8.83778816681008
H	4.72135257635698	-0.05612174397361	-7.75555337086440
C	4.32925734592041	-0.40965520488199	-9.84810803808596
C	3.45749513437499	-1.57718176264646	-9.88962061303824
C	5.09053763075887	-0.30793076335059	-11.17004644571416
H	5.69365261712899	-1.20018417753617	-11.31880788858249
H	5.74466369991929	0.55954023568054	-11.14871408623054
H	4.39481268991719	-0.19883011862363	-12.00303715390682
O	4.83352910439643	5.72733038059182	-8.50495616023678
O	6.77744944987569	4.63776065550122	-8.36599371783355
H	7.19639075476828	5.49749299207503	-8.49425722153088
N	0.57179557476139	2.32506630990813	-10.22788191395856
N	3.84293775887392	1.76580282107825	-10.19675019318520
C	5.45304634057293	4.70545435881118	-8.34185869189693
C	4.83605484115259	3.35571844990500	-8.04755582754521
H	5.39442036621132	2.58587119577072	-8.58464655131073
H	4.99678654171893	3.18537735666878	-6.97908525779373
C	3.34005343835008	3.28350753931286	-8.35616824517099
H	2.89140130661753	2.49063121089778	-7.74629394900574
H	2.86269960391892	4.23137753834545	-8.09190795259536
C	3.06218389387599	2.97165710978032	-9.83974791404539
C	1.65246861460896	2.65266340086076	-10.03411096418225
C	3.48170328415811	4.08627596294513	-10.79287107449058
H	4.56131773636517	4.20017691296652	-10.76639354533713
H	3.18833186782290	3.83629345872358	-11.81135755647628
H	3.01878064889819	5.03123901872343	-10.51465451248609
O	-4.11085150183740	-5.53992913463389	-19.86595811160393
O	-1.92931347319638	-5.76452664918765	-20.28721703951771

H	-2.21454556366349	-6.63582096822212	-20.68877400151197
O	-4.89359674872838	3.31608494569496	-17.82383854095283
N	0.31824860799956	-1.67501092676210	-18.67834294394626
N	-2.77465411944441	-0.85065330269727	-19.70256225014754
N	-3.27954686289722	0.06798850301478	-20.37074528225586
N	-4.31445179906689	0.44368710503192	-17.19054118059612
C	-2.99233851288245	-5.07830287706039	-19.90293145707521
C	-2.65602717703229	-3.65298897296386	-19.53538484216599
H	-1.89500086814912	-3.67132278507645	-18.74762840281159
H	-3.55272439685110	-3.15896063632188	-19.15603367185405
C	-2.11779915327099	-2.90587216340026	-20.75969238000694
H	-2.90371014307964	-2.80569060735495	-21.50974271634382
H	-1.28789332643546	-3.45589301700984	-21.20607101214486
C	-1.63582667309404	-1.48561961882708	-20.39095234877963
C	-0.53992540333493	-1.58086255498613	-19.43527607985900
C	-1.17825929167032	-0.71483138298098	-21.62808164663759
H	-0.31124945275743	-1.20127637617120	-22.06660902147288
H	-0.92352190250392	0.30761770963444	-21.35850597628786
H	-1.98525390636097	-0.69023511460253	-22.35554417426098
C	-4.42873457267104	0.54613369135938	-18.32519126448697
C	-5.63986887993597	-0.36882933324324	-20.23656670654572
H	-5.72673679981549	-0.32549040264175	-21.31880428047002
H	-6.58781411923267	-0.07773578013446	-19.78807367387817
H	-5.39320545967608	-1.38943267938062	-19.94481864722063
C	-4.52822627404013	0.58646807323801	-19.77815383786271
C	-4.77829388217123	1.99410527198052	-20.33340880834292
H	-4.77930822876394	1.91172936612115	-21.42040214163621
H	-5.76789863616523	2.33125370152435	-20.01928431771804
C	-3.72795935618241	3.02419989998705	-19.91875717702094
H	-2.72843138756406	2.58033056111704	-19.90113358386613
H	-3.69943132819859	3.83511392934484	-20.64787403321355
C	-4.00319822969367	3.65345671104812	-18.57054837192487
O	-3.15551859032436	4.62732301406339	-18.29873888287812
H	-3.35395229027624	5.04138151383256	-17.39211008507565
O	-0.66069790665235	-4.82910519756013	-16.71997300095662
H	-1.47649512019500	-5.39307006654238	-16.95014206591880

O	0.27155925614825	-6.81952580621277	-16.37223992318338
N	0.82140269491752	-2.93068324954770	-13.26776540013265
N	3.22571965108672	-5.12662069208592	-12.78569738472876
C	0.35237656541591	-5.60283520926962	-16.43754909899770
C	1.65055968137818	-4.86935880050004	-16.20283063251235
H	1.45264344608379	-3.79949462272986	-16.09323134721978
H	2.23965179436624	-4.99879199940340	-17.11210796710158
C	2.41223828324281	-5.45385736395730	-15.00230477335227
H	3.30172835064029	-5.98943910987786	-15.33531839027905
H	1.78603742151544	-6.17891807179505	-14.47164053216221
C	2.86423871252388	-4.37032993288981	-14.00325426284357
C	1.72975917631980	-3.53221976804198	-13.62386668548374
C	4.02713207747044	-3.54911925735605	-14.54683482190682
H	3.74388498354348	-3.08150295951474	-15.48517278709921
H	4.87515593203370	-4.20483099620868	-14.72743794886179
H	4.33208277757093	-2.78582125397131	-13.83237793556379
O	3.82368218004316	-8.16465332233905	-13.81244943296031
O	5.19183086333751	-9.25246790829680	-12.41707367725627
H	4.52883944043322	-9.98675727018512	-12.43905784182487
N	2.90686749762613	-7.42872485812017	-10.73024370844164
N	4.41334347072854	-4.96120329046899	-12.45976419986168
C	4.85850661894972	-8.23722254603116	-13.19149928137542
C	5.92500856636853	-7.16652420295250	-13.20618081646071
H	6.86390912871792	-7.63158214487006	-13.50574375327904
H	5.65800036080122	-6.40047618506283	-13.93634741709353
C	6.11216559587596	-6.54168523385732	-11.81982434902226
H	6.37456393645456	-7.31264075124798	-11.09377670354961
H	6.93148818121802	-5.82414727525200	-11.87016104227091
C	4.85018165957020	-5.80121458509619	-11.34102510989625
C	3.79201999378210	-6.73984871451747	-10.97317532421999
C	5.18850462593401	-4.86236561568880	-10.17993640073954
H	4.28792318190731	-4.37654168222063	-9.79939549775903
H	5.66435291764360	-5.41519010535996	-9.37458272352635
H	5.88094448419307	-4.10340242592145	-10.53821731835913
O	-6.57257258941257	-10.99165076583438	-11.93579008691951
O	-5.77671643258662	-11.71364619600303	-13.89100096535219

H	-6.64441750671386	-12.15750715091473	-14.00022538337306
O	3.36680515422277	-11.27092002903523	-12.25613039334617
N	-2.99513358994480	-7.62766385286118	-9.03866422788127
N	-2.01196633671958	-9.02974873134182	-11.84202607292875
N	-0.90409152784892	-9.55200146593519	-12.04279240524722
N	-0.10396233039322	-6.43703195552680	-12.99596529127606
C	-5.64718851770743	-11.16779426485529	-12.68889008168338
C	-4.20611712473047	-10.82985424639850	-12.37346723896094
H	-3.74627756553869	-10.34600806129588	-13.23999102345739
H	-3.69261998766455	-11.78211882382212	-12.22539791193431
C	-4.13687503313461	-9.95876717057573	-11.12532921867102
H	-4.64144081618625	-10.46839903765880	-10.30200945673494
H	-4.67032942469460	-9.02321611596843	-11.32553394847464
C	-2.70244148319142	-9.62501810704509	-10.68138478424160
C	-2.82749321700016	-8.53741646788644	-9.71439843498223
C	-1.96257063973001	-10.81846486402298	-10.08767416231008
H	-0.97966954771246	-10.51983167221767	-9.72647077198412
H	-1.83269502106717	-11.58557151551942	-10.84659661928961
H	-2.53239335593804	-11.23021966325494	-9.25838263935120
C	-0.12096527955054	-7.57760993246123	-13.11822614553748
C	-0.99557208501395	-9.40909733884386	-14.47699920732394
H	-1.19579374754715	-10.47672344231804	-14.46918572472811
H	-0.42628445295872	-9.16324931848378	-15.37083188091824
H	-1.94800990986393	-8.87791832487536	-14.49308845674816
C	-0.19743529698074	-9.02754823541606	-13.22859035688897
C	1.20769367292191	-9.64525720045814	-13.22590327767624
H	1.74188331905427	-9.34635088736053	-14.12961970575219
H	1.76804315310821	-9.26884117899100	-12.36384106979418
C	1.12340624630051	-11.16969044609265	-13.15580887541860
H	0.42910652639175	-11.46889472513884	-12.36247684385408
H	0.75416281147990	-11.60116327001528	-14.08688902940254
C	2.44461608495278	-11.81446961138683	-12.82660186641592
O	2.48235130539531	-13.07849305560715	-13.20311643079220
H	3.31735163974205	-13.50741383738705	-12.97823709582206
O	-1.66285581025720	-8.21820946189633	-17.45357511740982
H	-0.85198052701104	-7.69674936946302	-17.13888041993994

O	-2.76550938135869	-6.30870334485702	-17.13075342882716
N	-3.65638379787540	-7.40241675755589	-14.43933839078092
N	-6.69633602103960	-6.78563389706063	-15.64023107536602
C	-2.74466939149587	-7.48621527596454	-17.45317732455204
C	-3.96541212057354	-8.23135991018331	-17.92281765495841
H	-3.90952486315773	-9.27079612901613	-17.59358032203628
H	-3.90504927932093	-8.23831049341001	-19.01514003997046
C	-5.26848367166803	-7.54962980897503	-17.52311064026839
H	-6.07263747896118	-7.93844000103644	-18.14760888817077
H	-5.18430986439424	-6.47567637210491	-17.71880678695770
C	-5.66772239863336	-7.77692877482631	-16.05094025256079
C	-4.53582744372037	-7.58595626123527	-15.15241980721793
C	-6.29298190892240	-9.15670855997618	-15.84551210500333
H	-5.60328574060765	-9.93965143972649	-16.14919392117537
H	-7.19565091513408	-9.22929803794619	-16.44747878755271
H	-6.55542736790618	-9.30853371681653	-14.79996981188043
O	-5.66323450623443	-1.47302408627138	-15.29965610948579
O	-7.03825969448798	-0.74159724742403	-16.90284943953748
H	-7.07369299685257	0.13902547693727	-16.41316528074313
N	-6.70663194177154	-3.57493303804533	-13.55231757148322
N	-6.46521810722829	-5.63835206309259	-16.04352245595088
C	-6.25856941309210	-1.64424987790436	-16.34021411392804
C	-6.23511676116810	-2.91963643641163	-17.15406499753867
H	-6.14928152955588	-2.63719482849437	-18.20353397841406
H	-5.37779909826026	-3.54117163124935	-16.88866572329324
C	-7.54087915714381	-3.70148056944669	-16.97500215979072
H	-8.38096852984353	-3.01037837996413	-16.89093277962290
H	-7.70954499994294	-4.32039882180399	-17.85776372508449
C	-7.53758567952752	-4.65715098392608	-15.76761913767676
C	-7.11486377466314	-4.01340765824924	-14.52920749826905
C	-8.91656051494930	-5.29959058859256	-15.61484134721038
H	-9.00907603652082	-6.12130981208483	-16.32083175076359
H	-9.08864437902980	-5.66876045222891	-14.60663041911943
H	-9.67701756192597	-4.55819761382845	-15.84180680493958
O	-1.84101963798489	7.05640715450149	-10.10708650714356
O	-3.25678682662802	7.34589745842343	-8.40583047957277

H	-2.98277407675491	8.28071067742301	-8.43405848940085
O	-6.01483132402610	2.33700570816730	-13.17830370408534
N	-1.47046645505604	1.63010607131286	-8.12936470074159
N	-2.22425426483138	2.15153941612910	-11.23548227067162
N	-3.08400181313405	1.68060212325494	-11.99524138925349
N	-1.82738556579262	-1.29634769977388	-12.30302320345828
C	-2.58343378687940	6.60047069259666	-9.26549986081518
C	-2.85668952358013	5.13490907769637	-9.03926884596122
H	-3.92043098503301	5.03607270185563	-8.81058307102455
H	-2.30312031836214	4.84585184669212	-8.13600445462830
C	-2.44659989059713	4.28547394897852	-10.23443859658600
H	-1.36973670605529	4.38141192618819	-10.41360414761360
H	-2.96405434247731	4.63922255873622	-11.12999226729884
C	-2.78739246102493	2.79829329724508	-10.04656019261954
C	-2.04906885678116	2.20302529147755	-8.93653099847828
C	-4.27930389744258	2.52488776717349	-9.87149763607466
H	-4.63450182757522	2.95823609577397	-8.94108591793846
H	-4.46853319402594	1.44939730835549	-9.85672457572357
H	-4.83531553687290	2.96359228047866	-10.69460080798264
C	-2.11201106921037	-0.27820422277241	-12.74518308742727
C	-1.21066501192847	1.81079531108877	-13.62170520316312
H	-0.43858492474027	1.74713923383104	-12.85238160557669
H	-1.43879823842103	2.86266689341466	-13.78039612436914
H	-0.82170198521147	1.39303511270209	-14.54745661619273
C	-2.47386657949145	1.06311825524331	-13.19385322986456
C	-3.54186182044715	0.99974629800881	-14.29262161307396
H	-3.09324704895325	0.57339833763482	-15.19130573547294
H	-4.36259950474399	0.34239122057846	-13.97668219940719
C	-4.11113481290029	2.37857241556731	-14.63791625370483
H	-3.32527478119878	3.10177183577044	-14.85756289438321
H	-4.73313762119984	2.27767551418880	-15.53191502276446
C	-5.00405359362501	2.89766654165202	-13.53714074115375
O	-4.58346599791811	4.03455608569776	-13.00762470841262
H	-5.24749503730491	4.38303178260288	-12.35355133334068
O	-3.16789284482174	-3.06853587176339	-16.76287267617840
O	-2.16699676322611	-1.93067056186696	-15.13939053435496

H	-1.85592583132509	-1.36818721438551	-15.89715272549761
O	-1.49632577508431	-5.24260743982941	-4.91767426759170
N	-4.68315585151656	-2.73515578124911	-11.23785067251521
N	-1.75308398938440	-4.14017322386188	-11.22407271759772
N	-1.57782475656577	-4.87026326347988	-10.23213107980637
N	-0.48913116034074	-1.87000892816321	-9.30811174198952
C	-2.92890628221811	-2.91291893117120	-15.58759575177339
C	-3.50094457615080	-3.79644333397990	-14.49657477442195
H	-3.99224413876500	-4.64875808553595	-14.97114880800144
H	-4.27709569384693	-3.19993260501130	-13.99905266664953
C	-2.42771268802415	-4.25101713058097	-13.50353731613460
H	-1.62705105477910	-3.50033141337180	-13.45943790361155
H	-1.97700138795769	-5.18134561239409	-13.86015680150867
C	-2.92073523886840	-4.47062008704685	-12.05676788031216
C	-3.91805919782550	-3.48345858165347	-11.64888789587573
C	-3.48703325924449	-5.86043426479032	-11.78618172910654
H	-4.34363704849624	-6.04266127716533	-12.43345691908598
H	-3.81022045890123	-5.94224128447965	-10.74596272326514
H	-2.72905845069616	-6.61907972890677	-11.98189949251725
C	-0.38033355132637	-3.00918882545225	-9.39010213954261
C	0.81936668312178	-4.89531817874440	-10.43369788057312
H	1.77118072219037	-4.67491152311535	-9.95547232116002
H	0.76617926624001	-4.38059396255754	-11.39241118044504
H	0.75466737562678	-5.96864851188102	-10.60906291689173
C	-0.33995955511146	-4.46217732531067	-9.53338594655022
C	-0.19741956252156	-5.13245799096027	-8.16872365471475
H	0.20031136101405	-6.13808828012750	-8.31760309584531
H	0.54907684675100	-4.57116187148691	-7.60020560358046
C	-1.47653325777938	-5.23522739156133	-7.34202935062922
H	-2.01797177688899	-4.28694125830929	-7.27231489571866
H	-2.14891135853654	-5.98354975936661	-7.77741678100000
C	-1.08982108498597	-5.71278888741784	-5.95690131583865
O	-0.22531549378395	-6.71043930999822	-6.01363579717661
H	0.03023992303075	-7.05579949577751	-5.12992160966025
O	-1.83416964658874	-9.56411815120166	-4.63844939143145
H	-0.91324649934325	-9.20580707835244	-4.63599309703800

O	-1.70795725701920	-9.56828593517603	-6.87015543780202
N	-3.78036500344903	-6.97535276293043	-6.14898592837270
N	-6.09522904592309	-8.70640390067609	-7.95105994271749
C	-2.29249537404509	-9.84642224907906	-5.85006395846981
C	-3.59743816463488	-10.60503061271850	-5.79374195344376
H	-4.06065935290695	-10.47350325733143	-4.81647822253576
H	-3.31807863289715	-11.65761081935272	-5.87893219889636
C	-4.55167023874249	-10.26057871859361	-6.93644704597709
H	-5.26765741715968	-11.07083314511197	-7.06787611810117
H	-3.98264726262457	-10.16262478266727	-7.86688387795209
C	-5.35356051403220	-8.96910720916865	-6.70732945341963
C	-4.47468419548242	-7.85839225318273	-6.37796854312681
C	-6.43487772970780	-9.12063229023330	-5.63189542078441
H	-6.99359310738724	-8.18879674185282	-5.55148286447630
H	-5.99354291455244	-9.36410843911294	-4.66910447828625
H	-7.11644961475047	-9.91505458989955	-5.92452151107487
O	-6.23064456912758	-7.72513273119385	-12.50455682288521
O	-8.18735328442992	-7.54642276158072	-13.55613423343535
H	-7.64412795482840	-7.29521867024875	-14.37927213485160
N	-6.52502297638580	-5.31864131787796	-10.55809945903747
N	-6.48798815161793	-7.53271344658589	-8.06280087443051
C	-7.43493936928561	-7.85140975310540	-12.51464111124195
C	-8.26499922563105	-8.42264339057593	-11.38915975701529
H	-9.17333108922013	-7.82922923690303	-11.27186313905113
H	-8.57758827816329	-9.40538157231486	-11.75104809386181
C	-7.50900809740258	-8.61417401481209	-10.07834240172121
H	-8.00364041243523	-9.38786699828788	-9.49027485017813
H	-6.50242795959414	-8.97649565586036	-10.29879231675642
C	-7.41724103275624	-7.35554301499080	-9.20307818688284
C	-6.93778096070846	-6.20814018156100	-9.96622147066721
C	-8.75751442363165	-7.03835803790895	-8.52972997885899
H	-9.52319967252478	-6.82426259906526	-9.27137209830912
H	-9.06626155455620	-7.89884947320032	-7.94161494645302
H	-8.65693507302802	-6.18030339593379	-7.86724965084506

RR-SS

```
:::::::::::::::::::::::::::::::::::::::::::::::::::::
::                  THERMODYNAMICS                 ::
:::::::::::::::::::::::::::::::::::::::::::::::::::::
:: total free energy       -1371.533474475107 Eh   ::
::.................................................::
:: total energy            -1376.800970323018 Eh   ::
:: zero point energy           5.793181830488 Eh   ::
:: G(RRHO) w/o ZPVE           -0.525685982577 Eh   ::
:: G(RRHO) contrib.            5.267495847911 Eh   ::
:::::::::::::::::::::::::::::::::::::::::::::::::::::
```

O	-9.83930013584465	3.09182579792496	6.24917008083199
O	-8.85551289523835	4.19737737571124	4.58682098097596
H	-9.75192391426178	4.30008814049517	4.23975862330449
O	-1.62921529365758	3.10547671913125	5.68214278801316
N	-5.43643996691322	4.17894789579249	9.36200179183071
N	-4.66670866435138	3.03777464133367	6.23230262640770
N	-4.46996700165601	3.16830948191452	5.01199946709456
N	-3.21933745056590	0.05156385742133	5.83699182800032
C	-8.83580115666680	3.53037240494489	5.72617194180066
C	-7.45367779687786	3.34014922585077	6.29598010469227
H	-7.54359180401609	3.04785868253891	7.34388547321274
H	-7.01097011902267	2.49452928001563	5.76108273230662
C	-6.55361347692234	4.56757710467420	6.12947067561116
H	-6.39678548141499	4.76153668828567	5.06753731614631
H	-7.01637193413693	5.44710443284873	6.57952142995432
C	-5.17590242351562	4.32128641617226	6.77421767502620
C	-5.32106279178423	4.21909943414233	8.22043787604170
C	-4.18126371188736	5.44042387550405	6.44924968840517
H	-4.52614523849519	6.38233016700799	6.86808830959677
H	-3.20059336412959	5.19599745838981	6.85778206973543
H	-4.09760663615512	5.53461345195361	5.36918791559354
C	-3.60286177656169	0.90055979083102	5.16372657421081
C	-5.37535421760451	1.50707473654547	3.59437368133820
H	-6.11561252250409	1.21395186945894	4.33636205284386

H	-5.76728977853521	2.33297143330047	3.00697473985400
H	-5.16944849977764	0.66529840993890	2.93916751252596
C	-4.08125287100322	1.96113725904213	4.28853685648598
C	-3.02712199727046	2.37147471937840	3.23303623471940
H	-3.09344980295175	3.45446352175761	3.10897287995127
H	-3.29418570238198	1.90780137858649	2.28280835950446
C	-1.58714696070579	1.97001101983089	3.54398170565796
H	-0.95421616264078	2.23879263341075	2.69708355944744
H	-1.51705683264395	0.88424815290099	3.65821225098074
C	-0.99302288223160	2.60530366592987	4.78310433255075
O	0.32499561218666	2.52817024840145	4.77193498518982
H	0.73758877027028	3.00487192054246	5.56291943806902
O	-5.96624023359984	1.81957494582556	11.68754448254340
O	-5.79315802555242	1.35596662978744	9.51136109886073
H	-6.74269753418371	1.62620020956682	9.44486842325734
O	-2.18514694876151	7.31084364029892	11.77570368398121
N	-0.79383538785651	0.38732142665471	11.65164151101456
N	-0.81107923926917	2.87903511583767	9.17915876970587
N	0.14328569995266	3.14707523237939	9.92999738393000
N	1.34551004680598	3.67865401508778	6.91914621335348
C	-5.29467550292008	1.55667488086187	10.72003034234724
C	-3.79404498247136	1.35807856083595	10.77488062231966
H	-3.60620375385982	0.28970668259339	10.62686049621285
H	-3.44368436728739	1.63766159438255	11.77428949672105
C	-3.05171761766808	2.15206729655560	9.69925125234096
H	-3.04885131392191	3.21088378994064	9.97076800580740
H	-3.55996440903188	2.05991656805453	8.73631426634252
C	-1.58498045442969	1.68390563557527	9.53508403837174
C	-1.10300485317584	1.00570397468382	10.73642630251359
C	-1.39420792018626	0.74323925635464	8.33663121535180
H	-0.35262302489461	0.42880992700969	8.28144133363572
H	-1.64199792550089	1.28259922168936	7.42560366760786
H	-2.03285608893865	-0.13591395500723	8.41579498753467
C	1.16126380861623	4.00269591483432	8.00510085389075
C	2.19441095644798	4.46207060626571	10.17504670014192
H	1.99581811119219	4.54698351570805	11.24114948801049

H	2.72397575351427	5.34990819670411	9.84102929438154
H	2.82306122006190	3.59032479110762	10.00176608039321
C	0.88539685683354	4.31256422280178	9.40531390305867
C	-0.01650606647171	5.55926142315938	9.47573996533208
H	0.56628656748889	6.42469264088083	9.16039913489023
H	-0.84402902113503	5.42377008103736	8.77394252179924
C	-0.57092237570561	5.78254055136058	10.88284543165056
H	-1.29355589730565	5.00592984604194	11.15069510988612
H	0.24093516970846	5.75848158268215	11.61473600329750
C	-1.22529414620465	7.13227097347360	11.06584309976531
O	-0.61003549221397	8.10680837893598	10.41353195971461
H	-0.98511072362437	8.97517266230437	10.60855761958622
O	1.83197191225678	-9.06169260561962	23.18736553790022
O	-0.25719265308538	-8.64302417741858	23.83588222691442
H	0.17222937349617	-8.42069546893234	24.67341188454117
O	6.42187711746502	-7.09487234163820	21.15455792768155
N	-1.34713839921339	-9.20392668955007	18.32074805335207
N	1.51106306287156	-8.02481883968639	19.42377999697114
N	1.72377211135938	-7.66151333602986	20.58994249986339
N	1.39450420908349	-4.77445316757378	18.82607149897236
C	0.65150710382370	-9.08941204747455	22.95813208218512
C	0.00650561829354	-9.61102183543532	21.69736444197828
H	-0.69666331887881	-10.39121139459637	21.99721065822328
H	-0.58506801466652	-8.79312593852209	21.27507382773630
C	1.02882778986530	-10.15286964817087	20.69122684195230
H	2.03402240752862	-10.08423186252831	21.11478482484615
H	0.82198931259689	-11.20529749628949	20.49460962525366
C	1.03991869082434	-9.42547252663680	19.33842502277290
C	-0.30257580149384	-9.32438360569066	18.77589321282706
C	1.96018387200810	-10.15184087472115	18.35092684834292
H	2.98814880619889	-10.04874292732444	18.68737854352050
H	1.70154598685518	-11.20700337968644	18.31722746626296
H	1.86655475754681	-9.73141851378184	17.35130215385961
C	1.82450642548855	-5.44031646755539	19.65611236805281
C	1.63868783082473	-5.76536328457324	22.06440959977132
H	1.90899193159555	-6.43353181907047	22.87803679574566

H	2.03344273708179	-4.77374571946451	22.26985190622301
H	0.55334556775851	-5.71546236779821	21.99464898256059
C	2.23775186183384	-6.30407244000074	20.75875128508213
C	3.76540111436216	-6.42175030039684	20.91989556824425
H	3.95119458324055	-7.03582955513179	21.80455251408794
H	4.16891974121160	-5.42698856661194	21.11634975246489
C	4.48379404293839	-7.04547446100624	19.72654599976086
H	4.48280425638425	-6.38177236495876	18.85807991722645
H	4.01044864353097	-7.98322697855477	19.42382260443464
C	5.92015519276339	-7.35360525710725	20.09081267125812
O	6.57591737947875	-7.95596934708429	19.10427599014647
H	7.47981103835335	-8.17018985321402	19.36960273742384
O	2.91019910663049	-0.94778656427799	11.25459267536100
O	1.94913970509501	0.57047830086082	9.92778727243227
H	2.05660668499338	-0.13023164712165	9.20285521843450
O	-0.54292107147365	3.60374120429559	18.61675855184350
N	0.97312602485217	-1.86057849070208	13.50346606684696
N	1.38489466414104	1.13147492965585	14.88775551649104
N	0.41252561422381	1.27279186711019	14.12643891132629
N	-2.83133086718476	1.22556607539277	14.40683035569655
C	2.48665867242401	0.17001993845742	11.06108696193430
C	2.53970165158442	1.27752666674216	12.08823190714037
H	1.52130939832710	1.63285516765726	12.28076847568547
H	3.08038392098058	2.10512983580419	11.62941144863324
C	3.24941004693071	0.86575839023709	13.37157525834519
H	3.64546035911086	1.75442152557369	13.86273793184100
H	4.08910274203294	0.21214113619770	13.12648857241611
C	2.36329052506334	0.14272768276360	14.40538272174705
C	1.64076093829197	-0.99811938764187	13.85474840655083
C	3.22890197310761	-0.27206126150812	15.59636935426555
H	2.67586046888562	-0.91419657084158	16.28053014880603
H	3.54458496630712	0.62337469022096	16.12522357032372
H	4.11624314788231	-0.79674590673188	15.25409278179909
C	-1.82851280168814	1.77449761098641	14.47497774377932
C	-0.35474268485047	3.43028059590343	13.42782949448874
H	-0.42529050248686	2.97105984154663	12.44147064372712

H	0.61429445292950	3.91271739369950	13.51961128174071
H	-1.13722362087481	4.17985745460358	13.52435660398164
C	-0.49377447998246	2.36957607774807	14.52401406436401
C	-0.16046055307729	2.91682017797137	15.91574395557995
H	-0.33453253126536	2.12918053827327	16.65555835494537
H	0.90296897586684	3.16116541527602	15.93484382664148
C	-0.95873629180250	4.15770932627073	16.30587956471668
H	-0.63741255972645	5.03715168113318	15.74890960976909
H	-2.02803352990086	4.01636391927156	16.11777704697492
C	-0.80294107052669	4.43817652836947	17.78460558624820
O	-1.00165473207420	5.71695664234500	18.07516473396011
H	-0.82739463013066	5.90882880398113	19.00493321889482
O	-10.34739140941553	-3.26532542578307	17.56435889038232
O	-11.92271010391020	-4.21660171959190	18.82831429490035
H	-11.79282420194441	-5.02542104008407	18.26806580636117
O	-5.08184650507568	-0.29321961322446	24.63299327458505
N	-8.53034129245619	-4.17933903227667	20.00563442927535
N	-9.19223980885707	-1.11820507870624	20.91649182540349
N	-8.33018815726677	-0.78554548748073	21.74405392104860
N	-6.66268429771396	0.28899882318804	19.05458227153994
C	-11.15244149836625	-3.20882419497483	18.46523450048787
C	-11.42296755961035	-1.96905978530250	19.28297955733655
H	-10.73928108699509	-1.17121901878090	18.98577121785150
H	-12.44215035609177	-1.65547011041104	19.05068048164229
C	-11.33551769472684	-2.23259505512450	20.78846050141169
H	-11.78491314524744	-1.39376000546973	21.32021218545235
H	-11.89190068923777	-3.13614260409082	21.04401434552480
C	-9.88962311332374	-2.36868759300233	21.29214297484717
C	-9.15928348535092	-3.41461087081380	20.58494349737717
C	-9.86693712831017	-2.59773653092656	22.80360224953962
H	-10.22602702553157	-1.69876821809583	23.29859214997250
H	-10.51576138886793	-3.43096712878701	23.05857462167641
H	-8.86049003975293	-2.81009199050712	23.15562416526253
C	-7.13719874738872	0.42422584369854	20.08907731860844
C	-8.82851182364819	1.57025868316300	21.45107686595124
H	-8.42084607436430	2.55697470648161	21.24683460609577

H	-9.57930650590865	1.31664280692038	20.70465317121931
H	-9.30242548692066	1.57447504552925	22.42831702674799
C	-7.71772141468548	0.51943074939263	21.42188362004443
C	-6.60591070793922	0.78904206109266	22.44593899372786
H	-6.17189858935838	1.77151279821457	22.25320585874360
H	-5.81979244516624	0.03785493759682	22.31877870225039
C	-7.13073244413556	0.72269834076884	23.88253327110827
H	-7.83641234127059	-0.10877780711107	23.97722613922177
H	-7.64684349225301	1.63752676434400	24.16976229940898
C	-6.00677186399998	0.44709054204581	24.85680339591293
O	-6.16589219390873	1.10074748385213	25.99852717527984
H	-5.46778972932072	0.92410773225747	26.64050045843745
O	-12.19117087715404	-3.89492435825460	10.30128805218491
O	-13.35928940286601	-2.51694598533337	9.00118153154227
H	-14.12250413231156	-2.78948779618200	9.53129346615510
O	-5.09075103672374	-3.66175213549340	6.34307964193257
N	-8.03786921068374	-4.76574516472158	8.39897527724230
N	-9.45863854718745	-2.83690264358314	5.98533811809079
N	-8.30120296853944	-2.44929451464730	6.22727090625492
N	-6.33796836808835	0.21673321849957	6.81942332465375
C	-12.25212993603308	-3.15191377174199	9.34292817261239
C	-11.10691384574972	-2.89755860071322	8.40238750505830
H	-10.16978786935948	-2.96315480847829	8.96290078685250
H	-11.19399466934643	-1.90801400046087	7.95215986290661
C	-11.15545114549407	-3.98338309947078	7.31315272151776
H	-11.92062938472732	-3.73129972955624	6.57947968887003
H	-11.41171382767718	-4.94847481870812	7.75736930318631
C	-9.80295247181442	-4.12429554181715	6.59737731983320
C	-8.79663038156213	-4.52800488287319	7.57398077003758
C	-9.90426496373862	-5.11413089702272	5.43280230781722
H	-10.62536021682852	-4.73857308315354	4.71074231541257
H	-10.22514659373307	-6.08927642935839	5.79202066796428
H	-8.94097438366601	-5.20756336762300	4.93705623785628
C	-7.04360168723835	-0.43706441272654	6.19278932064698
C	-9.18323570177257	-0.44870176197152	5.01840664371584
H	-8.86777070685991	0.44034619713354	4.47887543711852

H	-9.74368333182333	-0.15235128801023	5.90434965934262
H	-9.82427151956765	-1.04778219371095	4.37693415175235
C	-7.95558768218428	-1.26923864604635	5.41681268933977
C	-7.25568700140999	-1.84420004109315	4.16726229977768
H	-7.90665399546783	-2.61588527548068	3.75152103136012
H	-7.15663900785472	-1.04950602459175	3.42839403366457
C	-5.86654380898310	-2.41799348200397	4.44986684714381
H	-5.45524456860703	-2.78465118144268	3.50617361646490
H	-5.19324941164425	-1.64609054237158	4.82917686951677
C	-5.84200418107376	-3.59328338469828	5.39517814937771
O	-6.66090731838032	-4.56876479310575	5.05789681822377
H	-6.50714043236227	-5.38634398300190	5.62022922447251
O	-11.46850394995808	-5.33589372869879	22.12005895179191
O	-12.34922249588582	-7.29174124545633	22.73076063605624
H	-13.19698136708692	-6.82904739576261	22.73028134769761
O	-1.34185614334298	-6.75527586638098	21.07817542238170
N	-7.13385094832925	-8.05309308142257	20.39402679174159
N	-6.51889607461806	-5.73175494259608	22.77027732366038
N	-6.29598144445635	-5.17841349600719	21.67894065598920
N	-5.05603433217151	-3.02473967037778	19.46968595939347
C	-11.33353482954029	-6.48882947160204	22.44688312701520
C	-10.00453652388297	-7.20214345481912	22.56858557305876
H	-10.06758366425817	-7.92222092454322	23.38390372830626
H	-9.86282858657055	-7.76448560681947	21.63983307978677
C	-8.86756116042982	-6.20401671148317	22.77538236282055
H	-8.93059046335317	-5.41712811734365	22.01830407440513
H	-8.95905977520805	-5.73546690817950	23.75625629220317
C	-7.47208456286862	-6.84703464038992	22.67778082111335
C	-7.30609404142119	-7.53068844018690	21.40112931933174
C	-7.17272623912797	-7.80135317384141	23.83327091646475
H	-7.85126480485819	-8.64981012066817	23.81310452218951
H	-6.14884586210561	-8.16359782679425	23.75797873879196
H	-7.29122976281737	-7.27059207830057	24.77440208023615
C	-5.17957646349158	-3.44981155736711	20.52735849742826
C	-5.91045003126772	-3.03564741263768	22.83861822621543
H	-6.79639057535451	-2.55339325253044	22.42769972951631

H	-6.18291745409208	-3.55379432936230	23.75485784560134
H	-5.16854626394332	-2.27375717971880	23.06817701077652
C	-5.34516854249169	-4.05170324478095	21.84731824674581
C	-4.01350800864583	-4.64067548957097	22.34674775499745
H	-4.22530712669700	-5.18985849418017	23.26555766528121
H	-3.31868804508069	-3.83314384993385	22.58124128386417
C	-3.41344981184356	-5.57512169104013	21.29791769870093
H	-3.07561902830488	-5.01591245707163	20.41971300424871
H	-4.17634345478841	-6.29399348728601	20.97208885680904
C	-2.26004240033227	-6.41541529361717	21.78893396799710
O	-2.38883725890409	-6.80401448686242	23.04773483200426
H	-1.64252565752460	-7.36546673022575	23.33714736282441
O	-11.22765708158065	-9.57661702426596	18.14841599789273
O	-10.24787352682414	-7.88121749822800	19.20790144745398
H	-10.83192803845309	-7.33366449658482	18.59528938159014
O	-3.92504373974545	-13.55538661494709	20.12761254240428
N	-8.41485681930883	-8.99932136420016	16.69003001893526
N	-6.88518407139628	-10.91885742264376	19.02817457313241
N	-5.98728236910654	-10.23071068807925	18.51554034221098
N	-3.87014270267012	-7.78513738264886	19.27039061044241
C	-10.44990894990643	-9.15568895658762	18.98900052628987
C	-9.66237608108576	-10.08315530449487	19.87758789861435
H	-8.79191375861504	-9.57141077650556	20.29401263623313
H	-10.32466611119037	-10.35206244162983	20.70254187466760
C	-9.26091721034669	-11.35069380607399	19.12333285799066
H	-8.96427229475337	-12.12007937491332	19.83615361719970
H	-10.11406338385495	-11.72021748120501	18.55181607864822
C	-8.07126366644624	-11.10801137482844	18.18229394872177
C	-8.28501115125957	-9.94505185082043	17.32655746639555
C	-7.78961632675093	-12.36476044489433	17.35035410290175
H	-6.87705206428829	-12.23090360658638	16.77162954987211
H	-7.66162732975331	-13.21262642396078	18.01886728252399
H	-8.62358142428947	-12.56265196735568	16.68127953242495
C	-4.24190523999327	-8.86930535867908	19.32579281999066
C	-5.05592823441114	-10.65062444965571	20.78940023827771
H	-4.16693310657680	-10.53727483096695	21.40503974309589

H	-5.85062150218805	-10.02587584223271	21.19495033352329
H	-5.38300215063566	-11.68664309998915	20.81588523509278
C	-4.76414744925295	-10.23195307069733	19.34993154897396
C	-3.81893951904081	-11.19027288559708	18.58514198045048
H	-3.47190931796858	-10.68667573529135	17.67510574477065
H	-4.41133842990025	-12.05908836586603	18.28459833175080
C	-2.61273019085496	-11.66563348611128	19.39430924654751
H	-2.24768648793314	-10.90250917425836	20.08398665351827
H	-1.78546270770644	-11.89973062388361	18.71764176901051
C	-2.89597366581913	-12.93010597643880	20.17730990265218
O	-1.85756972827678	-13.29678652159815	20.92074519516077
H	-2.03072691915628	-14.12969366134704	21.37779929603190
O	-4.59504205097664	-10.50797874527068	13.92954450255462
O	-5.97514852126497	-10.26939456631841	15.65309023099246
H	-5.22818306037245	-9.68229346241699	16.02594722803956
O	-12.14305785022816	-6.88446293503893	12.10531838798651
N	-4.98905410578292	-8.48258182072818	12.04018362575014
N	-8.14737113381061	-9.37968587202048	12.44596165203154
N	-9.00138877044633	-10.17098374361948	12.88459027502754
N	-9.67305434955219	-7.17439181430772	14.31845442406765
C	-5.65457576258737	-10.74780753828021	14.48142978971140
C	-6.72210889378349	-11.61950151040128	13.87847546376801
H	-7.68347189004136	-11.38979134542742	14.34130283233533
H	-6.47370180114252	-12.65113348659356	14.13651739667070
C	-6.79385647987127	-11.51040220402083	12.35660831397621
H	-7.61431741046544	-12.13957298594272	12.00924771260120
H	-5.86752652937308	-11.89092432724984	11.92180095867518
C	-7.02228449874881	-10.09121467294421	11.81041370078499
C	-5.85501396015917	-9.22985945282388	11.95335061472080
C	-7.38275570020060	-10.16386999579953	10.31947075335642
H	-8.38399763416028	-10.57160038411008	10.22281832399620
H	-6.68690616938118	-10.81919822203279	9.80082499133836
H	-7.36088250310346	-9.17728616154028	9.85935348592244
C	-9.95280599989713	-8.19560311882441	13.87836527284067
C	-10.80758401702418	-10.41421136893176	14.47133381905637
H	-10.16078138646745	-10.39898873103520	15.34874634183632

H	-10.88732527409678	-11.43549974117342	14.10663732338585
H	-11.79584208222069	-10.06034058655655	14.75383939526840
C	-10.22896179921732	-9.53226805905774	13.36344392329402
C	-11.16458072877255	-9.52285527586608	12.13597816481430
H	-11.15906082374144	-10.53449049286183	11.72523069167209
H	-12.17904018132890	-9.29167370709390	12.46635224934270
C	-10.73839631473219	-8.53320324804885	11.05483644935529
H	-9.64830718525450	-8.47204267326597	10.97019201703301
H	-11.11155881971717	-8.86776555802668	10.08501600820091
C	-11.31153049613208	-7.15054281337150	11.26782692437351
O	-10.82897184995513	-6.26654750783764	10.41172372219685
H	-11.34771120075661	-5.42116435688143	10.45237093909441
O	-4.18365762149061	4.12466288997188	15.81700878693032
O	-5.78724127006737	5.66220622445428	15.99629790328401
H	-6.24537202593353	5.01895254950636	16.57811224787275
O	-8.43469047120220	2.20386412189842	9.29609220134405
N	-3.41589169575930	4.06644269613656	12.29185670932291
N	-6.40324538893136	5.59248538191554	13.08598303300769
N	-6.66810518763444	4.39353837482857	12.90655308259781
N	-8.04739946919129	2.48892331863181	15.29569906617804
C	-4.59915389661321	5.23816236542127	15.59504501682411
C	-3.80477643060090	6.29791078944082	14.87572889191141
H	-3.53829707704110	7.04637253002000	15.62449924218315
H	-2.88113138871870	5.85575094658451	14.49631417154400
C	-4.57130423763383	6.99991249539953	13.75365194985771
H	-5.43909880152548	7.51850457923502	14.16425390781116
H	-3.91142680961131	7.73936314933518	13.30287835769051
C	-5.07933437170211	6.04003144732217	12.65982577427615
C	-4.14848678782900	4.93867374339987	12.43456118974161
C	-5.33414872219886	6.80054881376373	11.35178096268926
H	-5.71119442015717	6.11867963812562	10.59082518472687
H	-6.07392883677679	7.57584593568861	11.53316145844203
H	-4.41340174139248	7.25787542597793	11.00025846908804
C	-8.05683233273618	3.20036211044327	14.39290291638267
C	-8.85654616091128	5.36025653627025	13.65972344959010
H	-9.88996284234142	5.09970538808693	13.87757406256575

H	-8.40724109741066	5.81244928248499	14.54195623503851
H	-8.83123405642387	6.08675134156307	12.85167892586898
C	-8.08095083467964	4.10501878262906	13.25653864869687
C	-8.70571023484149	3.37112978850400	12.05498759680741
H	-9.77855578873359	3.27872420293274	12.23074512690987
H	-8.27965788659276	2.36398541763067	11.97605259559537
C	-8.45900656874377	4.12743602569305	10.74961549855662
H	-7.38664563663026	4.31612068129465	10.62868055489362
H	-8.98177900603186	5.08293375398887	10.73659481749406
C	-8.89487692661466	3.30527370769313	9.56543859514660
O	-9.78483359676167	3.90779464124218	8.81995405171515
H	-9.97191213171593	3.42956316984653	7.97198386134471
O	-11.00341556187178	1.87371734333501	10.80188306252918
O	-10.39294247672145	0.22073476692341	9.43841194724127
H	-9.86018610797483	0.93558152255458	9.01940901013966
O	-11.51256677907864	-6.46913621377463	17.35283421270234
N	-8.90879443518509	0.33467780196644	13.01547236655146
N	-11.35956227469944	-1.89973317113125	13.42367129031088
N	-12.01366227733481	-2.46922040775864	14.31463307314993
N	-10.05098207285204	-4.69090736690470	12.84020840423750
C	-11.07678891873917	0.71755793990075	10.46560441487082
C	-11.97177455446333	-0.30091170906453	11.12889048199219
H	-11.51591727531793	-1.29382986990134	11.08240503728262
H	-12.87930745558953	-0.33193504818800	10.52185147831515
C	-12.33736434369183	0.09089724512555	12.56201526855229
H	-13.31294294889506	-0.32388985850466	12.81619841926120
H	-12.40088789878745	1.17846594917420	12.64116997404506
C	-11.32824578470624	-0.43697575606751	13.60046330456202
C	-9.97787444416729	0.01004175899099	13.27285483615381
C	-11.70850126997894	-0.00813374512112	15.01653911177117
H	-11.06545679798625	-0.50077301270404	15.74856283631433
H	-12.74121095083222	-0.28438307086330	15.21552042877374
H	-11.60980308026176	1.07039103480494	15.11778797218070
C	-11.04459669867815	-4.39814909788648	13.33631490557939
C	-13.47591591946180	-3.99569443918885	13.19011729888985
H	-14.33544920994331	-3.69785042271181	13.78417281108078

H	-13.61490968723398	-5.01168387060440	12.82915162193606
H	-13.38531826343904	-3.32553562237764	12.33803027737359
C	-12.21292272930486	-3.90338936064760	14.04686766112373
C	-12.34665810978002	-4.61873198954058	15.39768970279694
H	-11.41848233390643	-4.47015479684733	15.95917414032782
H	-13.15424421863604	-4.14557568523671	15.95764728797748
C	-12.62024092468005	-6.11255164040591	15.23653946001854
H	-13.68567537352719	-6.31397755215727	15.12267854475664
H	-12.13171262523109	-6.51216679290929	14.34118799992204
C	-12.12288832972767	-6.93367186748687	16.39508531291050
O	-12.39214649587061	-8.20047397972321	16.27003536571332
H	-12.06090972791066	-8.74445116989097	17.05737559985547
O	-4.18076355526944	-8.54114822848326	16.38164479192161
O	-3.22278785652674	-8.21754204119925	14.39551474253209
H	-3.78569123455662	-8.99020683783183	14.10857380837800
O	-8.88038638760744	-1.57648805910295	15.41125357653087
N	-2.77656289307335	-2.73477694853362	14.41628831601392
N	-4.32236214989153	-5.28319872709354	15.88749659924161
N	-5.06320592369782	-5.01333861923713	16.85161163984208
N	-6.68009915790389	-6.72664238186057	14.41646226088702
C	-3.31129556793954	-8.03319710345016	15.68892674266668
C	-2.20821039504255	-7.18994869897355	16.27177604454035
H	-2.39646753252802	-7.05574604479905	17.33872009498803
H	-1.30090317865081	-7.79563210153946	16.17277263742524
C	-1.98533302031099	-5.85768467894514	15.55736542804054
H	-0.96294116651480	-5.52140367964686	15.74904468597812
H	-2.10027404862604	-5.99155095752703	14.47267147976302
C	-2.95067787206696	-4.76088045628469	16.04883131850097
C	-2.84911221914726	-3.62156172971233	15.13875940636522
C	-2.64872361742612	-4.29571945627155	17.47340114159760
H	-1.61232365889828	-3.96651232880312	17.54377553386790
H	-3.30231978351215	-3.46994549493694	17.75757824879121
H	-2.80950296071502	-5.11165507827859	18.17635937700426
C	-6.59926971612385	-6.22001892758061	15.44295438255951
C	-6.59169549331919	-6.55998011562096	17.87328584724255
H	-5.93385551307675	-7.41614355307618	17.72930692939685

H	-6.34832053492489	-6.07943917443582	18.82074383842126
H	-7.62173266373105	-6.91153431035246	17.90971212576208
C	-6.42057672175827	-5.55525420020422	16.73232445755665
C	-7.40820321957985	-4.39042363162794	16.91575805457463
H	-7.22107433201121	-3.95007468867642	17.90010453627558
H	-8.42585411397278	-4.78952807232255	16.92144220378715
C	-7.27286600182551	-3.31983918636951	15.83243169621905
H	-7.59195368156491	-3.70096296299736	14.85700500960667
H	-6.22499603399695	-2.99194396447307	15.76358539776363
C	-8.10514529612250	-2.09635924329063	16.17758303414103
O	-7.86067752417850	-1.64783556238472	17.39449130218200
H	-8.45843322658423	-0.88460596596317	17.61482345493457
O	-14.59102065041217	2.05314501334007	13.20409464454413
O	-15.35311914951197	3.83701781945610	12.09898047722630
H	-15.27722293890095	3.29099175775888	11.30268603986850
O	-10.49620616418087	6.26342855260326	17.64593960573639
N	-11.55797057573774	3.21714507123314	13.70598888055924
N	-12.48042313319188	3.76530026209561	16.90161909161563
N	-11.70918035476781	2.79374476661960	16.83763747296744
N	-9.47366171548530	0.53030825781975	17.84640424185224
C	-15.03456320995829	3.17409186113194	13.20444191109862
C	-15.34240757752345	3.97700486144250	14.44416560625697
H	-16.38713561110525	3.76058533968627	14.68096430567245
H	-15.27999154474882	5.04109060665804	14.21931175316280
C	-14.48063332236229	3.57966461590370	15.64020754847597
H	-14.40522447896281	2.48941365058490	15.69132737229083
H	-14.95392906326238	3.92920378590434	16.55850636020231
C	-13.05535918069020	4.16928109575432	15.61294916153068
C	-12.25400375786327	3.63720438768080	14.51554158166335
C	-13.03670671395993	5.70052297873886	15.59006228907035
H	-13.35972835987882	6.07541665815173	14.62240360636107
H	-12.02580909228793	6.05313702689698	15.78818750211111
H	-13.69803264043392	6.08043477720923	16.36485355995910
C	-10.23580736386443	1.36980562860445	18.01301772042007
C	-12.39902148339930	1.93131739651585	19.01588529363611
H	-13.13685193883969	2.72694238472664	19.08183762037135

H	-12.07021119822950	1.65901386425913	20.01551518788713
H	-12.85317741306031	1.06629845953808	18.53579331538408
C	-11.21458090845869	2.43585150755226	18.18785309157584
C	-10.56521176389677	3.67530696096583	18.82149772154860
H	-11.32867086412009	4.45615935952384	18.85611930629507
H	-10.27378138303000	3.44714322240092	19.84550093238364
C	-9.35640220057761	4.16648205995239	18.01639567426463
H	-8.42387645897727	4.03516044851852	18.56884952424732
H	-9.25911957352635	3.60742929065268	17.07800018897693
C	-9.48045432085966	5.61761688364603	17.61835556417713
O	-8.32105797310481	6.11876105190593	17.19017023384541
H	-8.41890942744922	7.06304558572774	16.99796780518527
O	-5.43868627137170	1.33306902109046	16.28027829258973
O	-6.52727498451616	2.98904807118550	17.30810957347553
H	-7.21816194992756	2.80625116492085	16.58680960955759
O	-1.08970467587859	-3.70510145696602	21.85442919942682
N	-4.62901199043757	-1.44659409859078	16.81125174238512
N	-1.85197764382359	-0.51021269482213	18.16942799999920
N	-0.95783555455534	0.23218353077160	18.61432801161080
N	0.35833765203455	-2.01634333288870	16.56411861193496
C	-5.50531009124326	2.15062783089402	17.16186590301841
C	-4.46275587721000	2.35386929017903	18.23633128563654
H	-4.22937278380173	3.41947042118427	18.25672191318646
H	-4.91943977735383	2.12101328231271	19.19872044487581
C	-3.17652229506738	1.56980483297395	17.99747491061711
H	-2.88745260695524	1.66693822772188	16.94416739935216
H	-2.38874231941824	2.02161239840207	18.60182746333934
C	-3.20444376544767	0.07696829247455	18.36947742029950
C	-4.06962702110276	-0.72923818761716	17.50817294386134
C	-3.51899501696855	-0.15523846585401	19.84963607917263
H	-3.46716137727766	-1.21746328399921	20.08751167015879
H	-2.78164013303736	0.37580099484979	20.44643919318934
H	-4.51038388350843	0.20859092694814	20.10221045602021
C	0.43858474409126	-1.31113314823708	17.46464159925363
C	1.40086423186239	0.76141555140650	18.42550500707906
H	2.39384768966010	0.40861768151223	18.69092905938548

H	1.42413001673893	1.13015463180297	17.40199006987791
H	1.12451111167289	1.58000579033744	19.08655984779198
C	0.37998021714809	-0.36556674715010	18.57480070945062
C	0.56473695234628	-1.06801216149032	19.93369720947584
H	0.31685531726503	-0.34839574042943	20.71569927693011
H	1.61506562533361	-1.33824581769371	20.04787198604648
C	-0.30701832177586	-2.31152489829374	20.06840179689025
H	0.04934442734513	-3.09876034929965	19.39119682635332
H	-1.34890746192207	-2.09592693378951	19.80810233625717
C	-0.28117436152305	-2.90177134529710	21.45761379704135
O	0.73744442287680	-2.47337158692483	22.19264813675304
H	0.73407111143429	-2.85377597457244	23.08206752885279
O	-1.78592200861485	-3.10505589421912	10.79509954440072
O	-0.33826315296117	-2.56554991320117	9.18045299846203
H	-1.09712368360336	-2.29501175241713	8.61973134274811
O	1.16018306955278	-7.95211726725554	15.95086770886049
N	2.81288186972118	-7.09306482547746	10.50857849794457
N	2.88282486753530	-4.23548242542757	12.30518661305872
N	3.35276901445950	-5.16445671547761	12.98505827521078
N	0.96332274271840	-4.52744076367994	15.17410751452201
C	-0.67849273524989	-3.15541380214255	10.31549111026818
C	0.46952195373190	-3.90497814207986	10.95489445491138
H	0.15202233735653	-4.95067518646385	11.05425760366632
H	0.60712741056753	-3.50668826208418	11.96883207444712
C	1.77194720276319	-3.79651407478151	10.16231338595988
H	2.04140659609121	-2.74476259204766	10.04449536334886
H	1.65066133641366	-4.21256743431786	9.15893958285127
C	2.93919016036214	-4.51231067370009	10.86505935906144
C	2.88609907074986	-5.95399824751613	10.64240248813845
C	4.29537773763511	-3.96059202421491	10.41265571907123
H	5.08990320982058	-4.48858680342215	10.93473447486912
H	4.35261543712271	-2.90078870078762	10.65605266603283
H	4.41484573627042	-4.09067184297948	9.34029681531955
C	2.06748328064419	-4.64615100044346	14.89293674860781
C	4.25214323928430	-3.54679484137216	14.57311204191148
H	5.25611767635389	-3.71937151656738	14.19450740857049

H	4.31394468274001	-3.25674437707594	15.61898963970510
H	3.79292610769679	-2.74821158511232	13.99049125809253
C	3.43460472684843	-4.82920319769563	14.42461219007923
C	4.12235167687941	-6.01340121361929	15.11412299493720
H	5.07244936482366	-6.17400065273656	14.60360956586972
H	4.33943900856851	-5.76586736971994	16.15402590634368
C	3.28637947839178	-7.29155059064823	15.02736965779996
H	2.82760194456791	-7.38914835393864	14.04093373703003
H	3.94488223019341	-8.15050585825533	15.17497326688212
C	2.23958132541915	-7.42609892455505	16.11045519156462
O	2.66247028027995	-6.97961277431389	17.27789514301032
H	2.10559398006222	-7.32003842267409	18.06209985191966
O	2.51414735341954	-12.67067972306170	11.00986906717312
O	4.05236823226400	-14.01868578655110	10.11769453419278
H	3.96272913412640	-14.55093774843715	10.91871757462326
O	-4.24629143970456	-9.30359343859994	5.12789667434756
N	-0.44510467740829	-12.75828893614009	9.96646855060130
N	0.24943239298737	-11.09376101934929	7.24995024795511
N	0.17986064918883	-11.36350878740261	6.04063249108588
N	-1.39050464781997	-8.36884406740113	6.59601220098880
C	3.27972750522978	-12.93720742453356	10.11910721210169
C	3.52333179878774	-12.09331335567108	8.88969729430691
H	4.34102259575401	-11.41379316443788	9.14809440386348
H	3.88329457505006	-12.72985754439375	8.08261375916027
C	2.32279153328624	-11.25302635413736	8.45853067748214
H	2.02066987187828	-10.60390713537745	9.28682598638058
H	2.63011276083485	-10.62056608774090	7.62344664487912
C	1.09748742840650	-12.06929087526126	7.98344134968206
C	0.26504812958870	-12.48062834653206	9.10997796180587
C	1.45469506143770	-13.28137820110470	7.12459796690538
H	2.10251858405883	-12.97833282597141	6.30592751873525
H	1.95727126184969	-14.03275967828671	7.72865640986631
H	0.54687939686405	-13.71168806651708	6.70629017915482
C	-1.09284975696185	-9.30086451869495	5.99579497117569
C	0.14033655053317	-10.05287429227243	4.02276933511824
H	-0.43318684077130	-9.36786822632126	3.40324106911296

H	1.05217898334625	-9.55738600885566	4.35115015422463
H	0.40444558950456	-10.93272674641011	3.44198723365198
C	-0.67828709149415	-10.48004049152511	5.24408737980431
C	-1.88074670679345	-11.37812143947830	4.88509761920928
H	-2.42744433606647	-11.60804615839963	5.80246024777276
H	-1.48206306714346	-12.31168704047764	4.48592599168769
C	-2.83640286987261	-10.77779659725313	3.84875985438673
H	-3.67862921021413	-11.46248964484300	3.73654700302246
H	-2.34059063097852	-10.66885784035517	2.88543853736109
C	-3.38218025376373	-9.44047421669521	4.27604159848654
O	-2.84811886750866	-8.42776520615965	3.64161194752983
H	-3.37109012005869	-7.57584329992911	3.81941745207564
O	3.57234666250293	-4.20641742762411	7.14287520723377
O	4.88089222565054	-2.63620228945376	6.25023889007346
H	5.42647638339352	-2.73209788388555	7.04522505320441
O	-2.12655243755382	-2.75777224822449	7.11692493509872
N	2.04487266077974	-1.22440272169021	8.01363302324590
N	0.48162779670811	-3.20845365563021	5.67740039340187
N	0.54646616369236	-3.83941946383631	6.74468482162926
N	-1.46475292879258	-5.53262625923946	8.85392536665939
C	3.86498935106459	-3.49270859296603	6.21423757573000
C	3.15345061375641	-3.45726651744725	4.88646558881748
H	2.46833029301163	-4.30195993491177	4.81216262105984
H	3.90696644436309	-3.54531131696061	4.10361601823166
C	2.39216795962590	-2.14775507015759	4.66157633212585
H	1.97223007241440	-2.17477924703813	3.65573733192116
H	3.07033108961816	-1.29603713854394	4.73234120122442
C	1.22625671304052	-1.95139704480940	5.65064652345959
C	1.70805124205385	-1.56085501120909	6.96935455194306
C	0.27229126208854	-0.86922246079572	5.13064667300512
H	-0.16635759005705	-1.21117247547126	4.19611217614075
H	0.81548570106035	0.05579583591067	4.95345196766333
H	-0.52623654789916	-0.68686972409112	5.84944360544667
C	-0.86271625630768	-5.36599070422084	7.89193950061440
C	1.04712813065261	-6.16310918167707	6.57736512181299
H	1.55161044298947	-6.03901846522732	5.62302319665427

H	0.67131750667546	-7.18087915582204	6.64361136104505
H	1.76349331082157	-5.98245353126776	7.37778059272992
C	-0.11324950216677	-5.16043716735323	6.65675192517039
C	-1.04218542708384	-5.30840203996967	5.44478178287717
H	-0.52696389469882	-4.91658845549874	4.56698270641537
H	-1.22279182494536	-6.37240731721428	5.27907480440912
C	-2.40363086599344	-4.62416795405138	5.60804232128540
H	-2.93589091165938	-4.70281275398408	4.66102437975846
H	-2.98075477175042	-5.14944447518120	6.37379161207757
C	-2.34822132550551	-3.16534883235575	5.99485109363214
O	-2.60036754998816	-2.36677740462688	4.97816313698546
H	-2.70661556582242	-1.40982837713804	5.27155140547385
O	-3.83395629175339	-11.14040870315954	7.41027734997852
O	-4.79091726255614	-9.52273799530433	8.59701893729108
H	-5.55177128795168	-9.73640375337582	7.98496947145643
O	1.05286989521853	-8.05163766770755	12.78586879923389
N	-1.49581183395827	-6.38920137344371	12.07882003354151
N	-0.86484332285431	-9.67188670927236	11.43966191119371
N	-1.59716413595279	-9.82111505086613	12.43267050599451
N	-1.78701451618367	-10.52766279463815	15.70237525883320
C	-3.77876924088334	-10.31083788577962	8.29374754645490
C	-2.53938278106833	-10.11185109849769	9.13195031586704
H	-2.46802999306472	-10.96265755715097	9.81689246525797
H	-1.68308155544062	-10.18110646596073	8.45881010193676
C	-2.56145903819392	-8.79648094278732	9.90068657258939
H	-2.77016923471142	-7.97388632892004	9.20945221064249
H	-3.35327047599605	-8.83020155919992	10.65735125886401
C	-1.22011940224366	-8.51380489587143	10.59451140453148
C	-1.36542064723749	-7.32395199999177	11.42779256207721
C	-0.09013918647508	-8.32849224422077	9.58412018887796
H	0.25114099571334	-9.30197525210648	9.24341247190562
H	-0.43335363685410	-7.76330302053934	8.72250963318538
H	0.74956951929718	-7.80526520761548	10.03344723842312
C	-1.52944762282488	-10.74808605161296	14.60560702323740
C	-2.12677820644268	-12.15540118405090	12.71127816499350
H	-1.97254355930357	-13.03770303519604	13.32763360168707

H	-3.17431524899996	-11.86248911505383	12.76157821652695
H	-1.87632180910321	-12.39507267194266	11.68083717590272
C	-1.22093878633430	-11.01719481459708	13.20201148518198
C	0.25934892699657	-11.39778162710455	13.04333865793181
H	0.46455380211483	-11.61371875195759	11.99282007698530
H	0.42553449853233	-12.31450086426008	13.61048020687307
C	1.23659957195207	-10.33000121402655	13.55458384161707
H	2.20678667788705	-10.80994781065612	13.69026932770842
H	0.90889296623791	-9.92942868685665	14.51820562386560
C	1.45796424493799	-9.17780137771537	12.59899971462333
O	2.21677454097103	-9.50437560782699	11.56401406182188
H	2.48224698720132	-8.68661025157549	11.04954882275486
O	-6.94531166581889	-10.28851401169750	7.24889367439095
O	-6.02443059717536	-11.35527151143002	5.52053080684010
H	-5.30149393480663	-10.66915597381266	5.57309940236631
O	-5.03901035693609	-17.99291077567691	10.70005565639262
N	-9.82889971170495	-12.58181264329605	10.76680424542685
N	-7.24863672283742	-13.14240735419326	8.75154868374930
N	-6.67767558126760	-14.22480904339721	8.53920699662708
N	-4.44951866783861	-12.28266072284841	10.29561650819317
C	-6.96784224431336	-11.16998160241424	6.41107239159264
C	-8.10250729564241	-12.15508539180253	6.25868884180959
H	-7.67983595616233	-13.14360650099416	6.07453266707317
H	-8.63702324973878	-11.86460267437142	5.35184322901374
C	-9.06128012766481	-12.15153486938204	7.45079768251747
H	-10.07671202940622	-12.34701382813979	7.10711030908098
H	-9.04906680108945	-11.16259285278453	7.91834370018819
C	-8.70405163464887	-13.22791477386559	8.50141205621765
C	-9.34634223975885	-12.87881338944718	9.76801320398248
C	-9.15533042040212	-14.62257436234393	8.06503647101556
H	-8.88779352225255	-15.35865771875212	8.82057015848538
H	-8.67052785715060	-14.89237251438365	7.13010338735806
H	-10.23313806302060	-14.62646447537866	7.92372585567443
C	-4.78738000845476	-13.10808468129729	9.57334271944763
C	-4.61735027998433	-14.06941076293254	7.30994428946364
H	-4.96987270155449	-14.88200920838838	6.68073992635307

H	-3.53088519504209	-14.09814524428813	7.36321374570085
H	-4.92844966683620	-13.12742319320685	6.86504405096125
C	-5.21837490624055	-14.20656446607534	8.71320104847109
C	-4.81759589126796	-15.52553298822112	9.40264300756724
H	-3.72899702331612	-15.56296163986994	9.48810886519560
H	-5.23712059412564	-15.53291726047401	10.41357479238417
C	-5.31980047831671	-16.75716271119804	8.64948859585910
H	-6.37592074283541	-16.63995595932119	8.39037791856598
H	-4.77231077889503	-16.92249113485344	7.72131237958142
C	-5.20346544755911	-17.99661508046843	9.50688515120034
O	-5.31819951148661	-19.10649956480072	8.78723070617564
H	-5.24690247652587	-19.90442518621957	9.32512484291085
O	0.92372830997240	-4.14875837603495	2.62158819687112
O	0.43489831665717	-3.98753803886985	0.45169961548885
H	1.37211678411838	-4.17257763257139	0.29852217690039
O	-9.50775624510953	-3.86850028982511	2.38490268236526
N	-3.32803248107496	-0.73154130006991	2.76771743507492
N	-4.15290989571580	-3.88250004766515	1.81474775927142
N	-5.07689213477252	-4.38318326247775	1.15101860939935
N	-4.38808664415209	-6.36782737089759	3.87063592042586
C	0.12056141020460	-3.96339377648403	1.73813923621183
C	-1.34139810002751	-3.68527987886435	1.99671978375338
H	-1.40030985826260	-2.95497955951679	2.81010467597224
H	-1.78735739079658	-4.61391795359198	2.36588907452693
C	-2.09953295125637	-3.20044428090789	0.76047471507346
H	-2.17270544341800	-4.00712644778961	0.02950636761891
H	-1.57126947994606	-2.36620565924219	0.29648689453606
C	-3.52851848774210	-2.73893008592277	1.11840049282213
C	-3.43654829500201	-1.62512892787369	2.05514847248028
C	-4.30226981547543	-2.29526760910585	-0.12357787160606
H	-5.32208679819917	-2.03148369431346	0.14901024843964
H	-4.33032098162900	-3.10689970143035	-0.84640174638813
H	-3.81224327698198	-1.43244590916090	-0.56734362334019
C	-4.95413785596508	-6.02018840263768	2.93210329248127
C	-5.43687901102219	-6.69817245009049	0.65206142497011
H	-5.91640774426576	-7.62312469487648	0.96274509213888

H	-4.37187248500327	-6.87164299907404	0.51415548082918
H	-5.86319256815286	-6.36654798990104	-0.29074564684344
C	-5.64796407847345	-5.61094687473334	1.71580258899433
C	-7.13554877461798	-5.36329463179677	2.02681728052977
H	-7.58686480060606	-6.30310787645761	2.35203206090107
H	-7.20125015514305	-4.65185470728697	2.85257286074676
C	-7.90362627298555	-4.79760324157920	0.83430795931059
H	-7.34509860961634	-3.97219587794877	0.38374065681409
H	-8.06899254154589	-5.54480369631787	0.05828309020520
C	-9.23869580210440	-4.23490373485977	1.26712979468096
O	-10.08682530613242	-4.16826037274155	0.25080644719436
H	-10.94077945827544	-3.78718423320275	0.49469155019990
O	-6.08550120838501	-6.59226113094217	6.66295960258747
O	-4.00070559393096	-7.29768767795913	6.99528666517662
H	-4.16510118776183	-7.85141772305577	6.19414381881938
O	-7.59024830973669	-2.79023630437528	12.62786648472168
N	-4.25148868330561	-5.58551827587153	12.69501646743395
N	-6.28202218613859	-4.18612852785717	10.53666886588616
N	-5.19028064654165	-3.61473261092388	10.37308761614714
N	-4.63195829908271	-1.20230471668229	12.38825193541071
C	-5.06577529077513	-6.60473116770449	7.32752953936946
C	-4.91988499034652	-5.83792938204648	8.62325174274380
H	-3.88086212820922	-5.84912952665148	8.96676240341295
H	-5.22028574928878	-4.80620257474830	8.42121538293148
C	-5.87459708743498	-6.46121353584919	9.64179161654406
H	-6.83498665589420	-6.62650693728342	9.14913849807692
H	-5.49518593948628	-7.43527287917512	9.95790307372776
C	-6.13345346814529	-5.60670583290711	10.89755556291533
C	-5.04836865164548	-5.67066297053702	11.87479354531313
C	-7.45214368187666	-6.03831305205941	11.54322566296547
H	-8.26218726987640	-5.86064362680117	10.83798134749380
H	-7.43073320035221	-7.09797327210642	11.79198445344960
H	-7.64470372412628	-5.46577947529289	12.45269589688228
C	-4.94220379445768	-1.57951273279572	11.35094737308131
C	-4.29627839505689	-1.84331943837656	8.99513096219030
H	-4.53023989840461	-2.39980739550206	8.09284598558770

H	-4.33314264981835	-0.78339303799474	8.75373971208788
H	-3.29249839107434	-2.10911712877837	9.32482499341409
C	-5.32581561637847	-2.17755474911195	10.07218482529452
C	-6.73063045121193	-1.74831060508324	9.62207614253403
H	-7.19480852441299	-2.57486808612358	9.07653711742589
H	-6.61948771701303	-0.91965060234104	8.92307339996305
C	-7.65594597626778	-1.28009592711554	10.74810230643345
H	-8.53262070416021	-0.82920108276330	10.28773229776036
H	-7.16583316946968	-0.52829741534578	11.37327815451508
C	-8.11942663005471	-2.43746909192242	11.59884654650148
O	-9.17796914935131	-3.02386159283479	11.06953017851468
H	-9.54257357154953	-3.76205281285187	11.65498940303021
